# Arg–Tyr cation–π interactions drive phase separation and β-sheet assembly in native spider dragline silk

**DOI:** 10.1073/pnas.2523198122

**Published:** 2025-12-23

**Authors:** Hannah R. Johnson, Kevin Chalek, Nesreen Elathram, Andy T. Chau, Anikin Rae Domingo, Julian E. Aldana, Hieu Nguyen, Alexia de Loera, Brianna A. Duarte, Lado Shapakidze, David Onofrei, Galia T. Debelouchina, Christian D. Lorenz, Gregory P. Holland

**Affiliations:** ^a^Department of Chemistry and Biochemistry, San Diego State University, San Diego, CA 92182-1030; ^b^Department of Chemistry and Biochemistry, University of California San Diego, La Jolla, CA 92093; ^c^Department of Engineering, King’s College London, London WC2R 2LS, United Kingdom

**Keywords:** spider silk proteins, liquid–liquid phase separation (LLPS), cation–π interactions, β-sheet assembly, solid-state NMR

## Abstract

Liquid–liquid phase separation (LLPS) is a fundamental principle of protein organization in intrinsically disordered proteins (IDPs) and biomaterials, yet the residue-level interactions that link condensation to structural ordering remain poorly defined. In spider dragline silk, LLPS is believed to initiate the transition from soluble spidroin dope into β-sheet–rich fibers that provide exceptional toughness, yet how sequence-specific motifs govern this process has been unclear. Here, we combine isotope-edited solution NMR, dynamic nuclear polarization (DNP)–enhanced solid-state NMR, molecular dynamics simulations, and AlphaFold3 modeling to define the molecular role of arginine and tyrosine in *Latrodectus hesperus* dragline silk. Phosphate triggers LLPS while preserving intrinsic disorder, with arginine exhibiting the largest chemical shift perturbations. Simulations reveal that phosphate displaces hydration water to promote Arg–Tyr cation–π interactions and weaken Arg–poly(Ala) contacts. Solid-state NMR directly detects Arg–Tyr contacts in spun fibers, demonstrating that arginine is partially incorporated into β-sheet interfaces while tyrosine frequently adopts β-turn conformations. AlphaFold3 models corroborate these interfacial geometries and reproduce experimental chemical shifts, supporting persistent Arg–Tyr interactions at structured–unstructured boundaries. Together, these results identify Arg–Tyr contacts as critical “sticker” interactions that mediate condensation, nucleate local order, and stabilize fiber architecture. More broadly, this work establishes a mechanistic link between residue-specific chemistry, LLPS, and hierarchical assembly in a structural protein. These insights highlight how weak multivalent interactions bridge disordered and ordered states, providing a general framework for condensate-driven assembly in biology and guiding biomimetic material design.

## Significance

Spider silk is one of nature’s toughest materials, yet how its soluble protein building blocks transform into solid fibers remains elusive. We show that interactions between specific amino acids—arginine and tyrosine—act as molecular “stickers” that connect liquid–liquid phase separation (LLPS) with structural ordering. These interactions promote condensation and persist within emerging β-sheet regions, linking molecular chemistry to macroscopic assembly. By integrating NMR spectroscopy, molecular simulations, and AI-based structural modeling, our work reveals how sequence-encoded interactions guide silk protein organization. These findings establish a framework for understanding how proteins harness phase separation to form functional materials and may inspire design principles for next-generation biomimetic fibers.

Liquid–liquid phase separation (LLPS), also called coacervation, is a dynamic process in which intrinsically disordered proteins (IDPs), normally dilute in the cell environment, concentrate together to form dense droplets sometimes referred to as “membraneless organelles” (1–5). These condensates are important for diverse cellular functions, including stress adaptation and transcription regulation, and form in response to specific stimuli such as pH, molecular crowding, or temperature. LLPS is driven primarily by weak, multivalent interactions often described by the stickers and spacers model, in which stickers, such as Tyr, Gln, or RGG motifs, interact to drive coacervation, while spacers, such as Gly-rich regions, impact the viscosity of the droplets by influencing flexibility of the protein backbone (6–8). Several studies have shown that π–π and cation–π interactions between aromatic and charged amino acids, notably Arg and Tyr, substantially impact droplet formation (9–11).

LLPS has recently gained momentum in biomaterials research, where coacervation enables controlled assembly in a bottom–up, hierarchical manner in natural materials such as squid beaks, insect chitosan, underwater adhesives, nacre, and mantis shrimp clubs (6, 7, 12–14). In spiders and silkworms, phase separation has long been recognized as an important aspect of the silk spinning mechanism (15–19). Silk proteins, known as spidroins in spiders, are initially stored at 30 to 50 wt% (20–22) in an IDP hydrogel which separates into protein-dense and protein-dilute phases in response to elongational flow in the duct (23, 24). Shear forces combined with acidic pH and salt exchange facilitate removal of water and initiate hydrogen bonding between protein chains within the concentrated liquid crystalline phase, resulting in ordered, β-sheet-rich fibers (17, 23, 25). Molecular alignment of the crystalline regions within the fiber is key to achieving desirable mechanical properties. Attempts to mimic this process and spin synthetic fibers from recombinant proteins have so far resulted in silk that is inferior to the native material (26–29). While LLPS has been studied in recombinant silks (30–32), its role in native spidroin processing and sidechain-driven assembly remains unclear.

Recently, multivalent anions such as phosphate, citrate, and sulfate have been shown to induce LLPS in spidroins (33–37). Recombinant studies indicate the effect follows the Hoffmeister series—chaotropic ions prevent premature aggregation by favoring the IDP state while kosmotropic ions induce coacervation by promoting hydrophobic interactions (34, 38–40). However, further investigation found phosphate is more effective than sulfate at inducing fibril formation, indicating phosphate may have a more direct role in facilitating LLPS and subsequent fiber formation (41, 42). Fibers spun from LLPS recombinant spinning dopes have enhanced toughness compared to fibers spun from dopes which do not utilize phosphate-induced coacervation, suggesting that phosphate plays a role in alignment of β-sheets (43). NMR structural analysis on these systems both before and after spinning indicate changes in Tyr ring packing, whereas in all other aspects the protein is nearly identical between the two preparations with similar β-sheet content in the spun fibers (44).

**Fig. 1.**
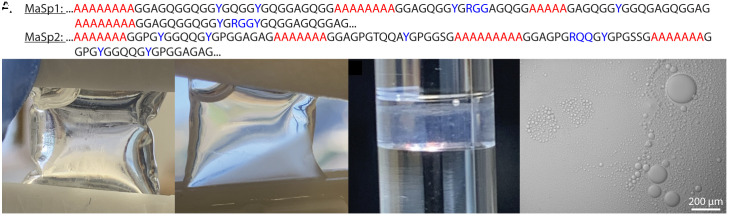
Phosphate induces LLPS in native MaSp. (*A*) Consensus sequence for *L. hesperus* MaSp1 and MaSp2. Poly(Ala) regions (red) form β-sheets in fibers; Tyr- and Arg-rich motifs (blue) are hypothesized to mediate LLPS. (*B*) Solubilized silk in 1 M urea is initially clear. (*C*) Dialysis against 300 mM KH_2_PO_4_ induces turbidity and formation of a condensed protein phase. (*D*) Condensed phase in NMR tube for data collection. (*E*) Brightfield image shows LLPS droplets (10 to 100 μm).

Native MA, or dragline silk, is mainly composed of MA spidroins 1 and 2 (MaSp1 and MaSp2), each approximately 250 to 300 kDa in size (45). Both contain Gly-rich GGX (X = Ala, Gln, Tyr, Arg, Ser) repeats sandwiched between poly(Ala) regions, but MaSp2 is unique in that it contains Pro. Previous compositional analysis based on Pro content indicate that *Latrodectus hesperus* dragline silk contains approximately fourfold to fivefold higher MaSp1 than MaSp2 (46), justifying our focus on MaSp1 for the sequence analysis, relaxation studies, and simulations. Gly, Ala, and Gln are major components, while Tyr, Pro, Ser, and Arg make up a smaller but not irrelevant percentage of the silk, where Tyr is present in all Gly-rich regions and RGG (MaSp1) and RQQ (MaSp2) motifs are present in nearly every other Gly-rich region (Fig. 1*A*). Before spinning, the secondary structure of the repetitive domains is primarily random coil (RC) as determined by NMR (20–22, 47). Spidroins form micelle-like structures with a hydrophobic poly(Ala) core and solvent-exposed hydrophilic residues including Tyr and Arg (48). After spinning, poly(Ala) regions adopt nanocrystalline β-sheet structure, while the Gly-rich regions are thought to be disordered and non-β-sheet, with evidence for 3_10_-helical structures for GGX and elastin-like type-II β-turn structures for the MaSp2 GPGXX motif (46, 49–51). Secondary structure has been studied extensively in MA silk fibers and our lab has recently begun to explore sidechain interactions (44, 52).

Here, we sought to uncover atomic-level interactions and mechanisms that govern the formation of spidroin condensates and their transition to b-sheet-rich fibers. To target specific sidechain interactions, we prepared ^13^C/^15^N-enriched black widow (*L. hesperus*) MA silk by feeding spiders isotopically labeled Arg and Phe while substituting unlabeled amino acids in their diet to promote selective incorporation and minimize isotope scrambling. Phosphate-induced LLPS phases in MA silk were compared to intact glands using solution NMR to investigate secondary structure, dynamics, and through-space interactions. Spectra were acquired at 800 MHz using a triple-resonance TXO cryoprobe, which allows direct detection of ^13^C and ^15^N nuclei. Four sample types were analyzed by solution-state NMR: intact glands, protein solubilized in 1 M urea, the phosphate-induced condensed phase, and the corresponding dilute phase. The condensed and dilute phases represent the protein-rich and protein-poor fractions formed after LLPS. Because of the very low protein concentration in the dilute phase, only ^13^C spectra could be obtained, whereas relaxation and multidimensional experiments were conducted on the condensed phase, intact glands, and urea samples. Pairwise comparisons were carried out primarily between the condensed phase and intact glands to evaluate phosphate-induced changes in secondary structure and dynamics, with 1 M urea serving as a denatured control. The dilute phase was included only for qualitative comparison of ^13^C chemical shifts where sufficient signal was obtained. This X-detect approach improves resolution for IDPs by avoiding polarization transfer to ^1^H, which is often inefficient in environments with μs–ms dynamics (53). To evaluate whether Arg–Tyr interactions persist in the assembled fiber, we used Dynamic Nuclear Polarization–Magic Angle Spinning Solid-State NMR (DNP-MAS SSNMR) to detect direct evidence of cation–π contacts in solidified fibers. NMR data were supported by computational models generated using both ColabFold (54) and AlphaFold3 (55). These models were validated by predicting chemical shifts with SHIFTX2 (56) and secondary structure with DSSP (57, 58), and comparing to experimental values. Our results provide atomic- and molecular-level insight into LLPS and fiber assembly in MA spider silk, revealing strong evidence for Arg–Tyr-mediated interactions in both condensed phases and fiber states. Notably, Arg appears partially incorporated at the interface of β-sheet domains, where it participates in stabilizing cation–π interactions with Tyr residues.

## Results

### Phosphate Induces LLPS in Native Black Widow MA Silk.

Although phosphate-induced LLPS has been shown in small recombinant spider silk proteins, it has only been demonstrated to a minimal extent in native systems (33). Here, we show that the protein-rich spinning dope extracted from black widow MA glands undergoes LLPS when exposed to potassium phosphate (Fig. 1). First, we solubilized the extracted silk dope in urea to form a homogenous solution (Fig. 1*B*). We then dialyzed the solution against 300 mM potassium phosphate in 1 M urea at pH 7.2, resulting in a cloudy, turbid phase and a clear protein-dilute phase (Fig. 1*C*). The turbid phase indicates the formation of micron-scale protein-rich droplets characteristic of LLPS. When stored in a Shigemi NMR tube overnight at room temperature, the turbid phase settled into a condensed phase (Fig. 1*D*) while the dilute phase was removed and transferred to a separate NMR tube for data collection. Viewed with light microscopy, the turbid phase contained droplets ranging from 10 to 100 μm in diameter (Fig. 1*E*), which coalesced to form the condensed phase overnight.

### Intrinsic Disorder Is Retained after LLPS.

Solution NMR has proven to be a powerful technique for probing LLPS in a number of systems including Fused in Sarcoma (FUS) (59–61), DEAD-box helicase 4 (DDX4) (62, 63), elastin-like polypeptides (64), and in one case a short MA spidroin repetitive domain (38). Based on prior LLPS and mutagenesis studies in other systems (1, 8, 65–67) including recombinant spidroins (68) which implicate Arg–Tyr interactions in droplet formation, we selectively labeled these residues in native MaSp for NMR analysis. Secondary structure of individual amino acids was assigned by analyzing chemical shifts, where specific trends in Cα, Cβ, carbonyl, and amide chemical shift differences indicate β-sheet, α-helix, and RC structure (69, 70). Prior to analyzing chemical shifts, peaks were assigned using a combination of CACO/CAN (*SI Appendix*, Fig. S1), INADEQUATE (*SI Appendix,* Fig. S2), and HNCACB/CBCA(CO)NH (*SI Appendix,* Fig. S3) spectra.

**Fig. 2.**
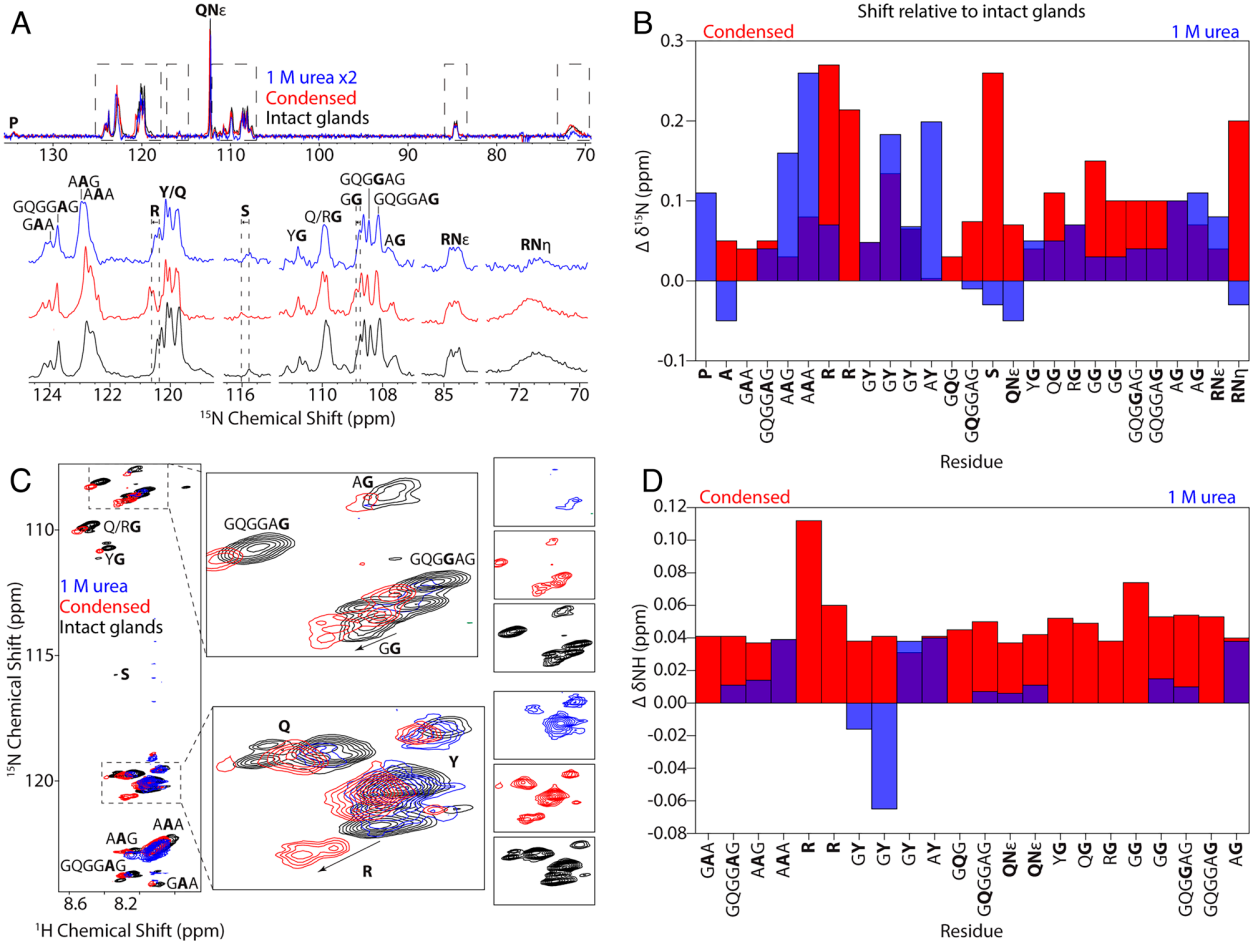
Intrinsic disorder is maintained following phosphate-induced LLPS and urea denaturation. (*A*) Stacked ^15^N direct-detect spectra of MaSp in intact glands (black), condensed phase (red), and 1 M urea (blue). Dashed lines highlight largest chemical shift perturbations. (*B*) ^15^N chemical shift differences in condensed (red) and 1 M urea (blue) phases relative to the intact glands. (*C*) Superimposed ^1^H-^15^N HSQC spectra under all three conditions. Arrows indicate residues with large chemical shift differences. The individual spectra (not overlapped) are shown to the right for clarity. (*D*) Amide proton chemical shift differences in condensed phase (red) and 1 M urea (blue) relative to intact glands. Chemical shift perturbations greater than 0.03 ppm for ^1^H and 0.1 ppm for ^15^N are considered significant consistent with established thresholds for protein NMR analysis.

To understand how LLPS affects the secondary structure of MaSp, ^15^N direct-detect and ^1^H-^15^N HSQC spectra were collected for intact glands, MaSp solubilized in 1 M urea, and MaSp in the condensed phase after dialysis against 300 mM phosphate (Fig. 2). In the ^15^N HSQC experiment (Fig. 2*C*), two unique shifts were observed for Arg in the condensed phase, whereas only one Arg shift could be resolved from the Tyr peaks in the gland and urea samples. The largest ^15^N chemical shift perturbations for the condensed phase were observed for Arg (0.27 ppm, 0.21 ppm), Ser (0.26 ppm), a GG motif (0.15 ppm), and the guanidino groups of the Arg side chain (0.2 ppm). The Arg side chain also exhibited some degree of line sharpening in the condensed phase indicative of structural ordering. In 1 M urea, the largest shifts were observed for A**A**G (0.16 ppm), A**A**A (0.26 ppm), G**Y** (0.18 ppm), and A**Y** (0.20 ppm). While phosphate is a kosmotropic ion, urea is a chaotropic denaturant, which could account for the opposing shifts in some cases. Interestingly, while phosphate affects the hydrophilic Gly-rich region and Arg sidechains, urea primarily affects the hydrophobic poly(Ala) and flanking A**A**G regions of the protein, consistent with previous NMR chemical shift measurements for native black widow MaSp solubilized in 4 M urea (71).

Similar trends were observed in the ^1^H-^15^N HSQC spectra (Fig. 2 *C* and *D*); in the condensed phase, the largest chemical shifts for amide protons were observed in Arg (0.11 ppm, 0.06 ppm) and one of the G**G** motifs (0.07 ppm), whereas in 1 M urea, the largest shifts were observed in poly(Ala) residues (0.04 ppm), G**Y** (−0.07), A**Y** (0.04 ppm), and the A**G** motif (0.04 ppm), which is commonly found in the Gly-rich domains that terminate the poly(Ala) runs (71). Arg, the G**A**A motif, and several of the Gly residues were not observed in the ^1^H–^15^N HSQC of the 1 M urea sample but remained visible in the ^15^N direct-detect spectrum, consistent with exchange broadening under denaturing conditions.

While recombinant studies have indicated spider silk adopts β-sheet secondary structure for the terminal domains within LLPS droplets based on circular dichroism (CD) measurements (34), the small chemical shift perturbations seen across all three samples in the present study indicate that native silk maintains intrinsic disorder in the repetitive core in LLPS samples, and condensate formation does not induce the β-sheet structure known to form between poly(Ala) regions in the spun fiber to any appreciable extent (*SI Appendix,* Tables S1 and S2).

^13^C and ^1^H chemical shifts measured in ^13^C direct-detect and ^1^H-^13^C HSQC spectra further support that a primarily RC structure is maintained in the condensed phase (*SI Appendix,* Fig. S4 and Tables S3 and S4). Arg, Pro, Tyr, and Ser showed the largest shifts in the condensed phase (0.10 to 0.15 ppm), whereas Ala showed the largest perturbations in urea (−0.14 ppm), consistent with phosphate impacting the hydrophilic Gly-rich regions and urea affecting the hydrophobic poly(Ala) domains. The dilute phase, representing the protein fraction that remains soluble after phase separation, was analyzed as a control to distinguish chemical shift changes specific to condensation from those intrinsic to the dilute protein. The most pronounced differences between the dilute and condensed phases were observed in Arg and Tyr residues, including sidechain sites, suggesting that LLPS alters residue–residue sidechain contacts without inducing large-scale changes in backbone conformational structure (*SI Appendix,* Fig. S4). Analysis of Cα, Cβ, and carbonyl (CO) chemical shifts (*SI Appendix*, Table S3) confirms that deviations remain below standard chemical-shift index (CSI) thresholds for β-sheet or α-helix formation, while correlated ^1^Hα and ^1^Hβ values (*SI Appendix*, Table S4) show similar trends consistent with disordered backbone structure.

### Phosphate Modulates Gly-Rich Backbone Dynamics in the Condensed Phase.

**Fig. 3.**
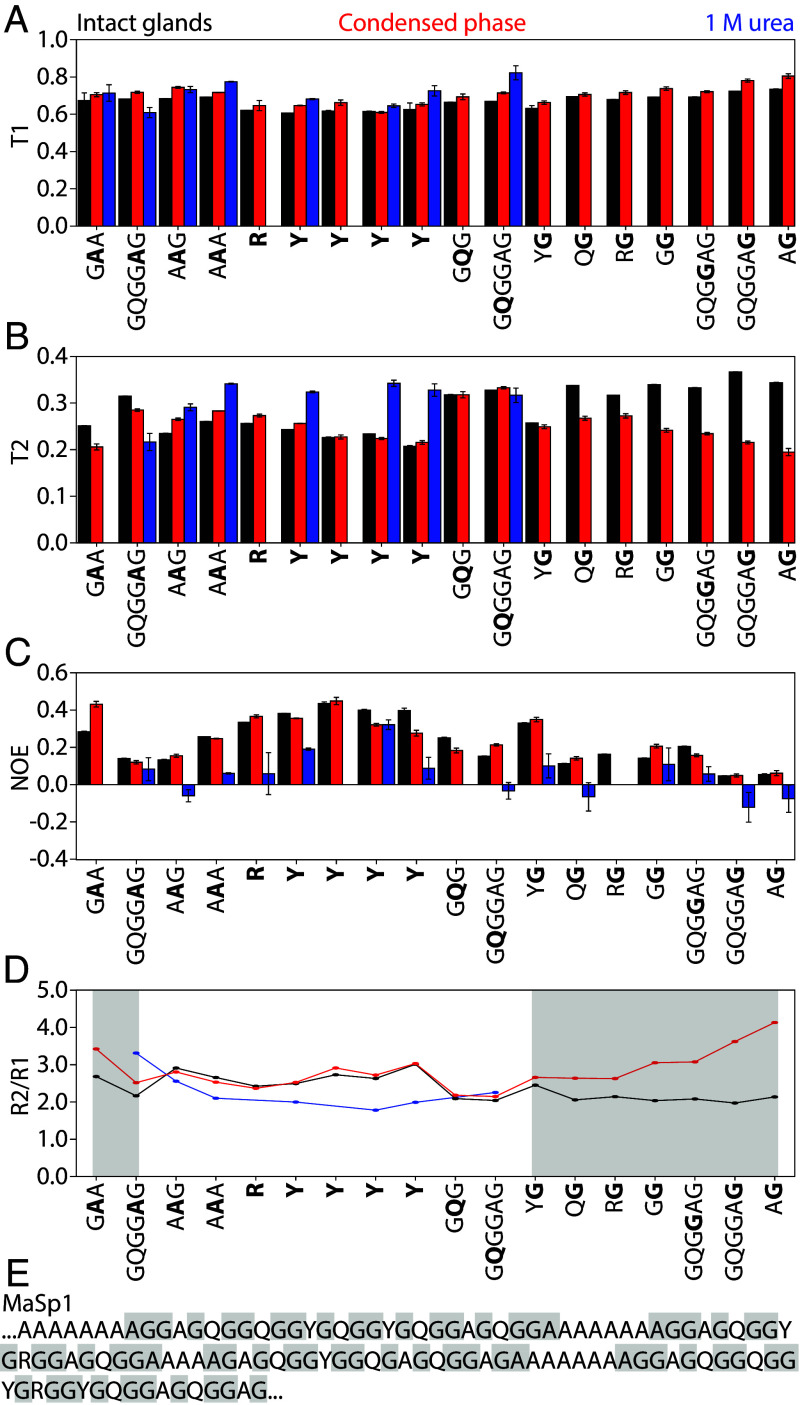
Backbone dynamics are modulated by phosphate in Gly-rich regions. (*A*–*C*) T_1_, T_2_, and NOE values measured in intact glands (black), condensed phase (red), and 1 M urea (blue). (*D*) R_2_/R_1_ ratios calculated from T_1_ and T_2_ highlight changes in dynamics, regions of interest shaded in gray. (*E*) Annotated MaSp1 sequence with regions of interest from (*D*) shaded in gray. T_2_ shortening may reflect either local rigidification or increased conformational exchange on the μs–ms timescale.

To determine how phosphate affects backbone dynamics, we compared ^1^H-^15^N relaxation parameters measured for intact gland, condensed phase, and 1 M urea samples (Fig. 3). ^15^N T_1_ and T_2_ relaxation times and ^1^H-^15^N NOE provide insight into both global and local backbone dynamics for residue sites, and have been measured previously for abundant residues in intact black widow MA glands (47, 72). The average T_1_, which reports on high frequency motions (10^8^–10^12^ s^−1^), was similar for all three samples: 0.67 ± 0.04 s for intact glands, 0.70 ± 0.05 s for the condensed phase, and 0.71 ± 0.07 s for the 1 M urea sample (Fig. 3*A* and *SI Appendix,* Fig. S5 and Table S5). In urea, signal in the Gly-rich region was too weak to measure T_1_, presumably due to rapid amide proton exchange. Similar T_1_ values indicate conserved high frequency dynamics across sample conditions. This is consistent with the chemical shift analysis discussed above, where intrinsic disorder is maintained across the phases.

T_2_ relaxation measurements, which are sensitive to both fast (ps-ns) and intermediate (μs–ms) timescale motions, revealed phase-dependent differences in backbone dynamics (Fig. 3*B* and *SI Appendix,* Fig. S5 and Table S6). The condensed phase showed the shortest average T_2_ (0.25 ± 0.04 s), followed by intact glands (0.29 ± 0.05 s), while 1 M urea displayed the longest T_2_ (0.31 ± 0.04 s), consistent with the highest backbone mobility for the urea condition. Interestingly, while Arg, Tyr, and Gln residues had similar T_2_ values in intact and condensed phases, Gly residues—particularly in R**G**, G**G**, **G**AG, GA**G**, A**G** motifs—showed enhanced T_2_ relaxation in the condensed phase. This T_2_ shortening may reflect either local rigidification or, more likely, increased conformational exchange on the μs–ms timescale driven by transient interactions. These trends are reinforced by elevated R_2_/R_1_ ratios (Fig. 3*D*), especially in Gly-rich segments, indicating that LLPS alters the dynamic behavior of these domains. T_2_ values could not be reliably measured for the Gly-rich regions in 1 M urea due to low signal, but previously published results in 4 M urea reported similar T_2_ values across residues, consistent with a fully disordered and flexible protein (71).

NOE measurements varied by residue and phase (Fig. 3*C* and *SI Appendix,* Table S7). Average NOE values were 0.23 ± 0.12 for intact glands, 0.24 ± 0.12 for the condensed phase, and 0.05 ± 0.12 for the 1 M urea sample, with the latter indicating greater ps–ns dynamics in the urea sample relative to the other two. Arg and Tyr residues exhibited higher NOEs (~0.3 to 0.4) than most Gly residues (~0.1 to 0.2), except for Y**G** motifs, suggesting greater rigidity and supporting T_2_ relaxation data. Gln displayed intermediate NOEs (~0.2), indicating more flexible dynamics similar to Ala and Gly. Notably, the Ala residue beginning poly(Ala) blocks had higher NOEs in the condensed phase than in intact glands, suggesting decreased flexibility in G**A**A motifs. This aligns with T_2_ data and points to localized ordering near poly(Ala) termini, which may act as nucleation zones for β-sheet formation. These dynamic differences across Gly-rich regions—with Q-containing segments remaining flexible while R- and Y-containing regions exhibiting higher rigidity—highlight the dynamic heterogeneity within the repetitive domain (Fig. 3*E*).

### Spectral Density Mapping Reveals Localized Motional Restriction.

Spectral density mapping (*SI Appendix*, Table S21) highlights distinct patterns of backbone motion across the three sample states. Residues within G**A**A, A**G**, GQGGA**G,** and G**G** motifs exhibit the highest J(0) values and J(0)/J(ωH) ratios in the condensed sample relative to the intact gland and urea-solubilized sample, indicating restricted local motions on the ns-to-µs timescale. These increases are localized to specific Gly-rich repeat elements, consistent with partial ordering or intermolecular association occurring selectively at these motifs during LLPS or early assembly. By contrast, residues within the A**A**A and A**A**G segments show comparable J(ωH) values between the intact and condensed samples (though higher in the urea condition), suggesting that fast ps–ns backbone motions are largely preserved upon condensation. The combination of enhanced low-frequency spectral density and maintained high-frequency flexibility supports a partially ordered, dynamically restricted ensemble rather than complete rigidification. Together, these results indicate that MaSp1 retains overall flexibility while selectively suppressing backbone mobility at Gly–Ala and Gly-rich interfaces that nucleate intermolecular interactions during condensation. Because the relaxation parameters reflect ensemble-averaged behavior, residues with intermediate spectral-density values may represent averages over multiple dynamic subpopulations within the disordered ensemble.

^13^C NOESY-HSQC spectra were collected on both intact glands and the condensed phase (*SI Appendix,* Fig. S6). Similar contacts were observed in both samples except for amide resonances, which were only observed in the intact glands consistent with an increase in exchange processes in the condensed phase as noted in the T_2_ discussion. Overlapping Gln, Arg, and Tyr side chain resonances were weak and difficult to resolve and did not allow observation of Arg–Tyr contacts indicative of cation- π interactions.

### Phosphate enhances Arg–Tyr interactions and disrupts Arg–Ala contacts.

**Fig. 4.**
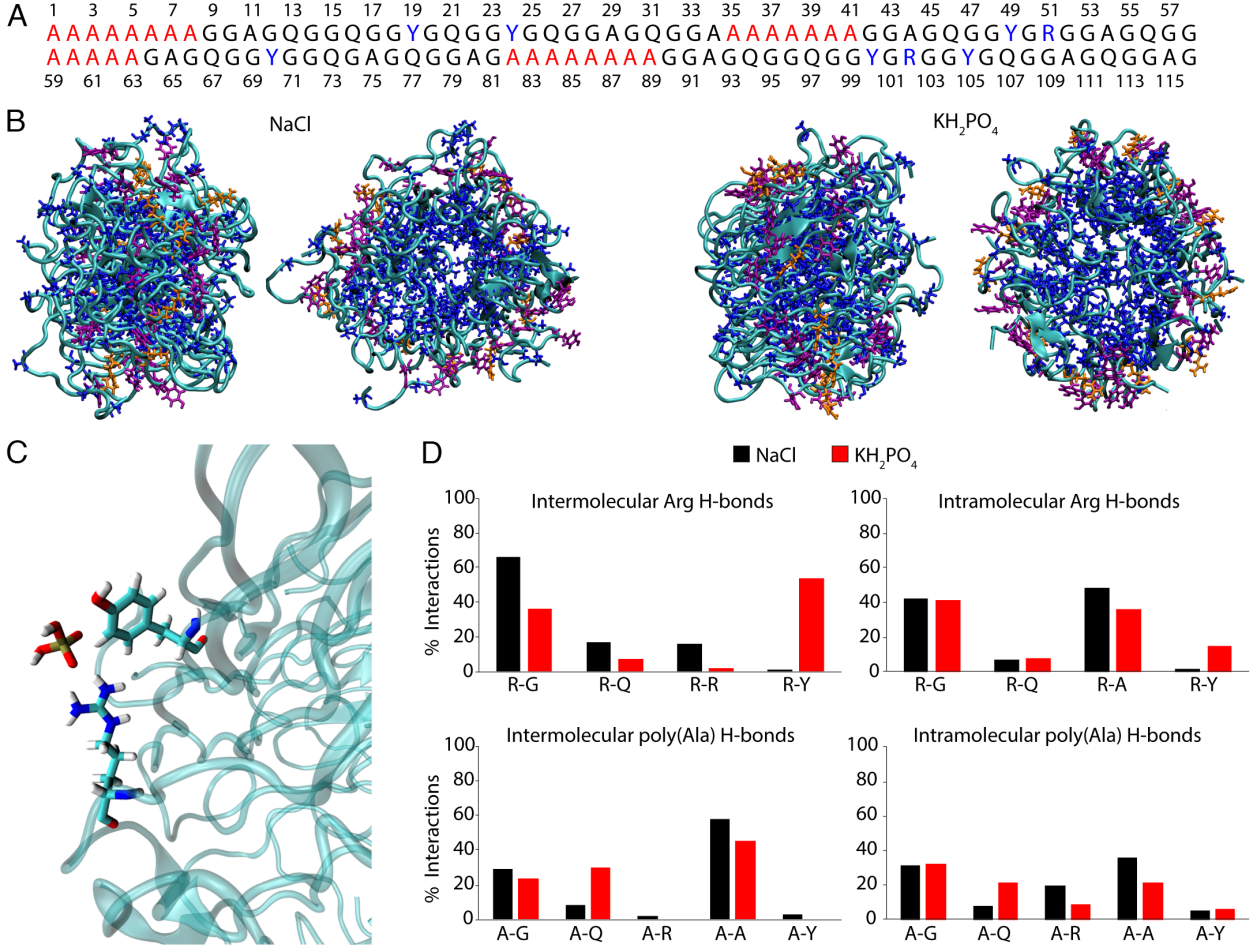
Molecular dynamics simulations reveal phosphate promotes Arg–Tyr interactions and disrupts Arg–Ala contacts. (*A*) 116-residue MaSp1 fragment used in simulations, with poly(Ala) (red) and Arg, Tyr (blue) highlighted. (*B*) Representative structures from simulations run with NaCl (*Left*) and KH_2_PO_4_ (*Right*). (*C*) Example of a phosphate-bridged Arg–Tyr interaction observed in KH_2_PO_4_. (*D*) Frequency of intermolecular (*Left*) and intramolecular (*Right*) hydrogen bonds involving Arg (*Top*) or poly(Ala) (*Bottom*), comparing NaCl (black) and KH_2_PO_4_ (red) conditions.

All-atom MD simulations were performed on six copies of a 116-residue fragment from the MaSp1 sequence (Fig. 4*A*) under three solvent conditions matching the experimental NMR samples: NaCl (intact gland), KH_2_PO_4_ (condensed LLPS phase), and urea (denatured) (Fig. 4*B* and *SI Appendix,* Figs. S7 and S8). The assemblies mimic the micelle-like organization previously observed in native MaSp by Cryo-EM featuring a solvent-filled core (48). DSSP analysis (*SI Appendix,* Table S8) confirmed that all conditions maintained a predominantly disordered backbone conformation, with loops and bends accounting for over 65% of the structure. A modest increase in β-strand content in the KH_2_PO_4_ condition suggests early β-structure formation during LLPS, though appreciable secondary structural transitions were not observed.

To validate the simulated micelle-like assemblies, chemical shifts were predicted using SHIFTX2 and compared to experimental values (*SI Appendix,* Tables S9–S12). Across the ensemble, predicted ^13^C shifts showed low variability (SD ≤ 1 ppm), while ^15^N shifts exhibited greater dispersion—particularly for Arg and Tyr sidechains, where some sites varied by more than 2.5 ppm. Despite this heterogeneity, the average predicted shifts for ^13^C agreed closely with experimental data (typically within ≤1 ppm), and predicted amide NH chemical shifts matched experimental values within ≤0.2 ppm. While some ^13^C chemical shifts—particularly for Ala, Tyr, and Arg—trend slightly toward values characteristic of β-sheet structure, the majority remain closer to disordered or RC regions. This is consistent with DSSP analysis (*SI Appendix,* Table S8), which classifies most residues as disordered with little persistent secondary structure. Together, the strong cross-nucleus agreement and partial shift trends support the relevance of the simulated assemblies while also reinforcing that they represent a compact but largely disordered micelle-like state, distinct from the β-sheet–rich silk fiber. The structural relevance of the modeled assemblies supports their use in analyzing how solvent conditions and ion identity modulate sidechain interactions in native MaSp1.

Phosphate ions displayed considerably stronger and more frequent binding to the peptide assemblies than chloride, interacting ~71% of the time versus ~10% for Cl^−^. In Arg residues, phosphate binding displaced an average of three water molecules from the first hydration shell and promoted Arg–Tyr interactions within (intramolecular) and between (intermolecular) chains. When phosphate was bound, intramolecular Arg–Tyr hydrogen bonds formed ~24% of the time, compared to ~10% when unbound (Fig. 4*C*). In contrast, chloride binding reduced Arg–Tyr hydrogen bonding (from 16% to 10%), supporting a phosphate-specific stabilization of Arg–Tyr cation–π interactions.

Phosphate also altered the broader hydrogen bonding landscape. Assemblies in KH_2_PO_4_ showed fewer total intermolecular hydrogen bonds compared to NaCl, consistent with a more dynamic and disordered condensed phase. However, among remaining interactions, Arg–Tyr contacts were the most frequent in phosphate (intermolecular 54%, intramolecular 15%), while Arg–Gly, Arg–Gln and Arg–Arg dominated in NaCl (Fig. 4*D*). Phosphate further disrupted Arg–Ala hydrogen bonding observed under NaCl conditions, weakening interactions with poly(Ala) domains. Together, these results suggest that phosphate promotes LLPS by displacing hydration water and shifting the sidechain interaction network toward Arg–Tyr contacts, consistent with a cation–π driven mechanism for condensed phase stabilization.

### Tyr–Arg Cation–π Interactions Persist in the Fiber State, with Arg Located at β-Sheet Interfaces.

Solution NMR and MD simulations indicated that Arg–Tyr interactions are enhanced in phosphate-induced condensates, raising the possibility that these interactions contribute to the structural transition toward fiber formation. To investigate their role in the solid state, we conducted DNP-enhanced MAS SSNMR experiments on isotope-enriched black widow *L. hesperus* MA silk fibers. DNP, which combines low-temperature MAS (~100 K), microwave irradiation, and exogenous radicals, greatly enhances NMR sensitivity and allows detection of otherwise invisible interactions (73–75). This substantial enhancement can be used to reveal interactions that are not visible with standard MAS SSNMR. DNP has previously been applied to dragline spider silk—achieving up to 64-fold enhancement in ^13^C CP-MAS spectra collected at 400 MHz (76).

Here, we performed DNP experiments on fibers labeled with U-[^13^C/^15^N]-enriched Phe, Ala, and Arg which labels Ala, Gly, Gln, Tyr, Pro, and Arg. 10 mM AMPUol in a 80:20 (v/v) ratio of D_2_O/H_2_O was used as the radical/solvent combination. EPR spectroscopy experiments found a final AMUPol concentration of 19 mM in the rotor (*SI Appendix,* Fig. S18). DNP enhancements (ε_DNP_) were on the order of 22 to 35 at 600 MHz (*SI Appendix,* Fig. S9), values that are modestly lower than previous reports but consistent with expectations at higher magnetic fields (76, 77). Importantly, these enhancements enabled acquisition of sidechain-specific spectra for Arg, a residue that is rarely characterized in SSNMR due to low signal and overlapping resonances. Combining partial Arg labeling with Tyr aromatic enrichment allowed us to probe Arg–Tyr sidechain interactions in detail via 2D ^15^N-^13^C correlation experiments.

**Fig. 5.**
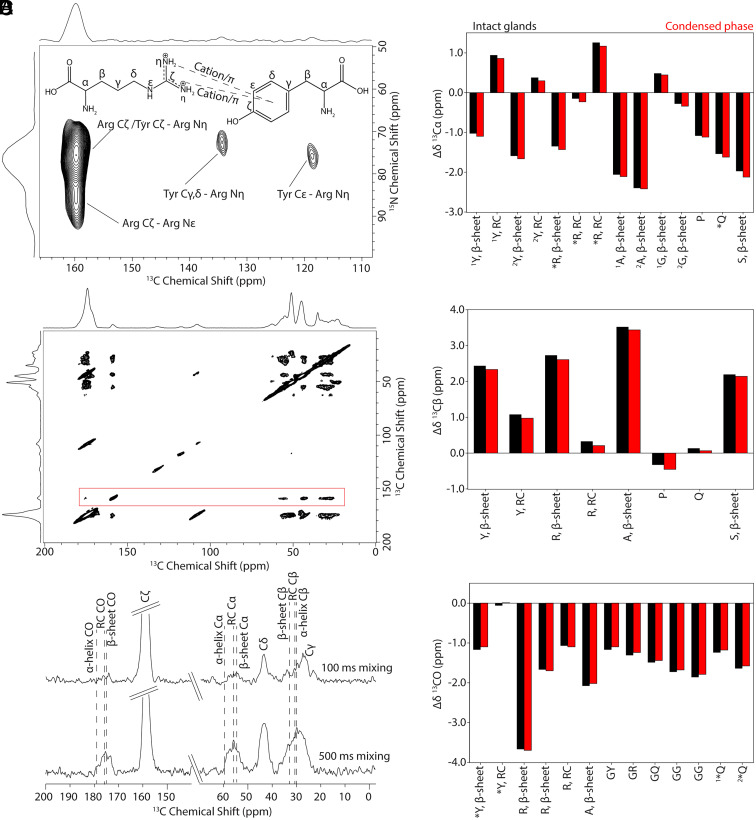
Solid-state NMR reveals Arg–Tyr cation–π interactions and β-sheet incorporation of Arg in spider silk fibers. (*A*) 2D DNP-enhanced MAS ^15^N–^13^C hNHHC spectrum of *L. hesperus* major ampullate silk fiber labeled with U-^13^C/^15^N-Phe, -Ala, and -Arg, acquired at 98.2 K, 15 kHz MAS, and 600 MHz, with a mixing time of 500 μs. Cross-peaks between Arg side chain nitrogens and Tyr aromatic carbons indicate cation–π interactions within the fiber. (*B*) ^13^C–^13^C DARR spectrum of silk selectively labeled with U-^13^C/^15^N-Arg, recorded with a 500 ms mixing time at room temperature, 10 kHz MAS, and 600 MHz. The boxed region (red) shows a slice through the Arg Cζ signal at 159.0 ppm, highlighting Arg-specific contacts. (*C*) The corresponding 1D slices taken at Arg Cζ from DARR spectra at 100 and 500 ms mixing times demonstrate structural heterogeneity, with Arg exhibiting both β-sheet and RC conformations. Common secondary structure chemical shift positions are indicated with dashed lines. (*D*) Changes in ^13^C chemical shifts (Δδ) between fiber and intact gland (black) and condensed (red) states reveal disorder-to-order transitions, with most residues showing substantial β-sheet signatures. *Arg and Gln Cα peaks and Tyr and Gln CO peaks could not be fully resolved in solution NMR. Two sets of Cα shift changes are shown for Ala, Gly, and Tyr and two sets of CO shift changes are shown for Gln (labeled “1” and “2”) based on distinct environments observed in solution NMR.

We first collected a 2D hNHHC spectrum (Fig. 5*A*) which reveals cross-peaks between Tyr Cγ,δ and Cε sites and Arg Nη groups, consistent with cation–π contacts. A potential additional correlation between Tyr Cζ and Arg Nη appears to overlap with the Arg Cζ–Nη signal. The two distinct Arg Nη resonances at 72.5 and 75.8 ppm correlate differentially with the Tyr ring, suggesting asymmetric T-shaped engagement—one guanidinium Nη interacting with the ring edge and the other with the π-face. This pattern supports a single Arg–Tyr cation–π geometry rather than two separate binding modes, aligning with prior reports that T-shaped interactions yield distinct electronic environments for the two Nη sites (78, 79). A stacked geometry would likely produce symmetric environments and less chemical shift dispersion.

Further 2D ^15^N-^13^C Double Cross-Polarization (DCP) (*SI Appendix,* Fig. S10) and ^13^C-^15^N TEDOR (*SI Appendix,* Fig. S11) spectra provided resolved ^13^C and ^15^N chemical shifts for Tyr, Arg, and other labeled residues (*SI Appendix,* Tables S13–S15). A comparison of ^13^C solid-state NMR chemical shifts at room temperature and at DNP temperatures indicates that there is not a significant difference in chemical shift between the two methods outside of Gln, which requires further investigation due to its broad resonances (*SI Appendix,* Table S13). Secondary structure assignments based on literature chemical shifts (80, 81) indicates that most residues are shifted toward β-sheet structure consistent with previous assignments although many resonances are multicomponent including disordered RC environments (44, 82). Interestingly, the ^15^N chemical shifts of Gly, Ala, Tyr, and Arg residues all fall squarely in the β-sheet range, confirming at least partial structural incorporation in ordered domains (83). Notably, Pro from MaSp2 exhibits a ^15^N shift (~133.6 ppm) typical of β-turns, consistent with its role in loop or turn regions (52, 84–86).

Comparison of ^13^C chemical shifts in fibers versus intact glands highlights structural transitions upon fiber formation (Fig. 5*D* and *SI Appendix,* Table S14). Most residues exhibit upfield shifts in Cα and carbonyl positions and downfield shifts in Cβ sites, consistent with RC to β-sheet conversion. While Arg backbone signals are broader, Arg-labeled samples show mixed populations of β-sheet and disordered conformations (Fig. 5 *B* and *C* and *SI Appendix,* Figs. S12–S14). Classic β-sheet chemical shift changes are evident in Arg when comparing fiber to gland and condensed solution phases, suggesting Arg becomes partially integrated into poly(Ala) β-sheet structures (Fig. 5*D*).

Tyr and Gln do not display typical coil-to-sheet transitions exhibiting broad resonances with central chemical shift positions in line with RC. Tyr Cα chemical shifts are unchanged from gland to fiber, while downfield shifts in Tyr Cβ and carbonyl resonances suggest some altered packing of sidechain environments. Close inspection of Tyr shows that the resonances, although centrally positioned at RC, do indeed contain some β-sheet component (*SI Appendix,* Figs. S15 and S16). Gln exhibits overlapping Cβ shifts with solution phase samples but upfield Cα and carbonyl shifts, indicating partial backbone ordering without full β-sheet incorporation. Together, these patterns suggest both residues remain structurally heterogeneous, only partially occupying β-sheet regions and to a lesser degree compared to Ala, Gly, and Arg.

Finally, comparison of ^15^N chemical shifts across solution and fiber states (*SI Appendix,* Table S15) confirms the partial β-sheet transition and involvement of Arg sidechain interactions in assembly. Most residues shift slightly downfield in the condensed phase, but Arg Nη shows the largest deshielding (4.2 and 1.2 ppm) between solution and fiber—a strong perturbation consistent with stabilized cation–π interactions. These data, together with the 2D correlation spectra, support a model in which Arg–Tyr interactions persist through LLPS and are structurally incorporated into the final fiber assembly, potentially playing a critical role in β-sheet formation.

### AlphaFold3 Structures Predict β-Sheet Assembly and Arg–Tyr Interactions in the Fiber.

**Fig. 6.**
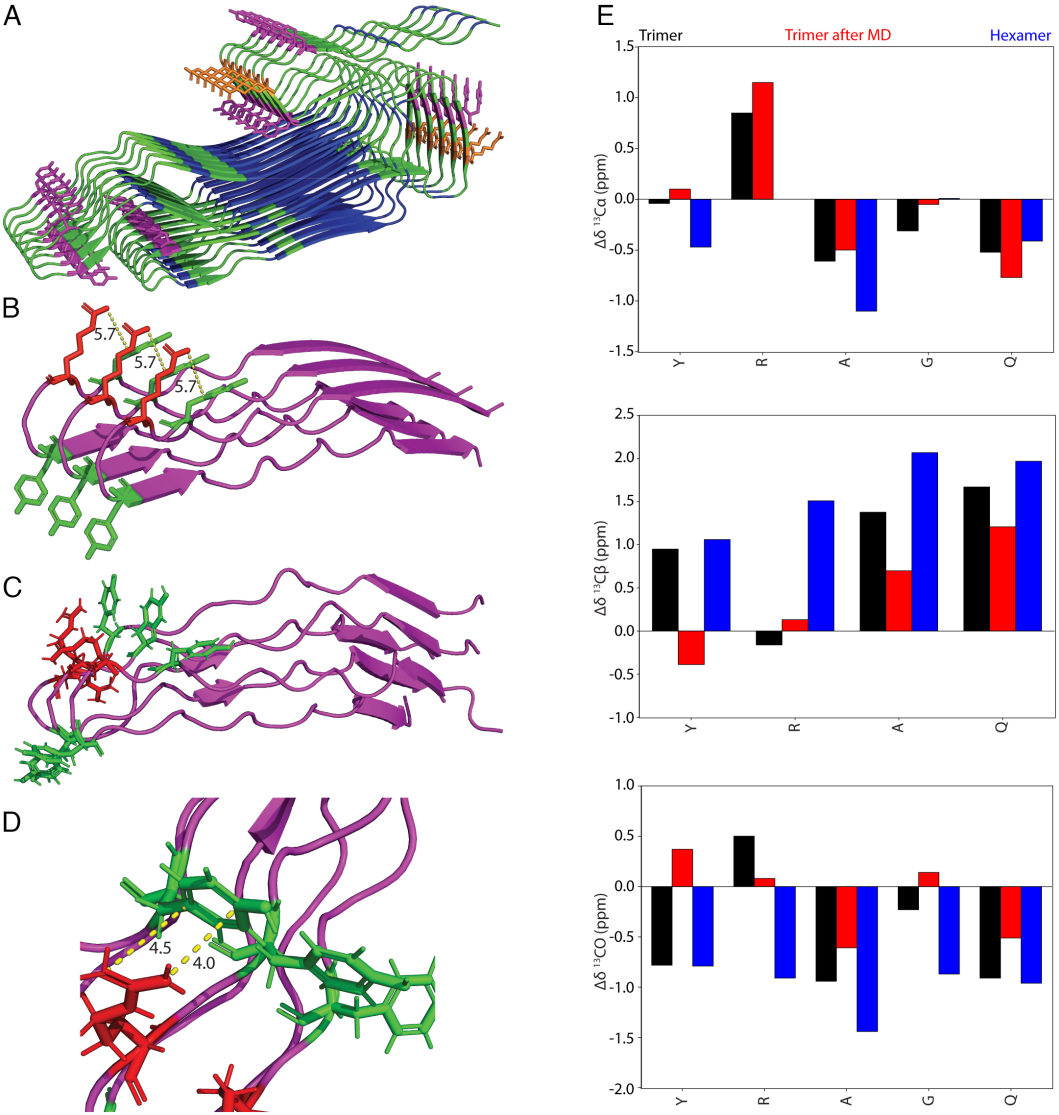
AlphaFold3 models reveal β-sheet fiber architecture with Arg–Tyr cation–π interactions at structured–unstructured interfaces. (*A*) Hexamer model of a 116-residue MaSp1 repeat predicts highly ordered β-sheet architecture, with poly(Ala) regions forming the β-sheet core (blue), and Arg (orange) and Tyr (purple) side chains positioned near β-sheet interfaces. (*B*) Trimer model of a 43-residue sequence containing Arg–Tyr motifs exhibit short β-sheets in poly(Ala) regions (purple). Arg (red) and Tyr (green) residues are located at β-sheet interfaces, with Arg side chains positioned near Tyr aromatic rings. The average distance between the Tyr Y19 ring (center-of-mass) and Arg R21 Nη atoms is ~5.7 Å. (*C*) All-atom CHARMM36m MD simulation (200 ns) preserves poly(Ala) β-sheet structure in the trimer assembly, but reveals noteworthy sidechain disorder in interfacial Arg and Tyr residues. (*D*) Zoomed view of the Arg–Tyr interface from (*C*), highlighting T-shaped cation–π interactions. The two Arg Nη atoms contact the Tyr ring at distances of 4.0 Å and 4.5 Å. (*E*) Predicted ^13^C chemical shift changes (Δδ) between ordered β-sheet AF3 models and the disordered micelle-phase ColabFold/MD model (Fig. 4*B*) reveal disorder-to-order transitions. Most residues show β-sheet shift signatures in the AF3 fiber models, with the hexamer being the most pronounced. Δδ values are shown in black, red, and blue for the trimer, MD-refined trimer, and hexamer, respectively.

While ColabFold models capture the micelle-like, intrinsically disordered conformation of spidroins in solution (Fig. 4), AlphaFold3 (AF3) models more closely resemble the nanocrystalline β-sheet architecture characteristic of the silk fiber. Two AF3 models were generated: a 116-residue hexamer based on the same sequence used in ColabFold (Fig. 6*A*, pTM = 0.33), and a shorter 43-residue trimer, capped with poly(Ala) motifs to promote β-sheet formation (Fig. 6*B*, pTM = 0.67). The shorter model exhibits reduced β-sheet propensity and increased disorder, consistent with the sequence composition (rich in GGX motifs), while still retaining some poly(Ala) β-sheet character. This model reflects the intermediate zones bridging highly ordered poly(Ala) blocks and more flexible, disordered domains. Although the overall confidence scores of the AF3 models are relatively low, particularly for the hexamer, the poly(Ala) motifs are predicted with higher reliability (average pLDDT for Ala–Ala pairs: 42.6 in the hexamer and 75.8 in the trimer). These motifs correspond to experimentally well-characterized β-sheet regions, whereas the surrounding disordered segments remain less certain. Nevertheless, even the low-confidence regions are informative to examine in the AF3 models, as they may offer plausible arrangements of side-chain interactions observed by NMR and serve as a starting framework for future structural refinement

Secondary structure analysis using DSSP shows that in the larger hexamer, 87% of Ala and 43% of Gly residues adopt β-sheet conformations (*SI Appendix,* Table S16), in excellent agreement with prior SSNMR-derived estimates for *L. hesperus* MA silk (83). SHIFTX2-predicted ^13^C and ^15^N chemical shifts for Ala, Gly, and Arg residues in β-sheet regions also show strong concordance with experimental values, validating the model’s structural relevance (*SI Appendix,* Tables S17 and S18). In particular, Arg is positioned at β-sheet interfaces, mirroring one component from the experimental observations described earlier (Fig. 5*C*). Arg Cα, Cβ, and CO sites show distinct populations experimentally: 54.6 ppm (β-sheet), 55.8, and 57.2 ppm (RC); 33.3 ppm (β-sheet) and 30.9 ppm (RC); and 173.4, 175.4 (β-sheet), and 176.0 ppm (RC), respectively (*SI Appendix,* Table S14). SHIFTX2-predicted values for these Arg sites (Cα: 55.0 ppm, Cβ:33.6 ppm, CO: 175.0 ppm) in the hexamer align well with the β-sheet populations, supporting the presence of Arg in structured domains.

To assess unstructured regions, we generated a second AF3 trimer model which predicted a lower β-sheet content, preserving some β-sheet character in poly(Ala) segments while Gly-rich domains were more disordered (*SI Appendix,* Table S19). This model better reflects the heterogenous regions adjacent to structured poly(Ala) blocks and enables closer examination of Arg–Tyr contacts. In the 43-residue trimer (Fig. 6*B*), the distance between Tyr (Y19) and Arg (R21) side chains averages 5.7 Å—a range consistent with cation– π interactions that are expected to be 4-6 Å and observable by the SSNMR ^15^N–^13^C hNHHC experiment (Fig. 5*A*) (87, 88). The guanidinium group approaches the Tyr ring side-on, forming a T-shaped geometry supported by asymmetric ^15^N–^13^C correlations observed in the DNP SSNMR data (Fig. 5*A*). These structural predictions substantiate the presence of stable Arg–Tyr interactions that persist during fiber assembly both experimentally and through AF3 models.

To further validate the cation–π contact and its potential in RC Arg domains, we performed a 200 ns all-atom MD simulation starting from the trimer AF3 model. The poly(Ala) β-sheet character was mostly retained (*SI Appendix,* Table S20), while Arg side chains exhibited enhanced disorder—consistent with the SSNMR-derived secondary shift analysis where a broad distribution of Arg components was observed including RC components (Fig. 5*C*). Critically, strong Arg–Tyr contacts persisted throughout the simulation, with Arg Nη atoms engaging the Tyr ring at distances of 4.0 and 4.5 Å (Fig. 6 *C* and *D*), within the expected range for cation–π stabilization and even tighter packing than the initial trimer AF3 model (Fig. 6*B*).

The smaller trimer model also refines our understanding of Tyr structure. While DSSP assigns Tyr residues as β-sheet (*SI Appendix,* Table S19), close inspection reveals that Tyr often resides at β-sheet edges or adopts β-turn conformations. This is supported by experimental chemical shifts which more closely match β-turn values than canonical β-sheet values, although some β-sheet component appears to be present (*SI Appendix,* Table S14) (89). The β-sheet rich hexamer model has Tyr calculated chemical shifts that most closely match the experimental β-sheet component while the trimer following MD most closely matches the RC major component. In contrast, both experimental and predicted data for Gln show limited agreement with any single conformation particularly for Cβ, consistent with its high structural heterogeneity in fibers. These observations suggest that Gln remains largely disordered and underscores the need for further investigation of structurally heterogenous residues. It is also interesting to note that of the large R-group amino acids (Arg, Tyr, and Gln), Gln was the most dynamic in the gland/condensed phases (Fig. 3), ending up the most disordered in the fiber state.

Altogether, these AF3 models—when integrated with MD and SSNMR—offer a structural model of the silk fiber, revealing how poly(Ala) forms stable β-sheets while Arg and Tyr sidechains remain partially disordered but functionally engaged, particularly through persistent cation–π interactions at structured and disordered interfaces. To test whether structural models capture the experimentally observed disorder-to-order transition, we subtracted SHIFTX2-predicted ^13^C chemical shifts from AlphaFold3 fiber models and ColabFold micelle models (Fig. 6*E*). The resulting Δδ values partially mirror experimental shift differences between solution-state and fiber-state NMR (Fig. 5*D*), particularly in β-sheet-promoting residues such as Ala and Gly. Arg exhibits β-sheet character in Cβ and CO, though little change in Cα—possibly due to its interfacial role. Tyr shows modest β-sheet formation consistent with SSNMR data, while Gln displays a surprisingly large shift toward β-sheet values. These findings highlight both the potential and current limitations of predicted chemical shifts in capturing conformational transitions, especially in disordered or heterogeneous systems. This integrated modeling and experimental framework will be extended in future work to examine additional structurally complex residues, including Gln, Ser, and Pro, in the context of phase transitions and hierarchical fiber formation.

## Discussion

In this study, we investigated the molecular mechanisms that govern LLPS and structural transitions in native *L. hesperus* MA silk using a combination of solution NMR, DNP-enhanced MAS SSNMR, MD simulations, and AF3 modeling. We focused on Tyr and Arg residues, which are abundant in Gly-rich domains and hypothesized to mediate LLPS through cation–π interactions. Our results show that potassium phosphate induces LLPS in native MaSp without triggering β-sheet formation in the repetitive core domain. Solution NMR chemical shift perturbations and relaxation measurements indicate that intrinsic disorder is maintained in the condensed phase, with enhanced T_2_ relaxation in Gly-rich regions suggestive of modulated μs–ms dynamics. We also observe moderate rigidification in poly(Ala)-flanked GAA motifs, marked by reduced T_2_ and increased NOE values. These motifs likely serve as nucleation points at the boundary between disordered domains and emerging β-sheet structure.

Unlike prior studies on recombinant constructs that emphasize β-sheet transitions in terminal domains (34), our data demonstrate that the repetitive core of native MaSp remains largely disordered within phosphate-induced condensates. Subtle but reproducible chemical shift changes in polar residues—particularly Arg—highlight sidechain rearrangements rather than backbone folding. Phosphate appears to selectively perturb Arg and Tyr sidechains, while leaving the hydrophobic poly(Ala) core unaffected. This selectivity supports a model in which phosphate promotes LLPS through water exclusion and stabilization of polar interactions, rather than through global secondary structure transitions. Molecular dynamics simulations reinforce this view, showing that phosphate enhances intra- and intermolecular Arg–Tyr interactions while disrupting Arg–poly(Ala) contacts. This behavior aligns with a sticker–spacer framework (65), in which Arg and Tyr act as multivalent stickers that mediate network formation.

DNP-enhanced SSNMR experiments provide direct evidence for Arg–Tyr cation–π interactions in the final spun fiber (Fig. 5*A*). Two-dimensional hNHHC spectra reveal correlations between Tyr aromatic carbons and Arg Nη atoms, consistent with asymmetric, T-shaped geometries. These interactions persist in the solid fiber and are associated with distinct chemical environments for each Nη site. Secondary structure analysis from ^13^C and ^15^N SSNMR reveals that Tyr only exhibits a low degree of β-sheet formation likely adopting β-turn conformations at sheet interfaces, while Arg displays structural heterogeneity with clear contributions from both β-sheet and RC populations. This dual character is consistent with Arg occupying dynamic, interfacial positions—partially incorporated into β-sheet domains while retaining conformational flexibility that enables interaction with Tyr and possibly other polar residues (Fig. 6).

AF3 models corroborate these findings by predicting Arg–Tyr interactions at the boundary of poly(Ala) β-sheet structures, with Arg incorporated into sheet edges and Tyr positioned in β-turns. All-atom MD simulations further support this geometry and capture persistent cation–π contacts, with Nη–ring distances of 4.0 to 4.5 Å in disordered regions. Predicted ^13^C chemical shifts from SHIFTX2 align well with experimental values for β-sheet-associated Ala, Gly, and Arg residues, though some deviations in specific shifts reflect the limitations of solution-trained predictors in modeling tightly packed solid-state β-sheets. Although the overall confidence scores of the AF3 models are relatively low, the strong agreement between SHIFTX2-predicted and experimental chemical shifts supports their use as a valuable starting structure for describing the unstructured region of the silk fiber, which will be further characterized in future work.

Taken together, these results support a cohesive model in which phosphate-induced LLPS in native spider silk is mediated by Arg–Tyr interactions within disordered Gly-rich regions. Upon condensation, the GAA motifs near poly(Ala) domains undergo localized rigidification, forming nucleation sites for β-sheet formation. Following fiber formation, Arg residues span a spectrum of conformations, some integrating at β-sheet interfaces while other disordered Arg environments can still engage in strong cation–π contacts. This combination of interactions in the condensed phase and structural incorporation in the solid fiber illustrates how multivalent sidechain interactions can govern phase behavior, hierarchical assembly, and material properties of structural proteins—a principle that may extend to other biological systems in which organization is preceded by LLPS, such as elastin or nacre. There are, however, some limitations. First, native silk requires selective isotope enrichment to study less abundant residues. While this study focused on Arg–Tyr cation- π interactions, there are likely many other interactions within MaSp that impact protein assembly, such as those involving Gln, Pro, and Ser. In fact, Ser showed one of the larger shifts after inducing LLPS, but was not isotopically enriched and therefore difficult to detect for further analysis. Similarly, MD simulations sampled a small portion of the dominant MaSp1 sequence, allowing analysis of Arg–Tyr interactions since these residues were the focus of this study, but excluding Ser and Pro residues, which are more prevalent in the minor MaSp2 component. Additionally, this study isolates one factor of the silk spinning process (i.e., increased phosphate concentration), but there are several other features involved in fiber formation, including elongational flow and acidic pH. Future studies could incorporate a microfluidic device to investigate how the combination of these factors influence the assembly and hierarchical organization of native spider silk.

Our findings bridge the gap between recombinant and native systems and highlight the value of integrating residue-specific solution NMR, enhanced SSNMR, MD simulations, and deep-learning-based structural models to resolve sequence-encoded assembly mechanisms. Future work will extend this framework to examine transient interactions involving Pro, Ser, and other polar residues, and to probe cooperative LLPS and assembly mechanisms in multicomponent native dopes and recombinant spidroin variants. This strategy may provide a blueprint for understanding phase-coupled fiber assembly in other biological and synthetic IDP-based materials.

## Materials and Methods

Full experimental details are provided in *SI Appendix*. Briefly, isotope-enriched *Latrodectus hesperus* major ampullate (MA) silk was obtained by feeding spiders solutions containing U-[^13^C,^15^N]-enriched Arg and Phe under controlled dietary conditions. LLPS was induced by dialysis of solubilized silk dope against 300 mM potassium phosphate (pH 7.2) in 1 M urea, yielding condensed and dilute phases for NMR analysis. Solution-state NMR experiments were performed at 800 MHz using a TXO cryoprobe to acquire ^13^C/^15^N direct-detect, HSQC, and relaxation datasets. Solid-state and DNP-enhanced MAS NMR spectra were recorded at 600 MHz to probe Arg–Tyr cation–π interactions in fibers. Molecular dynamics simulations (1 µs) were conducted using GROMACS with the CHARMM36m force field, and structural models were generated with ColabFold and AlphaFold3. Predicted chemical shifts were calculated using SHIFTX2 and compared to experimental data.

## Supplementary Material

Appendix 01 (PDF)

## Data Availability

Study data are included in the article and/or *SI Appendix*.

## References

[1] E. Gomes, J. Shorter, The molecular language of membraneless organelles. J. Biol. Chem. **294**, 7115–7127 (2019). 30045872 10.1074/jbc.TM118.001192 PMC6509512

[2] S. F. Banani, H. O. Lee, A. A. Hyman, M. K. Rosen, Biomolecular condensates: Organizers of cellular biochemistry. Nat. Rev. Mol. Cell Biol. **18**, 285–298 (2017). 28225081 10.1038/nrm.2017.7 PMC7434221

[3] S. Boeynaems , Protein phase separation: A new phase in cell biology. Trends Cell Biol. **28**, 420–435 (2018). 29602697 10.1016/j.tcb.2018.02.004 PMC6034118

[4] D. M. Mitrea, R. W. Kriwacki, Phase separation in biology; Functional organization of a higher order. Cell Commun. Signal. **14**, 1 (2016). 26727894 10.1186/s12964-015-0125-7 PMC4700675

[5] A. A. M. André, E. Spruijt, Liquid-liquid phase separation in crowded environments. Int. J. Mol. Sci. **21**, 5908 (2020). 32824618 10.3390/ijms21165908 PMC7460619

[6] S. Y. Bahn, B. H. Jo, Y. S. Choi, H. J. Cha, Control of nacre biomineralization by Pif80 in pearl oyster. Sci. Adv. **3**, e1700765 (2017). 28782039 10.1126/sciadv.1700765 PMC5540247

[7] Q. Gong , Coassembly of a new insect cuticular protein and chitosan via liquid-liquid phase separation. Biomacromolecules **23**, 2562–2571 (2022). 35561014 10.1021/acs.biomac.2c00261

[8] E. W. Martin , Valence and patterning of aromatic residues determine the phase behavior of prion-like domains. Science **367**, 694–699 (2020). 32029630 10.1126/science.aaw8653 PMC7297187

[9] S. Qamar , Fus phase separation is modulated by a molecular chaperone and methylation of arginine cation-π interactions. Cell **173**, 720–734.e715 (2018). 29677515 10.1016/j.cell.2018.03.056 PMC5927716

[10] B. S. Schuster , Identifying sequence perturbations to an intrinsically disordered protein that determine its phase-separation behavior. Proc. Natl. Acad. Sci. U.S.A. **117**, 11421–11431 (2020). 32393642 10.1073/pnas.2000223117 PMC7261017

[11] P. A. Chong, R. M. Vernon, J. D. Forman-Kay, RGG/RG motif regions in RNA binding and phase separation. J. Mol. Biol. **430**, 4650–4665 (2018). 29913160 10.1016/j.jmb.2018.06.014

[12] B. Gabryelczyk , Hydrogen bond guidance and aromatic stacking drive liquid-liquid phase separation of intrinsically disordered histidine-rich peptides. Nat. Commun. **10**, 5465 (2019). 31784535 10.1038/s41467-019-13469-8 PMC6884462

[13] Q. Guo , Hydrogen-bonds mediate liquid-liquid phase separation of mussel derived adhesive peptides. Nat. Commun. **13**, 5771 (2022). 36182948 10.1038/s41467-022-33545-w PMC9526746

[14] S. Amini , A diecast mineralization process forms the tough mantis shrimp dactyl club. Proc. Natl. Acad. Sci. U.S.A. **116**, 8685–8692 (2019). 30975751 10.1073/pnas.1816835116 PMC6500109

[15] S. Winkler, D. L. Kaplan, Molecular biology of spider silk. Rev. Mol. Biotechnol. **74**, 85–93 (2000). 10.1016/s1389-0352(00)00005-2 11763505

[16] D. P. Knight, M. M. Knight, F. Vollrath, Beta transition and stress-induced phase separation in the spinning of spider dragline silk. Int. J. Biol. Macromol. **27**, 205–210 (2000). 10828366 10.1016/s0141-8130(00)00124-0

[17] E. K. Tillinghast, S. F. Chase, M. A. Townley, Water extraction by the major ampullate duct during silk formation in the spider, Argiope aurantia Lucas. J. Insect Physiol. **30**, 591–596 (1984).

[18] T. Scheibel, Spider silks: Recombinant synthesis, assembly, spinning, and engineering of synthetic proteins. Microb. Cell Fact. **3**, 14 (2004). 15546497 10.1186/1475-2859-3-14 PMC534800

[19] P. R. Laity, G. Dunderdale, O. O. Mykhaylyk, C. Holland, Flow-induced protein chain deformation, segmental orientation, and phase separation in native silk feedstock. Biomacromolecules **24**, 2828–2846 (2023). 37234047 10.1021/acs.biomac.3c00233 PMC10265709

[20] D. H. Hijirida , 13C NMR of Nephila clavipes major ampullate silk gland. Biophys. J. **71**, 3442–3447 (1996). 8968613 10.1016/S0006-3495(96)79539-5 PMC1233831

[21] M. Hronska, J. D. van Beek, P. T. Williamson, F. Vollrath, B. H. Meier, NMR characterization of native liquid spider dragline silk from *Nephila edulis*. Biomacromolecules **5**, 834–839 (2004). 15132669 10.1021/bm0343904

[22] J. E. Jenkins, G. P. Holland, J. L. Yarger, High resolution magic angle spinning NMR investigation of silk protein structure within major ampullate glands of orb weaving spiders. Soft Matter **8**, 1947–1954 (2012).

[23] D. P. Knight, F. Vollrath, Liquid crystals and flow elongation in a spider’s silk production line. Proc. R. Soc. Lond. Series B: Biol. Sci. **266**, 519–523 (1999).

[24] F. Vollrath, D. P. Knight, Liquid crystalline spinning of spider silk. Nature **410**, 541–548 (2001). 11279484 10.1038/35069000

[25] D. P. Knight, F. Vollrath, Changes in element composition along the spinning duct in a Nephila spider. Naturwissenschaften **88**, 179–182 (2001). 11480706 10.1007/s001140100220

[26] E. Doblhofer, A. Heidebrecht, T. Scheibel, To spin or not to spin: Spider silk fibers and more. Appl. Microbiol. Biotechnol. **99**, 9361–9380 (2015). 26362683 10.1007/s00253-015-6948-8

[27] A. Koeppel, C. Holland, Progress and trends in artificial silk spinning: A systematic review. ACS Biomater. Sci. Eng. **3**, 226–237 (2017). 33465923 10.1021/acsbiomaterials.6b00669

[28] D. Qin, J. Li, H. Li, H. Zhang, K. Liu, Engineered spidroin-derived high-performance fibers for diverse applications. Nano Res. **17**, 492–502 (2024).

[29] A. Rising, J. Johansson, Toward spinning artificial spider silk. Nat. Chem. Biol. **11**, 309–315 (2015). 25885958 10.1038/nchembio.1789

[30] L. Zeußel, H. Bargel, G. P. Holland, T. Scheibel, Liquid-liquid phase separation of spider silk proteins. Polym. J. **57**, 831–843 (2025), 10.1038/s41428-025-01039-3.

[31] M. Landreh , Liquid-liquid crystalline phase separation of spider silk proteins. Commun. Chem. **7**, 260 (2024). 39533043 10.1038/s42004-024-01357-2 PMC11557605

[32] M. G. Lay, N. A. Oktaviani, A. D. Malay, K. Numata, Exploring the self-assembly of silk proteins through liquid-liquid phase separation. Polym. J. **57**, 799–814 (2025), 10.1038/s41428-025-01040-w.

[33] A. D. Malay , Spider silk self-assembly via modular liquid-liquid phase separation and nanofibrillation. Sci. Adv. **6**, eabb6030 (2020). 33148640 10.1126/sciadv.abb6030 PMC7673682

[34] P. Mohammadi , Controllable coacervation of recombinantly produced spider silk protein using kosmotropic salts. J. Colloid Interface Sci. **560**, 149–160 (2020). 31670097 10.1016/j.jcis.2019.10.058

[35] U. K. Slotta, S. Rammensee, S. Gorb, T. Scheibel, An engineered spider silk protein forms microspheres. Angew. Chem. Int. Ed. Engl. **47**, 4592–4594 (2008). 18461576 10.1002/anie.200800683

[36] J. H. Exler, D. Hümmerich, T. Scheibel, The amphiphilic properties of spider silks are important for spinning. Angew. Chem. Int. Ed. Engl. **46**, 3559–3562 (2007). 17397124 10.1002/anie.200604718

[37] A. D. Malay, N. A. Oktaviani, J. Chen, K. Numata, Spider silk: Rapid, bottom-up self-assembly of MaSp1 into hierarchically structured fibers through biomimetic processing. Adv. Funct. Mater. **35**, 2408175 (2025).

[38] N. A. Oktaviani, A. Matsugami, F. Hayashi, K. Numata, Ion effects on the conformation and dynamics of repetitive domains of a spider silk protein: Implications for solubility and β-sheet formation. Chem. Commun. **55**, 9761–9764 (2019). 10.1039/c9cc03538a 31355386

[39] L. Eisoldt, J. G. Hardy, M. Heim, T. R. Scheibel, The role of salt and shear on the storage and assembly of spider silk proteins. J. Struct. Biol. **170**, 413–419 (2010). 20045467 10.1016/j.jsb.2009.12.027

[40] D. Huemmerich , Primary structure elements of spider dragline silks and their contribution to protein solubility. Biochemistry **43**, 13604–13612 (2004). 15491167 10.1021/bi048983q

[41] V. Hovanová, A. Hovan, M. Humenik, E. Sedlák, Only kosmotrope anions trigger fibrillization of the recombinant core spidroin eADF4(C16) from Araneus diadematus. Protein Sci. **32**, e4832 (2023). 37937854 10.1002/pro.4832 PMC10661072

[42] M. Humenik, A. M. Smith, S. Arndt, T. Scheibel, Ion and seed dependent fibril assembly of a spidroin core domain. J. Struct. Biol. **191**, 130–138 (2015). 26123261 10.1016/j.jsb.2015.06.021

[43] A. Heidebrecht , Biomimetic fibers made of recombinant spidroins with the same toughness as natural spider silk. Adv. Mater. **27**, 2189–2194 (2015). 25689835 10.1002/adma.201404234

[44] D. Stengel , Tyrosine’s unique role in the hierarchical assembly of recombinant spider silk proteins: From spinning dope to fibers. Biomacromolecules **24**, 1463–1474 (2023). 36791420 10.1021/acs.biomac.2c01467

[45] N. A. Ayoub, J. E. Garb, R. M. Tinghitella, M. A. Collin, C. Y. Hayashi, Blueprint for a high-performance biomaterial: Full-length spider dragline silk genes. PLoS One **2**, e514 (2007). 17565367 10.1371/journal.pone.0000514 PMC1885213

[46] M. S. Creager , Solid-state NMR comparison of various spiders’ dragline silk fiber. Biomacromolecules **11**, 2039–2043 (2010). 20593757 10.1021/bm100399x PMC2922512

[47] D. Xu, J. L. Yarger, G. P. Holland, Exploring the backbone dynamics of native spider silk proteins in Black Widow silk glands with solution-state NMR spectroscopy. Polymer **55**, 3879–3885 (2014).

[48] L. R. Parent , Hierarchical spidroin micellar nanoparticles as the fundamental precursors of spider silks. Proc. Natl. Acad. Sci. U.S.A. **115**, 11507–11512 (2018). 30348773 10.1073/pnas.1810203115 PMC6233143

[49] G. P. Holland, M. S. Creager, J. E. Jenkins, R. V. Lewis, J. L. Yarger, Determining secondary structure in spider dragline silk by carbon-carbon correlation solid-state NMR spectroscopy. J. Am. Chem. Soc. **130**, 9871–9877 (2008). 18593157 10.1021/ja8021208

[50] G. M. Gray , Secondary structure adopted by the Gly-Gly-X repetitive regions of dragline spider silk. Int. J. Mol. Sci. **17**, 2023 (2016). 27918448 10.3390/ijms17122023 PMC5187823

[51] J. D. van Beek, S. Hess, F. Vollrath, B. H. Meier, The molecular structure of spider dragline silk: Folding and orientation of the protein backbone. Proc. Natl. Acad. Sci. U.S.A. **99**, 10266–10271 (2002). 12149440 10.1073/pnas.152162299 PMC124902

[52] K. Chalek, A. Soni, C. D. Lorenz, G. P. Holland, Proline-tyrosine ring interactions in black widow dragline silk revealed by solid-state nuclear magnetic resonance and molecular dynamics simulations. Biomacromolecules **25**, 1916–1922 (2024). 38315982 10.1021/acs.biomac.3c01351

[53] M. Schiavina , Intrinsically disordered proteins studied by NMR spectroscopy. J. Magn. Reson. Open **18**, 100143 (2024).

[54] M. Mirdita , ColabFold: Making protein folding accessible to all. Nat. Methods **19**, 679–682 (2022). 35637307 10.1038/s41592-022-01488-1 PMC9184281

[55] J. Abramson , Accurate structure prediction of biomolecular interactions with AlphaFold 3. Nature **630**, 493–500 (2024). 38718835 10.1038/s41586-024-07487-w PMC11168924

[56] B. Han, Y. Liu, S. W. Ginzinger, D. S. Wishart, SHIFTX2: Significantly improved protein chemical shift prediction. J. Biomol. NMR **50**, 43–57 (2011). 21448735 10.1007/s10858-011-9478-4 PMC3085061

[57] R. P. Joosten , A series of PDB related databases for everyday needs. Nucleic Acids Res. **39**, D411–D419 (2011). 21071423 10.1093/nar/gkq1105 PMC3013697

[58] W. Kabsch, C. Sander, Dictionary of protein secondary structure: Pattern recognition of hydrogen-bonded and geometrical features. Biopolymers **22**, 2577–2637 (1983). 6667333 10.1002/bip.360221211

[59] K. A. Burke, A. M. Janke, C. L. Rhine, N. L. Fawzi, Residue-by-residue view of in vitro FUS granules that bind the C-terminal domain of RNA polymerase II. Mol. Cell **60**, 231–241 (2015). 26455390 10.1016/j.molcel.2015.09.006 PMC4609301

[60] A. C. Murthy , Molecular interactions underlying liquid−liquid phase separation of the FUS low-complexity domain. Nat. Struct. Mol. Biol. **26**, 637–648 (2019). 31270472 10.1038/s41594-019-0250-x PMC6613800

[61] D. T. Murray , Structure of FUS protein fibrils and its relevance to self-assembly and phase separation of low-complexity domains. Cell **171**, 615–627.e616 (2017). 28942918 10.1016/j.cell.2017.08.048 PMC5650524

[62] T. J. Nott , Phase transition of a disordered nuage protein generates environmentally responsive membraneless organelles. Mol. Cell **57**, 936–947 (2015). 25747659 10.1016/j.molcel.2015.01.013 PMC4352761

[63] J. P. Brady , Structural and hydrodynamic properties of an intrinsically disordered region of a germ cell-specific protein on phase separation. Proc. Natl. Acad. Sci. U.S.A. **114**, E8194–E8203 (2017). 28894006 10.1073/pnas.1706197114 PMC5625912

[64] S. E. Reichheld, L. D. Muiznieks, F. W. Keeley, S. Sharpe, Direct observation of structure and dynamics during phase separation of an elastomeric protein. Proc. Natl. Acad. Sci. U.S.A. **114**, E4408–E4415 (2017). 28507126 10.1073/pnas.1701877114 PMC5465911

[65] J.-M. Choi, A. S. Holehouse, R. V. Pappu, Physical principles underlying the complex biology of intracellular phase transitions. Annu. Rev. Biophys. **49**, 107–133 (2020). 32004090 10.1146/annurev-biophys-121219-081629 PMC10715172

[66] J. Wang , A molecular grammar governing the driving forces for phase separation of prion-like RNA binding proteins. Cell **174**, 688–699.e616 (2018). 29961577 10.1016/j.cell.2018.06.006 PMC6063760

[67] R. M. Vernon , Pi-Pi contacts are an overlooked protein feature relevant to phase separation. eLife **7**, e31486 (2018). 29424691 10.7554/eLife.31486 PMC5847340

[68] A. Leppert , Liquid-liquid phase separation primes spider silk proteins for fiber formation via a conditional sticker domain. Nano Lett. **23**, 5836–5841 (2023). 37084706 10.1021/acs.nanolett.3c00773 PMC10311596

[69] D. S. Wishart, B. D. Sykes, F. M. Richards, Relationship between nuclear magnetic resonance chemical shift and protein secondary structure. J. Mol. Biol. **222**, 311–333 (1991). 1960729 10.1016/0022-2836(91)90214-q

[70] D. S. Wishart, B. D. Sykes, F. M. Richards, The chemical shift index: A fast and simple method for the assignment of protein secondary structure through NMR spectroscopy. Biochemistry **31**, 1647–1651 (1992). 1737021 10.1021/bi00121a010

[71] D. Onofrei , Investigating the atomic and mesoscale interactions that facilitate spider silk protein pre-assembly. Biomacromolecules **22**, 3377–3385 (2021). 34251190 10.1021/acs.biomac.1c00473

[72] L. E. Kay, D. A. Torchia, A. Bax, Backbone dynamics of proteins as studied by nitrogen-15 inverse detected heteronuclear NMR spectroscopy: Application to staphylococcal nuclease. Biochemistry **28**, 8972–8979 (1989). 2690953 10.1021/bi00449a003

[73] L. R. Becerra, G. J. Gerfen, R. J. Temkin, D. J. Singel, R. G. Griffin, Dynamic nuclear polarization with a cyclotron resonance maser at 5 T. Phys. Rev. Lett. **71**, 3561–3564 (1993). 10055008 10.1103/PhysRevLett.71.3561

[74] D. A. Hall , Polarization-enhanced NMR spectroscopy of biomolecules in frozen solution. Science **276**, 930–932 (1997). 9139651 10.1126/science.276.5314.930

[75] M. Rosay , Solid-state dynamic nuclear polarization at 263 GHz: Spectrometer design and experimental results. Phys. Chem. Chem. Phys. **12**, 5850–5860 (2010). 20449524 10.1039/c003685b PMC4442492

[76] H. C. Craig , DNP NMR spectroscopy reveals new structures, residues and interactions in wild spider silks. Chem. Commun. **55**, 4687–4690 (2019). 10.1039/c9cc01045a 30938741

[77] T. Maly , Dynamic nuclear polarization at high magnetic fields. J. Chem. Phys. **128**, 052211 (2008). 18266416 10.1063/1.2833582 PMC2770872

[78] J. P. Gallivan, D. A. Dougherty, Cation-π interactions in structural biology. Proc. Natl. Acad. Sci. U.S.A. **96**, 9459–9464 (1999). 10449714 10.1073/pnas.96.17.9459 PMC22230

[79] D. T. Infield , Cation-π interactions and their functional roles in membrane proteins. J. Mol. Biol. **433**, 167035 (2021). 33957146 10.1016/j.jmb.2021.167035 PMC8338773

[80] Y. Wang, O. Jardetzky, Probability-based protein secondary structure identification using combined NMR chemical-shift data. Protein Sci. **11**, 852–861 (2002). 11910028 10.1110/ps.3180102 PMC2373532

[81] K. J. Fritzsching, Y. Yang, K. Schmidt-Rohr, M. Hong, Practical use of chemical shift databases for protein solid-state NMR: 2D chemical shift maps and amino-acid assignment with secondary-structure information. J. Biomol. NMR **56**, 155–167 (2013). 23625364 10.1007/s10858-013-9732-z PMC4048757

[82] T. Izdebski, P. Akhenblit, J. E. Jenkins, J. L. Yarger, G. P. Holland, Structure and dynamics of aromatic residues in spider silk: 2D carbon correlation NMR of dragline fibers. Biomacromolecules **11**, 168–174 (2010). 19894709 10.1021/bm901039e

[83] J. E. Jenkins , Characterizing the secondary protein structure of Black Widow dragline silk using solid-state NMR and X-ray diffraction. Biomacromolecules **14**, 3472–3483 (2013). 24024617 10.1021/bm400791u PMC3914425

[84] J. E. Jenkins , Solid-state NMR evidence for elastin-like β-turn structure in spider dragline silk. Chem. Commun. **46**, 6714–6716 (2010). 10.1039/c0cc00829j 20733981

[85] M. Hong, D. Isailovic, R. A. McMillan, V. P. Conticello, Structure of an elastin-mimetic polypeptide by solid-state NMR chemical shift analysis. Biopolymers **70**, 158–168 (2003). 14517905 10.1002/bip.10431

[86] D. S. Wishart, C. G. Bigam, A. Holm, R. S. Hodges, B. D. Sykes, 1H, 13C and 15N random coil NMR chemical shifts of the common amino acids. I. Investigations of nearest-neighbor effects. J. Biomol. NMR **5**, 67–81 (1995). 7881273 10.1007/BF00227471

[87] A. A. Shcherbakov, J. Medeiros-Silva, N. Tran, M. D. Gelenter, M. Hong, From angstroms to nanometers: Measuring interatomic distances by solid-state NMR. Chem. Rev. **122**, 9848–9879 (2022). 34694769 10.1021/acs.chemrev.1c00662 PMC9035484

[88] A. Lange, S. Luca, M. Baldus, Structural constraints from proton-mediated rare-spin correlation spectroscopy in rotating solids. J. Am. Chem. Soc. **124**, 9704–9705 (2002). 12175218 10.1021/ja026691b

[89] C.-C. Wang, W.-C. Lai, W.-J. Chuang, Type I and II β-turns prediction using NMR chemical shifts. J. Biomol. NMR **59**, 175–184 (2014). 24838372 10.1007/s10858-014-9837-z

