# Supplementary material for: Arg–Tyr cation–π interactions drive phase separation and β-sheet assembly in native spider dragline silk
Source: Proc Natl Acad Sci U S A. 2025 Dec 23;122(52):e2523198122. doi: 10.1073/pnas.2523198122 (PMC12772222; doi:10.1073/pnas.2523198122)
Supplement: Supplementary file 1 — Appendix 01 (PDF) [file pnas.2523198122.sapp.pdf]

PNAS

Supporting Information for

Arg-Tyr Cation-π Interactions Drive Phase Separation and β-sheet Assembly in Native Spider Dragline Silk

Hannah R. Johnson[1]† , Kevin Chalek[1]†, Nesreen Elathram[2], Andy T. Chau[1], Anikin Rae Domingo[1], Julian E. Aldana[1], Hieu Nguyen[1], Alexia de Loera[1], Brianna A. Duarte[1], Lado Shapakidze[1], David Onofrei[1], Galia T. Debelouchina[2], Christian D. Lorenz[3], Gregory P. Holland[1,*]

*Gregory P. Holland
Email: gholland@sdsu.edu

Table of Contents

Materials and Methods.......3-6

SI Figures
Fig. S1. X-detect experiments for condensed phase allow assignment of overlapping signals 7
Fig. S2. 13C NADEQUATE for intact glands allows assignment of directly bound carbons 8
Fig. S3. CBCA(CO)NH and HNCACB allow assignment of amide resonances 9
Fig. S4. 13C chemical shift differences indicate spinning dope maintains random coil structure 10
Fig. S5. Representative fits for 15N T1 and T2 relaxation measurements 11
Fig. S6. 13C NOESY-HSQC collected on intact glands and condensed phase 12
Fig. S7. Initial conformation of hexamer peptide generated by ColabFold 13
Fig. S8. Molecular dynamics simulation of six MaSp1 fragments in urea 14
Fig. S9. DNP enhancement in 13C and 15N CP-MAS spectra 15
Fig. S10. 2D DNP-MAS 15N–13C DCP NCα spectrum 16
Fig. S11. 2D DNP-MAS 15N–13C TEDOR spectrum 17
Fig. S12. 2D 13C–13C DARR SSNMR spectrum (Arg-labeled spider silk, 100 ms) 18
Fig. S13. 2D 13C–13C DARR SSNMR spectrum (Arg-labeled spider silk, 500 ms) 19
Fig. S14. Blow-up of Arg Cα and Cβ sites from Fig. S13 20
Fig. S15. Blow-up of Tyr Cα/Cβ sites from 2D DARR spectrum 21
Fig. S16. Full projection of Tyr resonances from Fig. S15 22
Fig. S17. Water-suppressed 1H spectra over time show sample stability 23

Fig. S18. EPR experiments with different dragline silks and radicals .... ……………………………………………….24

SI Tables

Table S1. 15N chemical shifts from solution NMR spectra 25-26
Table S2. 1H chemical shifts from HSQC solution spectra .... ……………………………………………27
Table S3. 13C chemical shifts from direct-detect and HSQC spectra 28-29
Table S4. 1H chemical shifts from HSQC spectra .... ……………………………………..30-31
Table S5. 15N T1 relaxation times .... ……………………………………….…..32
Table S6. 15N T2 relaxation times .... ……………………………………….…..33
Table S7. 1H–15N hNOE values .... ……………………………………………34
Table S8. DSSP analysis of simulated 116-residue MaSp1 hexamer assembly .... ……………………………………………..35
Table S9. Comparison of predicted vs experimental 15N chemical shifts .... ……………………………………………36
Table S10. Comparison of predicted vs experimental NH chemical shifts .... ……………………………………………37
Table S11. Comparison of predicted vs experimental 13C chemical shifts 38-39
Table S12. Comparison of predicted vs experimental 1H chemical shifts .... ……………………………………………40
Table S13. Comparison of solid-state vs solution chemical shifts at RT and DNP temperatures 41-42
Table S14. 13C chemical shifts measured for silk fiber vs solution states 43-44
Table S15. 15N chemical shifts measured for silk fiber vs solution states 45-46
Table S16. DSSP analysis of AF3 β-sheet model .... ……………………………………………47
Table S17. Predicted vs experimental 13C chemical shifts for AF3 models .... …………………………………………48-49
Table S18. Predicted vs experimental 15N chemical shifts for AF3 models 50
Table S19. DSSP quantification of AF3 trimer model 51
Table S20. DSSP quantification of AF3 trimer model after MD .... ……………………………………………52
Table S21. Spectral density functions (J) calculated at 800 MHz from 15N Relaxation Data…………………………………………………………53-54

SI References 55-56

Materials and Methods

Isotope-Enriched Spider Silk Preparation. To isotope enrich Arg and Tyr in *L. hesperus* MA silk, spiders were fed a solution containing U-[^{13}C/^{15}N]-enriched Arg and Phe adjusted to pH 7.4 using sodium bicarbonate. When these amino acids are metabolized by the spider, they are incorporated into more abundant residues such as Gly, Ala, and Pro. Unenriched Ala and Pro were added to the feeding solution to minimize isotope scrambling and increase the labeling efficiency of Arg and Tyr. Spiders were fed this solution three times a week while forcibly silking MA silk for one hour. At the end of two weeks, spiders were sacrificed and MA glands removed for NMR studies as previously described (1). Silk fiber samples were also prepared by feeding a solution containing U-[^{13}C/^{15}N]-enriched Phe, Ala, and Arg. Another set of silk fiber samples were prepared by feeding a solution containing U-[^{13}C/^{15}N]-enriched Arg and unenriched Ala, Pro, and Gly to target Arg.

To prepare gland samples for NMR, intact glands were added directly to a 5 mm Shigemi tube filled with 90:10 H_2O:D_2O and 6 mM DSS. Excess solution was removed from the NMR tube after all glands were added. For 1 M urea and LLPS samples, the outer membrane of the MA gland was removed as previously described to expose the protein dope (2). The dope was solubilized in freshly prepared 4 M urea with pH adjusted to 7.2 using 1 M HCl. Glands were placed in separate solutions of 4 M urea to prevent aggregation and solubilized for four hours at 4 °C. After solubilizing, the solutions were combined, transferred to a dialysis bag by slowly pipetting with a borosilicate glass pipette to minimize shearing. For the 1 M urea sample, glands were dialyzed for 1 hour in 1 M urea, pH 7.2, and an additional hour in 1 M urea, 90:10 H_2O:D_2O, 1 mM DSS, pH 7.2. This sample was transferred to a 5 mm Shigemi tube. For the LLPS sample, glands were dialyzed against two solutions of 1 M urea for 1 hour each, and a third solution of 300 mM phosphate, 1 M urea, 90:10 H_2O:D_2O, 1 mM DSS, pH 7.2 for 30-45 minutes until a cloudy phase formed, indicating turbid LLPS droplets. Following phase separation, both condensed and dilute phases were collected separately. The condensed phase was used for detailed multi-nuclear, multi-dimensional NMR and relaxation measurements due to its higher protein concentration, whereas the dilute phase was too low in protein content for complete multidimensional or relaxation analysis and was therefore limited to ^{13}C direct-detect and HSQC for chemical shift comparison (***SI Appendix***, **Fig. S4** and **Table S3**).

The solution was transferred to a 3 mm Shigemi tube, gently flicked to remove air bubbles, and left at room temperature overnight to allow the turbid phase to settle into a condensed phase. The following morning, the dilute phase was removed, transferred to a separate NMR tube, and stored in the fridge until data collection.

Optical Microscopy. Black widow MA silk was solubilized in 2 M urea, pH 7, and transferred into a 96 well plate before exposing to potassium phosphate and imaging LLPS. Images were collected on a Keyence light microscope in SDSU's Electron Microscopy facility.

Protein Solution NMR Data Collection and Processing. Spectra were collected at 298 K using a Bruker Avance Neo 800 MHz NMR with a triple resonance TXO cryoprobe. Initial water-suppressed ^{1}H spectra were collected with 64 scans, while following water-suppressed ^{1}H spectra, used to track sample stability, were collected with 8 scans (***SI Appendix,*** **Fig. S17**). ^{13}C direct-detect spectra were collected with 5k scans for the intact glands, 1 M urea, and condensed phase and 10k scans for the dilute phase. 2D ^{1}H-^{13}C HSQC spectra (hsqcetgpg) were collected with 1024 x 512 points for t_1 x t_2 dimensions with 16 scans per point, 30% non-uniform sampling (NUS) with ^{13}C decoupling applied through the pulse sequence (-DLABEL_CN). ^{15}N direct-detect spectra were collected with 3k scans. 2D ^{1}H-^{15}N HSQC spectra (hsqcfpf3gpphwg) were collected with 2048 x 512 points for t_1 x t_2 dimensions with 32 scans per point, 50% non-uniform sampling (NUS) with ^{13}C decoupling applied through the pulse sequence (-DLABEL_CN). For X-detect experiments, CACO was collected with 1024 x 128 points with 32 scans per point, CAN was collected with 1024 x 64 points with 32 scans per point, CON was collected with 1024 x 32 points, 512 scans per point, each with 50% NUS. ^{1}H-^{15}N relaxation

experiments (T_1, T_2, and NOE) were collected using pseudo-3D experiments (3) with a 3 s recycle delay, 2048 x 512 points for t_1 x t_2 dimensions, and 50% non-uniform sampling. For T_1, 13 ^{1}H-^{15}N HSQC spectra were collected with 8 scans per point and delay periods ranging from 20 ms to 5000 ms. For T_2, 16 ^{1}H-^{15}N HSQC spectra were collected with 4 scans per point and delay periods ranging from 34 ms to 2240 ms. For hNOE, two ^{1}H-^{15}N HSQC spectra were collected with 16 scans both with and without proton saturation for 3s. CBCA(CO)NH was collected with 2048 x 128 x 128 points for t_1 x t_2 x t_3 dimensions, 16 scans per point with 25% NUS. HNCACB was collected with 2048 x 80 x 100 points for t_1 x t_2 x t_3 dimensions, 32 scans per point with 25% NUS. INADEQUATE was collected with 2000 x 256 points for t_1 x t_2 dimensions, 128 scans per point with 50% NUS. ^{13}C NOESY-HSQC (noesyhsqcetgp3d) was collected 2048 x 64 x 128 points for t_1 x t_2 x t_3 dimensions, 8 scans per point with 25% NUS at 500 ms mixing time.

^{13}C direct-detect spectra were processed in Topspin 4.0. ^{15}N direct-detect spectra were processed in MestReNova. 2D/3D data sets were processed in NMRpipe (4). T_1/T_2 relaxation data was processed using Dynamics Center in Topspin 4.0. NOE data was processed in NMRpipe and hNOE was calculated in relax (5). Spectral density functions were calculated from site-specific ^{15}N relaxation parameters (R_1, R_2, and hNOE) measured at 800 MHz following the formalism of Farrow *et al.* (1995) (6). The reduced spectral density approach was used to estimate J(0), J(ωN), and J(ωH) from the experimental relaxation data according to equations (5–7) in that work, where J(ω) represents the frequency-dependent spectral density function describing the amplitude of backbone N–H vector motions. The assumption is that the high frequency spectral density terms that contribute to the relaxation processes are of approximately equal magnitude, i.e., $J(\omega H \pm \omega N) \approx J(\omega H)$, and therefore may be replaced by a single equivalent term J(ωh). Data were analyzed residue by residue for each sample condition (intact, condensed, and urea-solubilized). The mapping was implemented in Python following the standard analytical framework and tabulated in *SI Appendix,* Table S21. Residues from INADEQUATE, HNCACB, CBCA(CO)NH, CON, CAN, and CACO experiments were assigned in Poky (7). All chemical shifts were referenced to DSS.

Solid-State NMR Spectroscopy. Solid-state NMR experiments were performed using a 600 MHz (14.1 Tesla) Bruker AVANCE-IIIHD spectrometer equipped with a Bruker 1.9 mm HCN MAS probe. 2D DARR experiments (8-12) were conducted while spinning at 10 or 15 kHz and collected with 512 scans and 64 t1 points in the indirect dimension (2 ms acquisition time) with mixing times of 100 ms and 500 ms. The recycle delay was 2.5 s and there were 1024 t2 acquisition points (12 ms acquisition time). SWFTPPM ^{1}H decoupling with an 87 kHz rf field strength was applied during acquisition with a 13° phase shift (13). ^{13}C chemical shifts were referenced externally to TMS at 0.0 ppm by setting the downfield adamantane signal to 38.48 ppm (14). All reported ^{13}C chemical shifts were adjusted to the DSS scale (14). ^{15}N chemical shifts were referenced to solid ^{15}N-Gly (33.4 ppm) (15).

Dynamic Nuclear Polarization Solid-state NMR Spectroscopy. To optimize DNP performance for silk fibers, we tested three radical/solvent combinations to improve radical distribution: 10 mM AMUPol in a 60:35:5 (v/v/v) ratio of glycerol-d8/D_2O/H_2O, 10 mM AsymPolPOK in a 30:60:10 (v/v/v) ratio of glycerol-d8/D_2O/H_2O, and 10 mM AMUPol in a 80:20 (v/v) ratio of D_2O/H_2O. The greatest level of DNP enhancement among these samples was achieved when 4.4 mg of U-[^{13}C/^{15}N]-Phe, -Ala, and -Arg labeled *L. hesperus* MA silk fibers was fluffed up by hand and 15 mL of 10 mM AMUPol in 80/20 D_2O/H_2O solvent was added dropwise to the silk. EPR spectroscopy was used to determine a final AMUPol concentration of 19 mM in the rotor (*SI Appendix*, Fig. S18). The sample was then mixed to integrate the solvent. The sample was kept in a cold room at 4 °C for 22 hours before packing into a 1.9 mm rotor. DNP experiments were conducted with a 600 MHz Bruker DNP NMR spectrometer equipped with a NEO console and a 395 GHz gyrotron for high-power microwave irradiation. A triple resonance HCN 1.9 mm low temperature MAS probe was used to record all DNP spectra. The MAS frequency was set to 15 kHz and spectra were referenced to the 40.49 ppm ^{13}C peak of adamantane at room temperature or to 3.2 ppm signal of the silicon plug at 100 K (14). The DNP enhancement was measured by acquiring spectra with and

without microwave irradiation at 5 W. 2D DCP ^{15}N-^{13}C HETCOR were recorded with 32 scans, d1 = 5 s, 128 t_1 pts in the indirect dimension (8 ms acquisition time), 1536 t2 pts in the direct dimension (17 ms acquisition time), 4 ms N-C CP contact time with the ^{13}C carrier set to 55 ppm for N-Ca correlations. 2D TEDOR experiments (16) were collected with 384 scans, d1 = 5 s, 128 t_1 pts in the indirect dimension (5 ms acquisition time), 1024 t2 pts in the direct dimension (10 ms acquisition time), 4.2 ms mixing and 16 rotor periods. 2D hNHHC experiments(17) were recorded with 160 scans, d1 = 8 s, 72 t_1 pts in the indirect dimension (3 ms acquisition time), 1024 t2 pts in the direct dimension (10 ms acquisition time), and 500 ms mixing. ^{1}H, ^{13}C and ^{15}N pulses were set to 3 ms, 3 ms and 5 ms, respectively.

EPR Spectroscopy. EPR experiments were performed on a 9.3 GHz Bruker EMXplus X-band CW EPR spectrometer equipped with a standard high sensitivity resonator. Sample-packed DNP rotors were placed into a standard X-band 707-SQ-250M EPR tube (Wilmad Labglass). To perform the measurements, the rotor was placed into a standard X-band 707-SQ-250M EPR tube (Wilmad Labglass). 1D EPR spectra were acquired with the following parameters: 10 mW power, 200 G sweep width, 30 s sweep time and 1 scan. For spin quantification, double integration was calculated, and the integrated intensity was compared to a standard curve prepared with rotors containing known concentrations of AMUPol/AsymPolPOK to determine the final AMUPol concentration of 19 mM in the rotor in the sample used in the main text. (***SI Appendix*****, Fig. S18**). EPR analysis was performed using EasySpin (18).

Molecular dynamics simulations. In order to generate a model of a micelle-like aggregate of *L. hesperus* MaSp1, we used ColabFold (19) to predict the structure of a hexamer of a peptide with the following sequence: *AAAAAAAAGGAGQGGQGGYGQGGYGQGGAGQGGAAAAAAAAGGAGQGGYGRGGAGQGGAAAAAGAGQGGYGGQGAGQGGAGAAAAAAAA GGAGQGGQGGYGRGGYGQGGAGQGGAG* (***SI Appendix*****, Fig. S7**). The rank 1 model (**Fig. 4**) was used as the initial conformation for our simulations. All of the simulations reported in this manuscript were performed with GROMACS 2019 (20).

The initial structures of our peptides were then inputted into the CHARMM-GUI Solution Builder (21, 22) in order to build our simulated system and the corresponding files required to perform the simulations in GROMACS. In doing so, we generated a simulation box of size 92 Å x 92 Å x 92 Å that contained the peptides and 21,367 water molecules, which resulted in a total of 71,499 atoms. Three different ionic solutions were investigated, 0.2 M NaCl, 0.2 M KH_2PO_4 and 1.0 M urea. The CHARMM36m forcefield (23) was used to describe the proteins, ions and water in the simulations. The LJ interactions were cut-off at 12 Å, and the PME algorithm was used for the long-range Coloumb's interactions. The simulation protocol suggested by CHARMM-GUI was used, which consists of the following steps: (i) a steepest descent energy minimization which runs until there is a convergence with a maximum force on any atom less than 1 000 kJ; (ii) an NVT simulation using the Nose-Hoover thermostat (24, 25) to control the temperature at a target temperature of 303.15 K that lasted 125 ps and utilized a 1 fs timestep; and (iii) the production simulation which employed the NPT ensemble with a Nose-Hoover thermostat and a Parrinello-Rahman barostat (26, 27) to control the temperature and pressure, respectively. The target pressure was 1 bar and the target temperature in the production simulation was the same as the NVT simulation, 303.15 K. Different thermostats were used to control the temperatures of the water and the peptides separately. The production simulation used a 2 fs timestep and in total the simulation was conducted for 1 µs. All analysis was done on the trajectory of the production simulation using a combination of MDAnalysis (28, 29) and in-house Python scripts.

AlphaFold3 structure analysis. AlphaFold3(30) was used to create a trimer of the peptide with a sequence of "AAAAAAAAGGAGQGGQGGYGRGGYGQGGAGQGGAGAAAAAAAA" (Fig. 6B, pTM = 0.67) and hexamer:AAAAAAAAGGAGQGGQGGYGQGGYGQGGAGQGGAAAAAAAAGGAGQGGYGRGGAGQGGAAAAAGAGQGGYGGQGAGQGGAGA AAAAAAAGGAGQGGQGGYGRGGYGQGGAGQGGAG (Fig. 6A, pTM = 0.33) from *L. hesperus* MaSp1 (31). The trimer predicted by AlphaFold3

displays lower overall β-sheet content relative to the hexamer, which arises directly from its sequence composition: the trimer construct contains fewer contiguous poly(Ala) segments and a higher proportion of GGX motifs that disfavor extended β-sheet formation. Thus, the reduced β-sheet character reflects intrinsic sequence heterogeneity rather than any modeling constraint.

Visualizations and distance measurements were conducted in PyMOL (32). The trimer model was further refined using all-atom MD simulations with the CHARMM36m force field in explicit water (no added ions) for 200 ns, as described above.

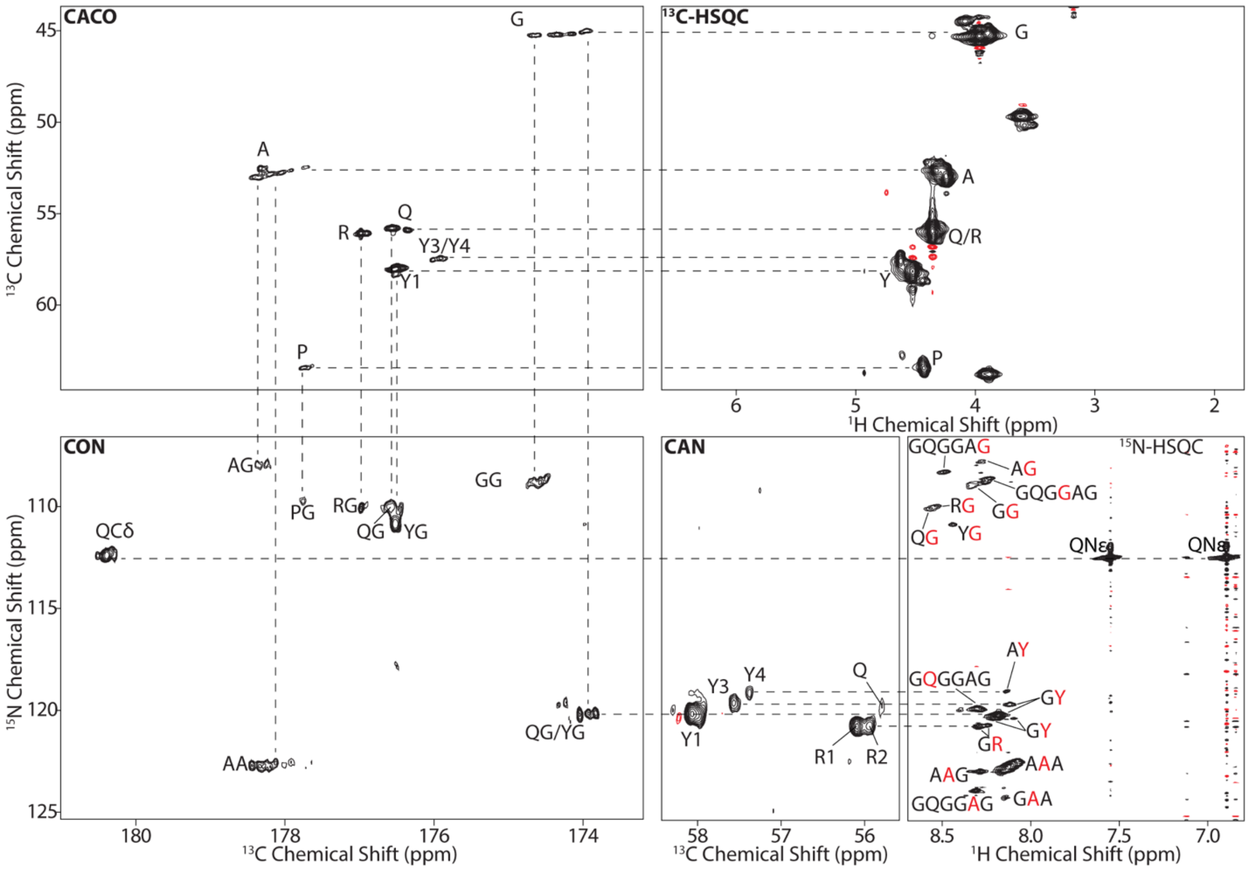

Fig. S1. X-detect experiments for condensed phase allow assignment of overlapping signal in the ^{13}C direct-detect, ^{1}H-^{13}C HSQC, and ^{1}H-^{15}N HSQC. The carbonyl dimension in the CACO allows differentiation of Arg and Gln Cα resonances, which overlap in the ^{1}H-^{13}C HSQC. The CON spectrum allows correlation of carbonyls to subsequent amides in the ^{1}H-^{15}N HSQC. CAN correlates the Cα resonances to the amide on the same residue. This experiment provides more resolution to differentiate between Tyr residues, and is the most convincing evidence that the large peak which shifts in the ^{1}H-^{15}N HSQC is Arg.

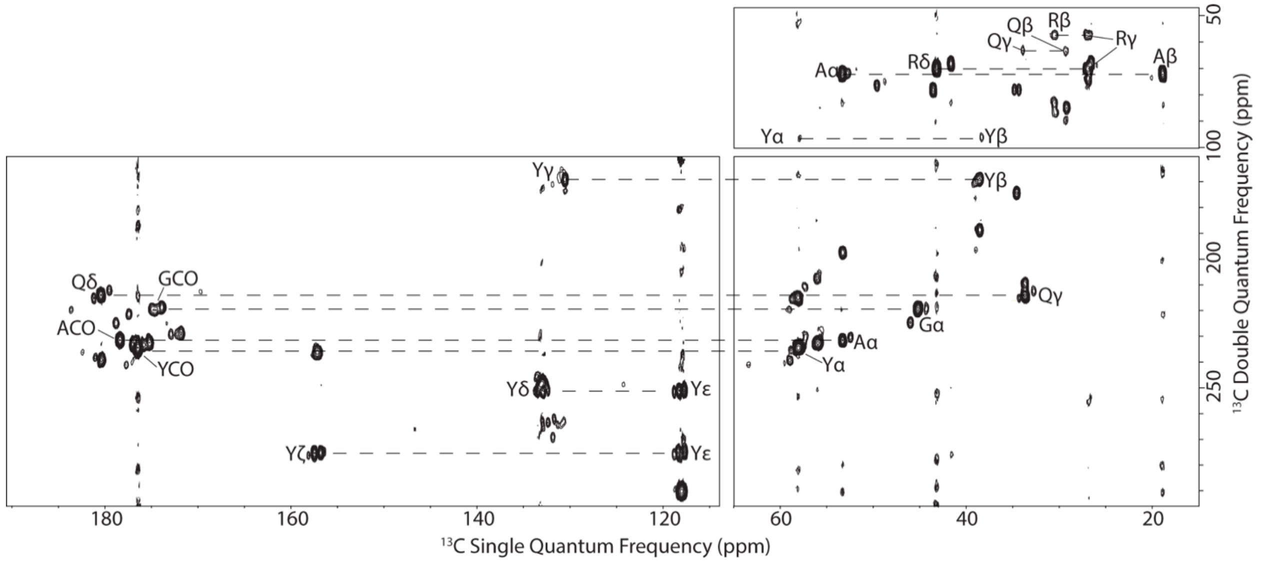

Fig. S2. ^{13}C INADEQUATE for intact glands allows assignment of directly bound carbons in each amino acid. Carbons that are directly bound to one another are correlated in the DQ dimension. Not only does this experiment confirm residue assignment of the ^{1}H-^{13}C HSQC, but it also confirms assignments of the carbonyl region from the CACO.

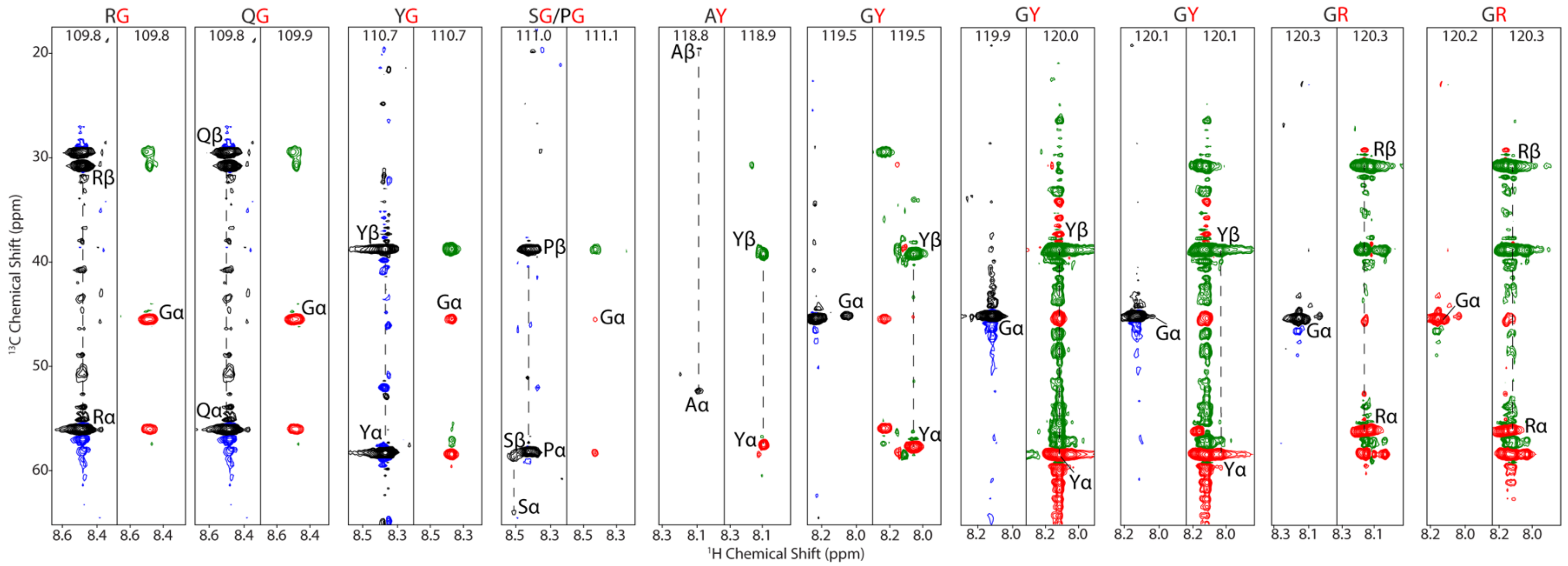

Fig. S3. CBCA(CO)NH and HNCACB for intact glands allow assignment of amide resonances in ^{1}H-^{15}N HSQC. Slices from ^{15}N dimension of the HNCACB (right, red/green) reveal Cα and Cβ peaks associated with the ^{15}N chemical shift, whereas CBCA(CO)NH (left, black/teal) reveal Cα and Cβ peaks associated with the preceding residue on the protein backbone.

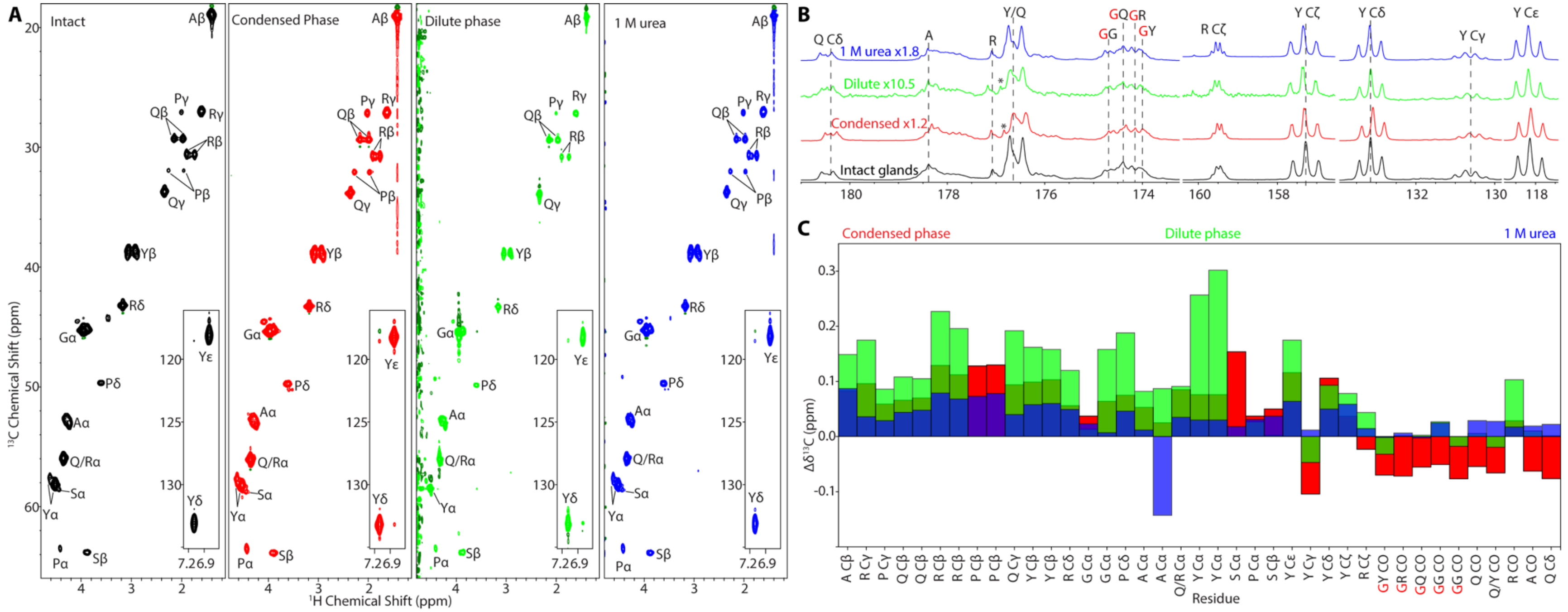

Fig. S4. ^{13}C chemical shift differences indicate the spinning dope maintains random coil structure after inducing LLPS. **(A)** ^{13}C HSQC spectra for intact glands (black), condensed phase (red), dilute phase (green) and 1 M urea (blue) and **(B)** stacked ^{13}C direct-detect spectra with largest chemical shifts indicated by dashed lines. Carbonyl peaks were assigned using X-detect experiments (**Fig. S1 and S2**). An additional unassigned peak denoted with an asterisk was visible near the Arg carbonyl peak in the direct-detect spectra following LLPS but was unobserved in both X-detect and INADEQUATE spectra. **(C)** Chemical shift differences measured from the ^{13}C direct-detect and ^{13}C HSQC spectra with respect to the intact gland sample.

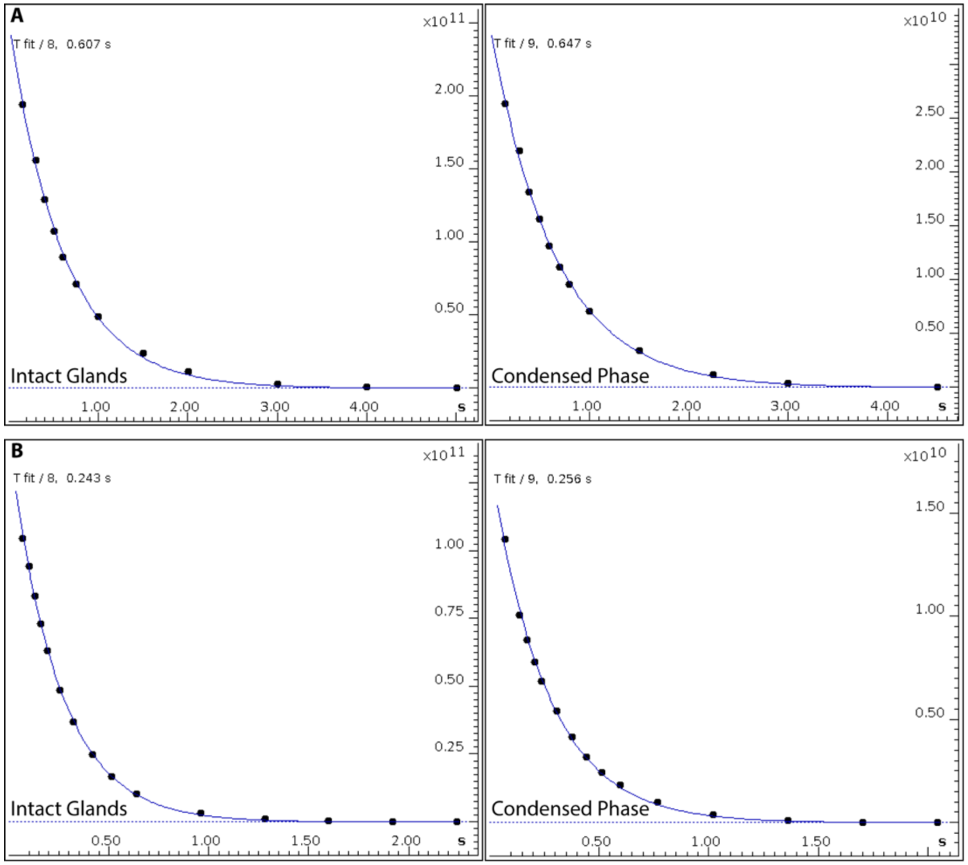

Fig. S5. Representative fits for ^{15}N T_1 and T_2 relaxation measurements show similarities in relaxation rates in both intact and condensed phase samples. Both T_1 (A) and T_2 (B) fits are shown for the most intense Tyr amide resonance at 120 ppm in the ^{1}H-^{15}N HSQC.

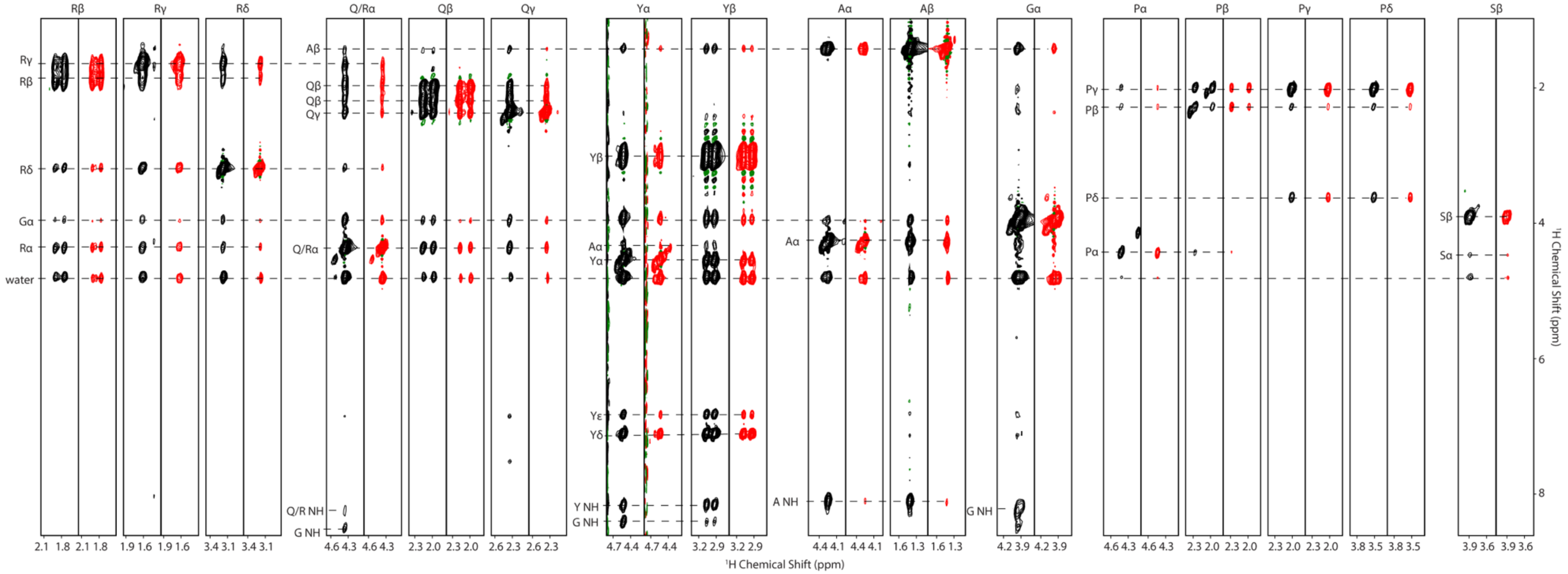

Fig. S6. ^{13}C NOESY-HSQC collected on intact glands (black) and condensed phase (red) with a 500 ms mixing time. NH correlations to backbone carbons are absent in the condensed phase. The specific ^{13}C plane for each 2D ^{1}H-^{1}H NOESY is indicated at the top of each spectrum.

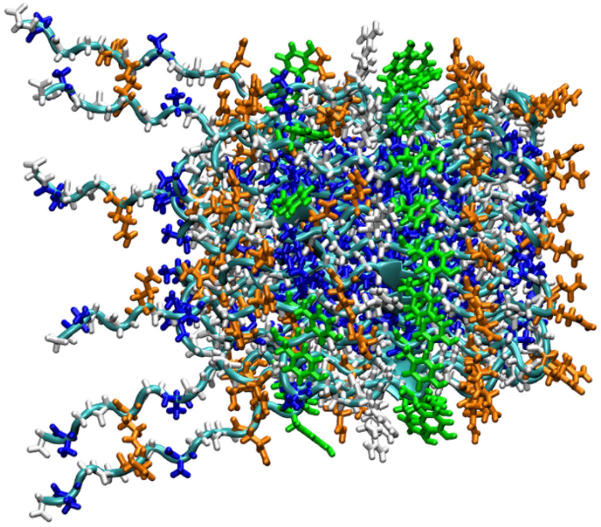

Fig. S7. Initial conformation of the hexamer peptide as generated by ColabFold.

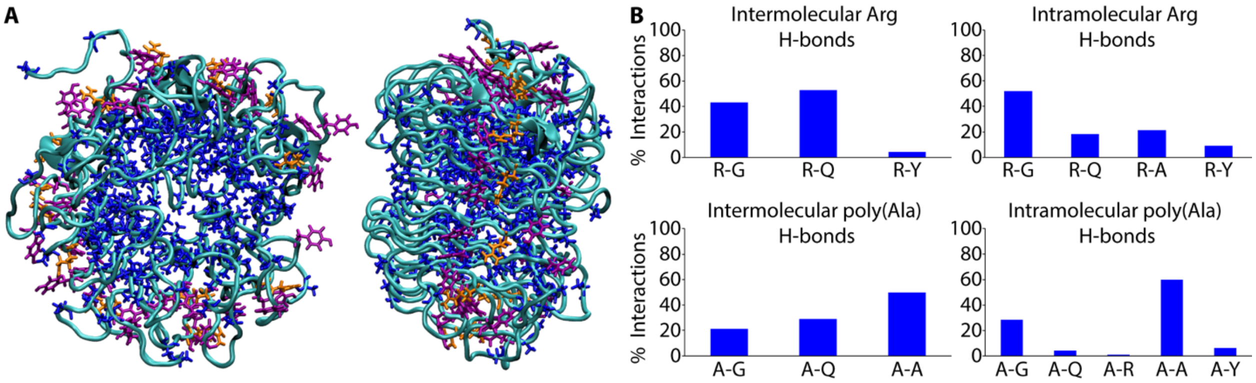

Fig. S8. Molecular dynamics (MD) simulation of six fragments from MaSp1 run with urea as the solvent.

(A) Two views of the structures in the presence of urea. **(B)** Percentage of residues which form intermolecular (left) and intramolecular (right) hydrogen bonds with Arg (top) and poly(Ala) (bottom).

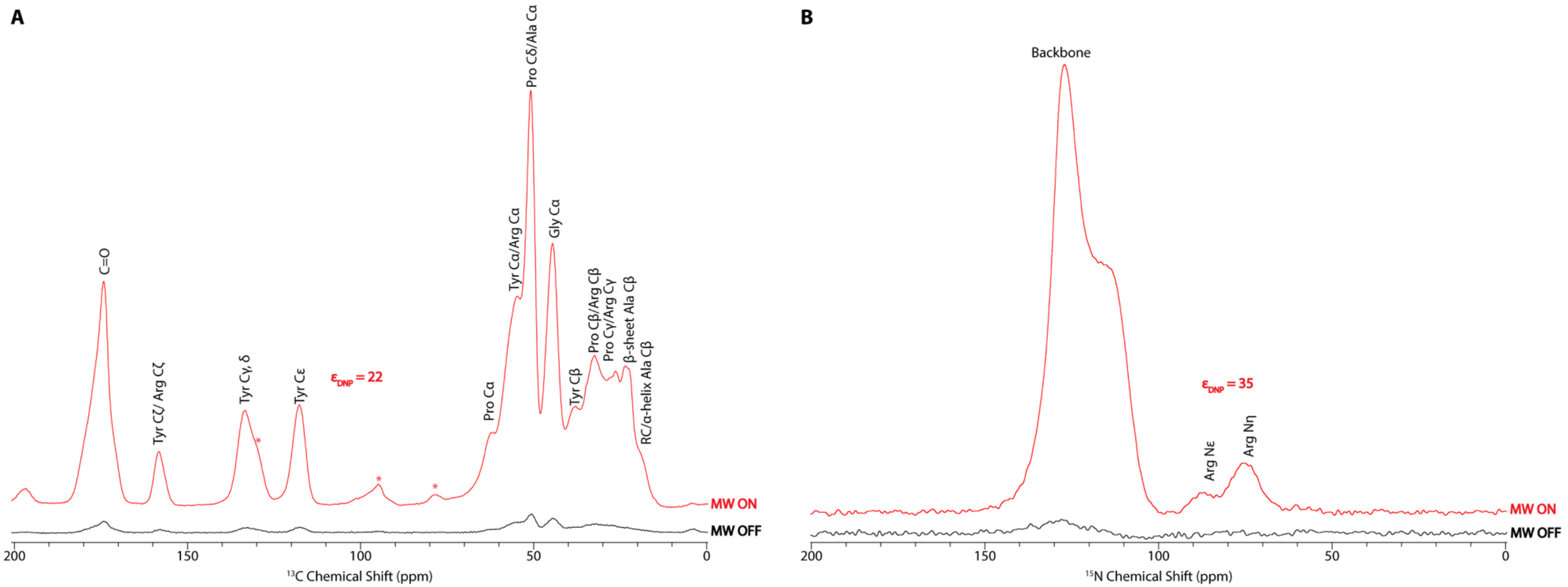

Fig. S9. DNP enhancement in the (A) ^{13}C CP-MAS and (B) ^{15}N CP-MAS spectrum of *L. hesperus* dragline silk where the spider was fed $^{13}C/^{15}N$-labeled Phe, Ala, and Arg**.** Observed DNP enhancements (ε_{DNP}) are 22 and 35 for ^{13}C and ^{15}N spectra, respectively. Asterisks (*) indicates spinning sidebands. MW on and off indicate whether microwave irradiation was turned on/off.

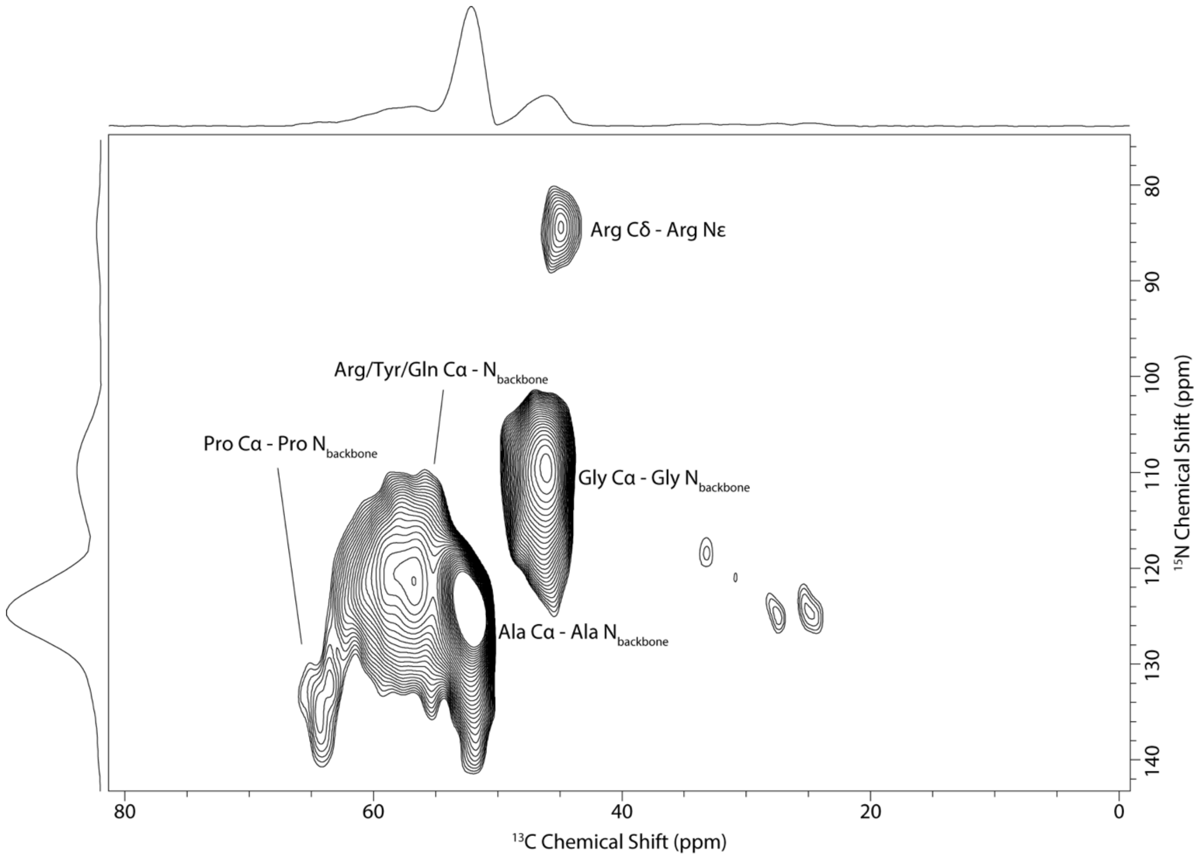

Fig. S10. 2D DNP-MAS ^{15}N-^{13}C DCP NCa spectrum of *L. hesperus* dragline silk where the spider was fed ^{13}C/^{15}N-labeled Phe, Ala, and Arg and Arg with a 4 ms DCP contact time.

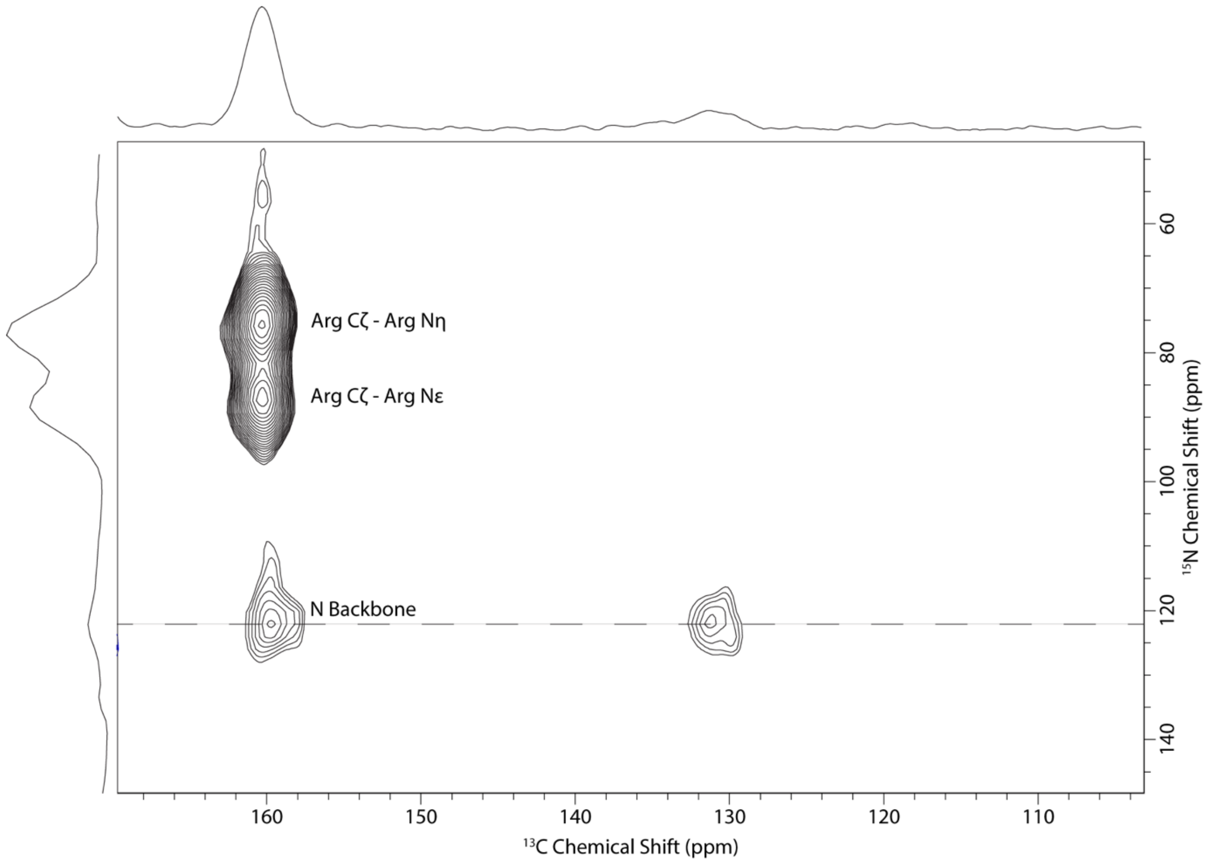

Fig S11. 2D DNP-MAS ^{15}N-^{13}C TEDOR spectrum of *L. hesperus* dragline silk where the spider was fed ^{13}C/^{15}N-labeled Phe, Ala, and Arg with a 4.2 ms mixing time.

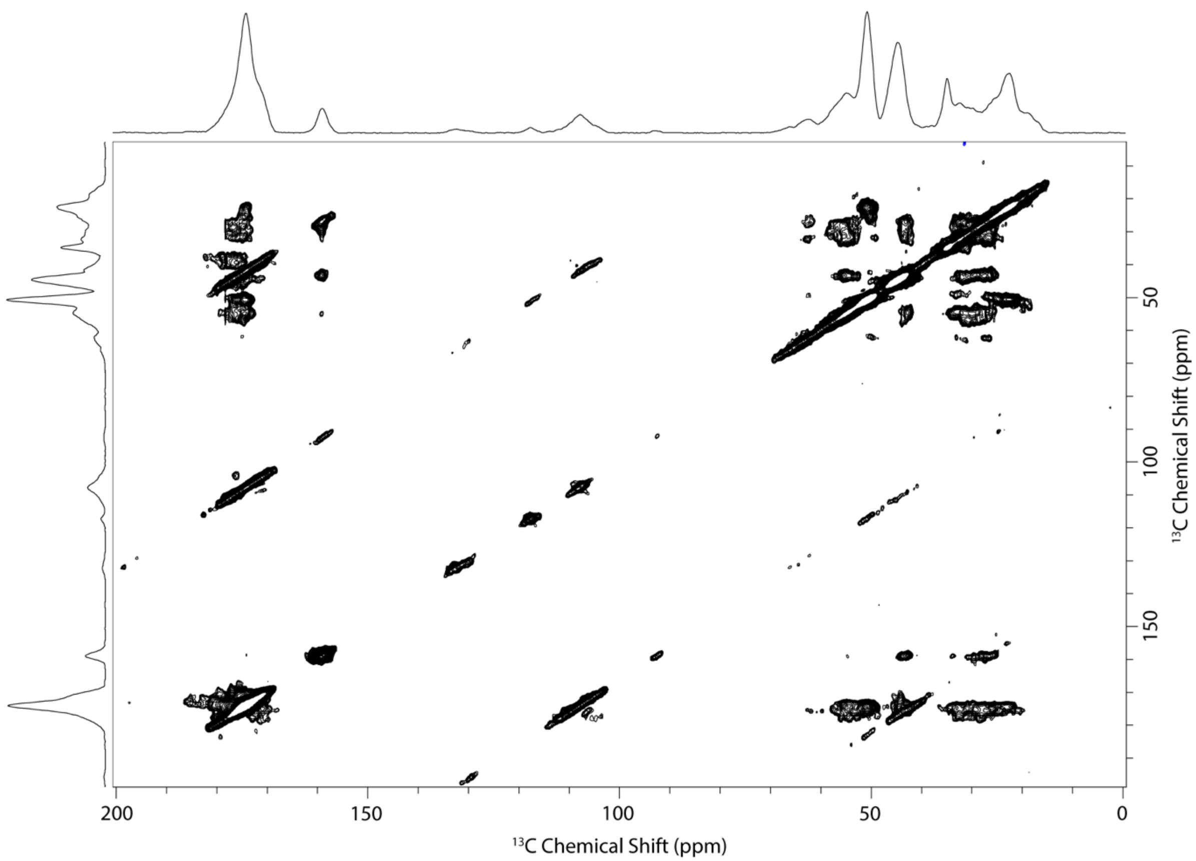

Fig. S12. 2D ^{13}C-^{13}C DARR SSNMR spectrum of *L. hesperus* dragline silk where the spider was fed $^{13}C/^{15}N$-labeled Arg and unenriched Ala, Pro, and Gly with a 100 ms mixing time at room temperature and 600 MHz.

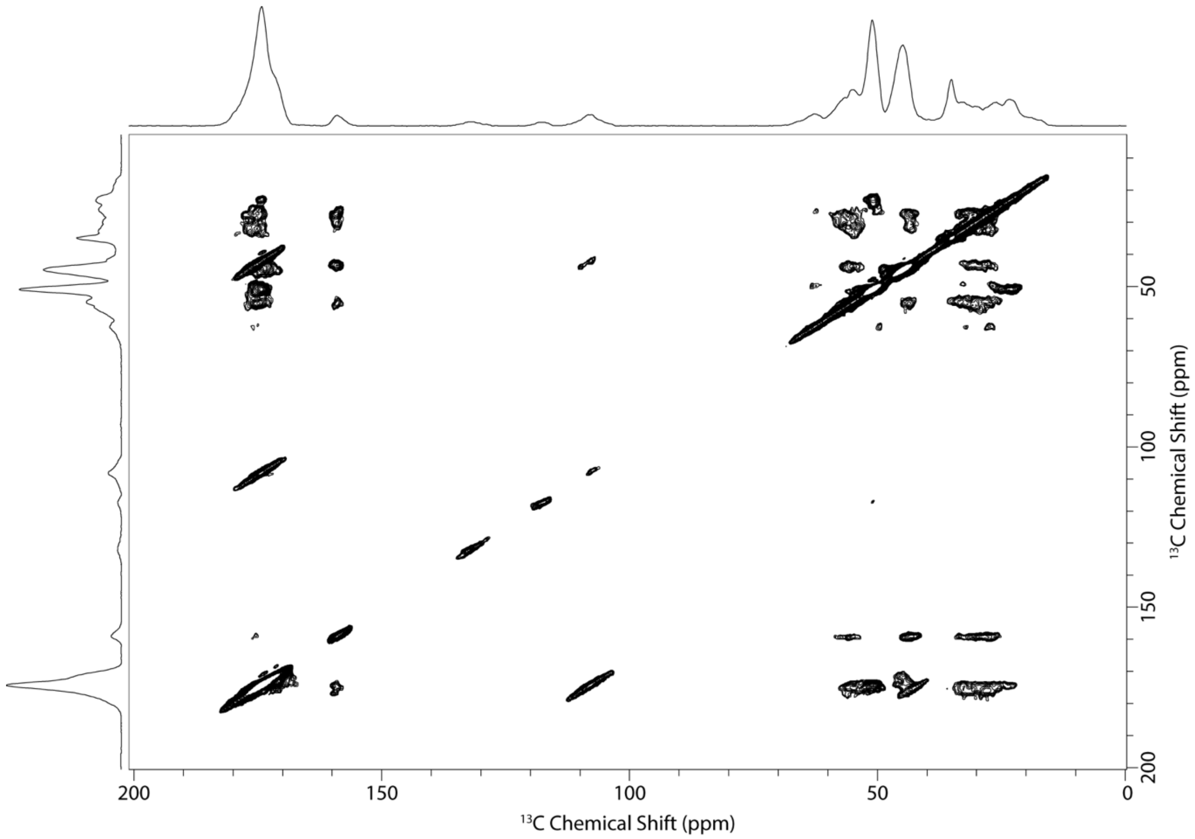

Fig. S13. 2D ^{13}C-^{13}C DARR SSNMR spectrum of *L. hesperus* dragline silk where the spider was fed ^{13}C/^{15}N-labeled Arg and unenriched Ala, Pro, and Gly with a 500 ms mixing time at room temperature and 600 MHz.

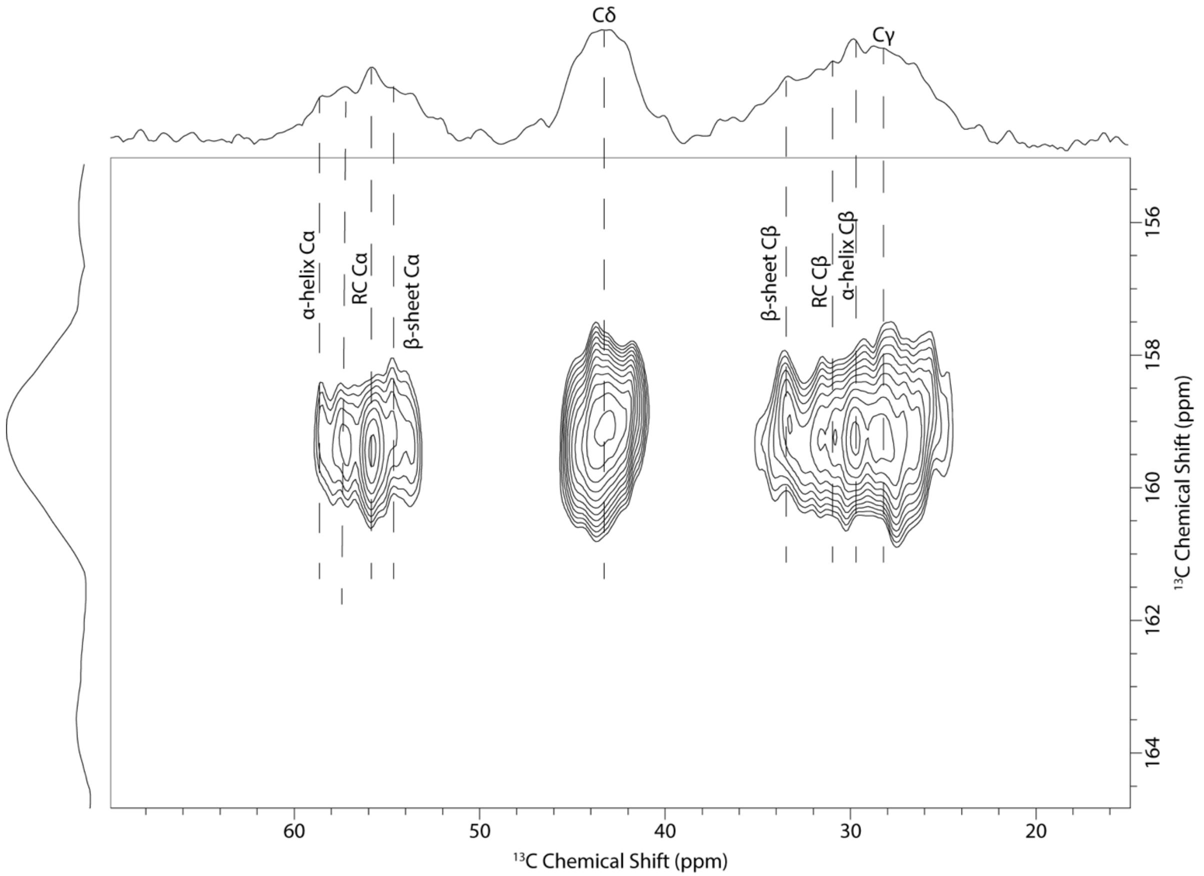

Fig. S14. Blow up of the Arg Ca and Cb sites taken from Fig. S13 when slicing through 159.0 ppm corresponding to Arg Cz. Dashed lines indicate literature chemical shifts for each secondary structure.(33) The projections of labeled Arg residues in spider silk fibers show significant β-sheet components for Cα and Cβ, consistent with Arg partial incorporation into structured regions. Additional populations at random coil (RC) chemical shift positions support the presence of interfacial or partially disordered Arg residues. α-helical content is low to negligible. Arg chemical shifts determined from this experiment are presented in **Tables S13**, **S14**, and **S17**.

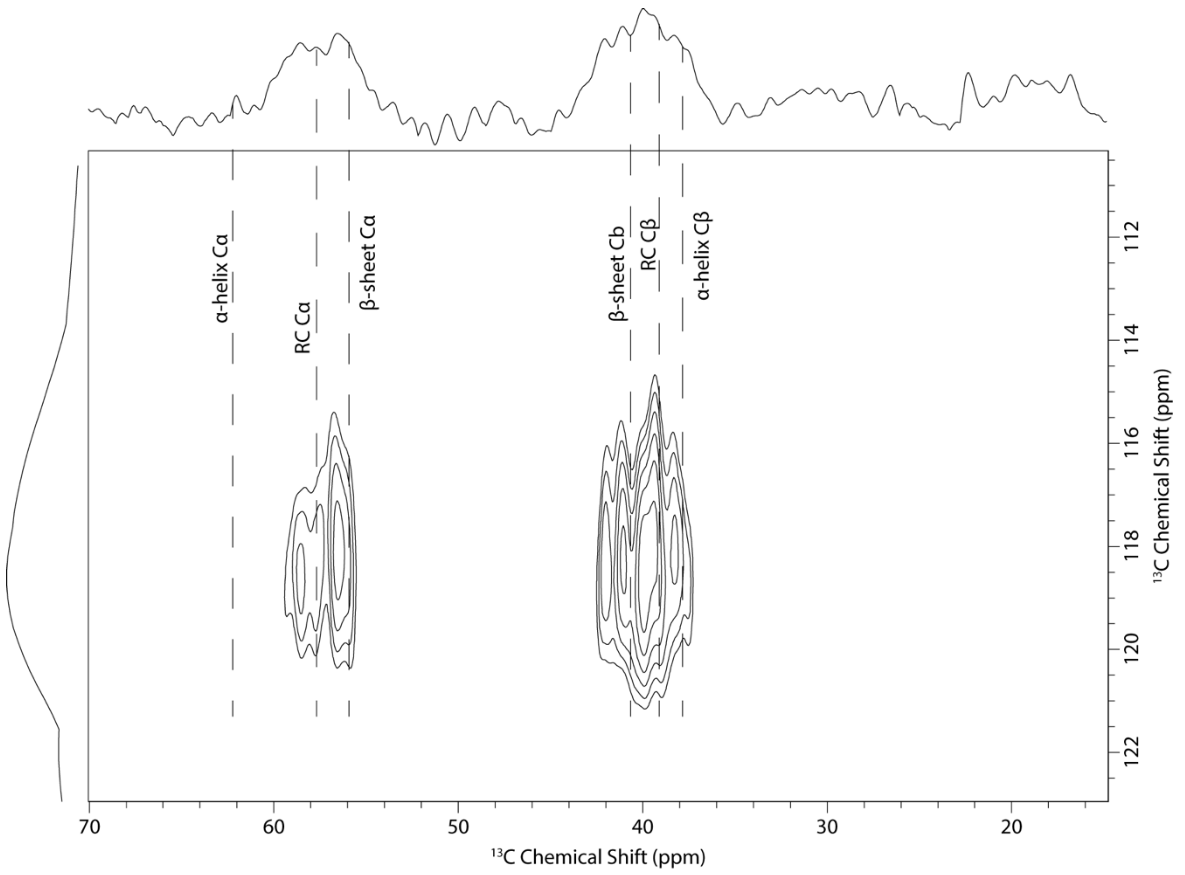

Fig. S15. Blow up of the Ca and Cb sites taken from the 2D DARR SSNMR spectrum of *L. hesperus* dragline silk where the spider was fed $^{13}C/^{15}N$-labeled Phe, Ala, and Arg when slicing through 133.3 ppm corresponding to Tyr $C\gamma,\delta$. Dashed lines indicate literature chemical shifts for each secondary structure.(33) While the central resonances align with the Tyr random coil (RC) region, β-sheet components are present with α-helix negligible.

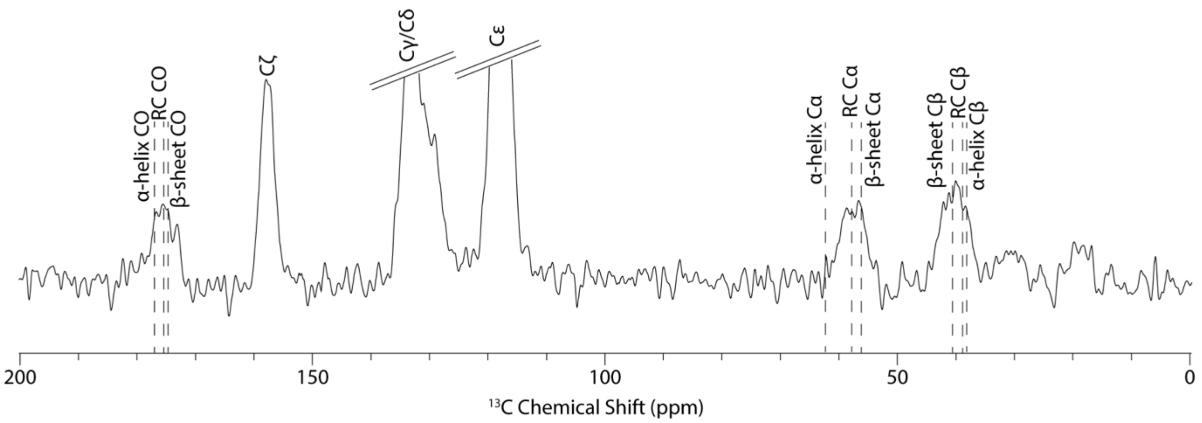

Fig. S16. The full projection from **Fig. S15**. Tyr chemical shifts determined from this experiment are presented in **Tables S13**, **S14**, and **S17**.

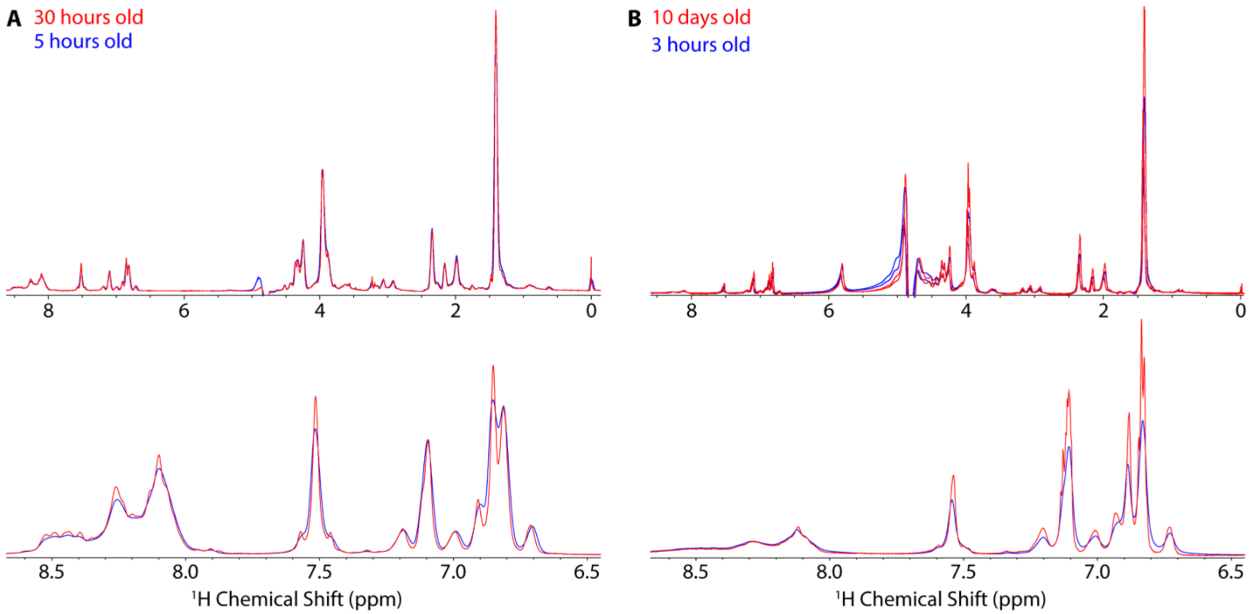

Fig. S17. Water suppressed proton spectra collected over time provides a measure of sample stability. **(A)** Intact glands show sharpening in the amide region over time, though chemical shifts are preserved. **(B)** The condensed phase is stable over several days, with some sharpening observed in the Tyr and Gln side chain residues. Intact gland NMR data was collected within 48 hrs of sample prep and condensed phase data was collected within one week.

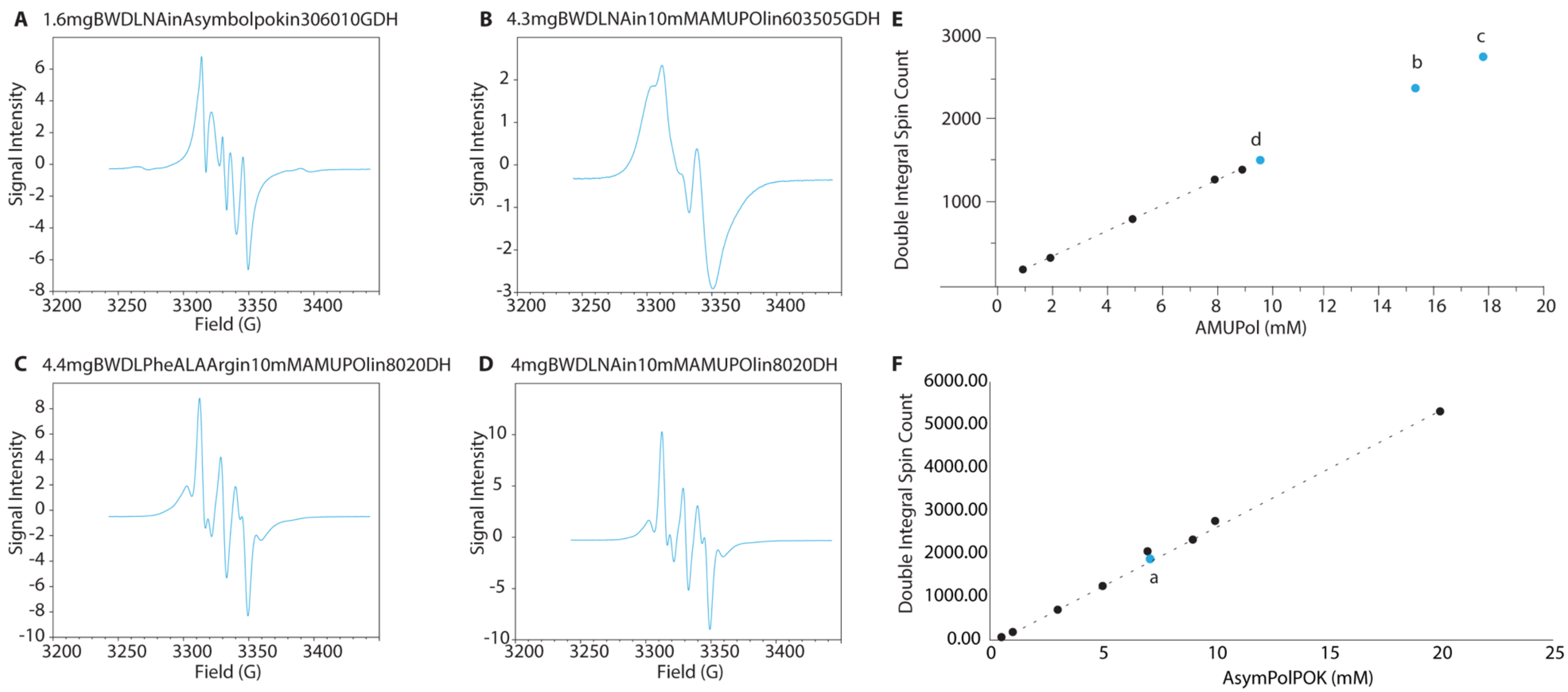

Fig. S18. (A-D) EPR experiments with different *L. hesperus* dragline silks in different radical/solvent concentrations. **(E-F)** Double integration calculations with the integrated intensity compared to a standard curve prepared with rotors containing known concentrations of AMUPol/AsymPolPOK to determine the final AMUPol concentration of 19 mM in the rotor with the sample used in the main text **(C)**.

Table S1. Chemical shifts (ppm) from ^{15}N direct-detect and ^{1}H-^{15}N HSQC solution NMR spectra for silk samples**.** The chemical shift difference (Δ) with respect to the intact gland sample is reported and literature secondary structure chemical shifts are also shown.(33)

**Residue**	**Intact**	**Condensed**	**1 M urea**	**ΔCondensed**	**Δ1 M urea**	**β–strand**	**Random coil**	**α–helix**
P	134.41	134.41	134.52	0.00	0.11			
**A**	124.18	124.23	124.13	0.05	-0.05			
G**A**A	123.98	124.02	123.98	0.04	0.00			
GQGG**A**G	123.70	123.75	123.74	0.05	0.04	125.57	123.52	121.65
A**A**G	122.78	122.81	122.94	0.03	0.16			
A**A**A	122.57	122.65	122.83	0.08	0.26			
**R**	120.36	120.63	120.43	0.27	0.07	122.60	120.59	118.99
**R**		120.57		0.21				
G**Y**	120.04	120.09	120.09	0.05	0.05			
G**Y**	120.09	120.23	120.28	0.13	0.18	122.55	120.05	119.67
G**Y**	119.51	119.57	119.58	0.06	0.07			
A**Y**	118.92	118.93	119.12	0.00	0.20			
G**Q**G	119.80	119.83	-	0.03	-	123.14	119.73	118.59
G**Q**GGAG	119.69	119.76	119.68	0.07	-0.01			
**S**	115.76	116.02	115.73	0.26	-0.03	117.44	115.94	114.78
QNε	112.28	112.35	112.23	0.07	-0.05			
Y**G**	110.75	110.79	110.80	0.04	0.05			
Q**G**	109.88	109.99	109.93	0.11	0.05			
R**G**	109.79	109.86	109.86	0.07	0.07			
G**G**	108.73	108.88	108.76	0.15	0.03			
G**G**	108.60	108.70	108.63	0.10	0.03	110.19	109.94	107.34
GQG**G**AG	108.41	108.51	108.45	0.10	0.04			
GQGGA**G**	108.10	108.20	108.14	0.10	0.04			
A**G**	107.64	107.74	107.74	0.10	0.10			
A**G**	107.57	107.64	107.68	0.07	0.11			
RNε	84.61	84.65	84.69	0.04	0.08			
RNη	71.4	71.6	71.3	0.20	-0.03			

Table S2. Proton chemical shifts (ppm) from ^1H-^{15}N HSQC solution NMR spectra for silk samples. The chemical shift difference (Δ) with respect to the intact gland sample is reported and literature secondary structure chemical shifts are also shown.(33)

<table>
<tr><th>Residue</th><th>Intact</th><th>Condensed</th><th>1 M urea</th><th>ΔCondensed</th><th>Δ1 M urea</th><th>β-strand</th><th>Random coil</th><th>α-helix</th></tr>
<tr><td>GAA</td><td>8.09</td><td>8.13</td><td></td><td>0.04</td><td></td><td rowspan="4">8.59</td><td rowspan="4">8.11</td><td rowspan="4">7.99</td></tr>
<tr><td>GQGGAG</td><td>8.26</td><td>8.30</td><td>8.27</td><td>0.04</td><td>0.01</td></tr>
<tr><td>AAG</td><td>8.24</td><td>8.27</td><td>8.25</td><td>0.04</td><td>0.01</td></tr>
<tr><td>AAA</td><td>8.07</td><td>8.11</td><td>8.11</td><td>0.04</td><td>0.04</td></tr>
<tr><td>R</td><td rowspan="2">8.17</td><td>8.28</td><td></td><td>0.11</td><td></td><td rowspan="2">8.57</td><td rowspan="2">8.17</td><td rowspan="2">8.03</td></tr>
<tr><td>R</td><td>8.23</td><td></td><td>0.06</td><td></td></tr>
<tr><td>GY</td><td>8.16</td><td>8.20</td><td>8.14</td><td>0.04</td><td>-0.02</td><td rowspan="4">8.69</td><td rowspan="4">7.90</td><td rowspan="4">8.10</td></tr>
<tr><td>GY</td><td>8.13</td><td>8.17</td><td>8.07</td><td>0.04</td><td>-0.07</td></tr>
<tr><td>GY</td><td>8.05</td><td>8.08</td><td>8.09</td><td>0.03</td><td>0.04</td></tr>
<tr><td>AY</td><td>8.07</td><td>8.11</td><td>8.11</td><td>0.04</td><td>0.04</td></tr>
<tr><td>GQG</td><td>8.34</td><td>8.39</td><td></td><td>0.04</td><td></td><td rowspan="2">8.51</td><td rowspan="2">8.25</td><td rowspan="2">8.11</td></tr>
<tr><td>GQGGAG</td><td>8.24</td><td>8.29</td><td>8.25</td><td>0.05</td><td>0.01</td></tr>
<tr><td>QNε</td><td>7.50</td><td>7.54</td><td>7.50</td><td>0.04</td><td>0.01</td><td></td><td></td><td></td></tr>
<tr><td>QNε</td><td>6.84</td><td>6.88</td><td>6.85</td><td>0.04</td><td>0.01</td><td></td><td></td><td></td></tr>
<tr><td>YG</td><td>8.38</td><td>8.43</td><td></td><td>0.05</td><td></td><td rowspan="8">8.27</td><td rowspan="8">8.34</td><td rowspan="8">8.23</td></tr>
<tr><td>QG</td><td>8.50</td><td>8.55</td><td></td><td>0.05</td><td></td></tr>
<tr><td>RG</td><td>8.48</td><td>8.52</td><td></td><td>0.04</td><td></td></tr>
<tr><td>GG</td><td>8.25</td><td>8.32</td><td></td><td>0.07</td><td></td></tr>
<tr><td>GG</td><td>8.19</td><td>8.24</td><td>8.21</td><td>0.05</td><td>0.02</td></tr>
<tr><td>GQGGAG</td><td>8.17</td><td>8.22</td><td>8.18</td><td>0.05</td><td>0.01</td></tr>
<tr><td>GQGGAG</td><td>8.42</td><td>8.47</td><td></td><td>0.05</td><td></td></tr>
<tr><td>AG</td><td>8.22</td><td>8.26</td><td>8.26</td><td>0.04</td><td>0.04</td></tr>
</table>

Table S3. Carbon chemical shifts (ppm) from ^{13}C direct-detect and ^{1}H-^{13}C HSQC solution NMR spectra for silk samples. The chemical shift difference (Δ) with respect to the intact gland sample is reported and literature secondary structure chemical shifts are shown.(33)

Residue	Intact	Condensed	Dilute	1 M urea	ΔCondensed	ΔDilute	Δ1 M urea	β-strand	Random coil	α-helix
**A Cβ**	18.89	18.97	19.04	18.98	0.08	0.15	0.09	21.72	19.03	18.27
**R Cγ**	26.99	27.09	27.16	27.03	0.10	0.18	0.04			
**P Cγ**	27.07	27.13	27.16	27.10	0.06	0.09	0.03			
**Q Cβ**	29.28	29.34	29.38	29.32	0.07	0.11	0.04	31.92	28.67	28.33
**Q Cβ**	29.28	29.35	29.38	29.33	0.07	0.11	0.05			
**R Cβ**	30.56	30.68	30.78	30.63	0.13	0.23	0.08	32.36	30.53	30.00
**R Cβ**	30.59	30.70	30.79	30.66	0.11	0.20	0.07			
**P Cβ**	31.93	32.06	-	32.01	0.13		0.07	32.45	31.87	31.08
**P Cβ**	31.94	32.07	-	32.02	0.13		0.08			
**Q Cγ**	33.70	33.80	33.90	33.74	0.09	0.19	0.04			
**Y Cβ**	38.63	38.73	38.80	38.69	0.10	0.16	0.06	40.79	38.71	38.38
**Y Cβ**	38.64	38.74	38.79	38.70	0.10	0.16	0.06			
**R Cδ**	43.20	43.25	43.32	43.25	0.06	0.12	0.05			
**G Cα**	44.53	44.57	44.54	44.55	0.04	0.01	0.02	45.08	45.34	47.02
**G Cα**	45.29	45.35	45.44	45.29	0.06	0.16	0.01			
**P Cδ**	49.67	49.74	49.86	49.71	0.07	0.19	0.05			
**A Cα**	52.67	52.72	52.75	52.68	0.05	0.08	0.01	50.86	52.67	54.86
**A Cα**	53.00	53.03	53.09	52.86	0.03	0.09	-0.14			
**Q/R Cα**	55.95	56.03	56.04	55.98	0.09	0.09	0.03	54.33/54.63	55.94/55.96	58.61/59.05
**Y Cα**	57.58	57.66	57.84	57.61	0.08	0.26	0.03	56.56	57.72	61.07
**Y Cα**	58.15	58.22	58.45	58.18	0.08	0.30	0.03			
**S Cα**	58.58	58.73	-	58.60	0.15		0.02	57.14	58.35	60.86
**P Cα**	63.49	63.53	63.52	63.52	0.04	0.03	0.03	62.79	63.53	65.52
**S Cβ**	63.82	63.87	63.82	63.86	0.05	0.00	0.04	65.39	63.88	62.81
**Y Cε**	118.07	118.19	118.25	118.14	0.12	0.17	0.06			

**Y Cγ**	130.75	130.64	130.70	130.76	-0.10	-0.05	0.01			
**Y Cδ**	133.05	133.16	133.15	133.10	0.11	0.09	0.05			
**Y Cζ**	157.27	157.31	157.35	157.33	0.04	0.08	0.06			
**R Cζ**	159.49	159.47	159.53	159.51	-0.02	0.04	0.01			
**G**Y **CO**	174.07	174.00	174.04	174.07	-0.07	-0.03	0.00			
**G**R **CO**	174.21	174.14	174.22	174.22	-0.07	0.00	0.01			
**G**Q **CO**	174.39	174.34	174.39	174.39	-0.06	0.00	0.00	173.01	174.30	176.31
**G**G **CO**	174.63	174.58	174.66	174.66	-0.05	0.03	0.02			
G**G CO**	174.76	174.69	174.75	174.76	-0.08	-0.02	0.00			
**Q CO**	176.45	176.39	176.45	176.48	-0.05	0.01	0.03	174.58	175.88	178.35
**Q/Y CO**	176.72	176.65	176.70	176.74	-0.07	-0.02	0.03	174.58/174.65	175.88/175.32	178.35/177.05
**R CO**	177.07	177.10	177.17	177.09	0.03	0.10	0.02	175.04	175.91	178.11
**A CO**	178.38	178.32	178.39	178.40	-0.06	0.01	0.02	175.30	177.39	179.58
**Q Cδ**	180.47	180.39	180.47	180.49	-0.08	0.00	0.02			

Table S4. Proton chemical shifts (ppm) from ^1H-^{13}C HSQC spectra for silk samples. The chemical shift difference (Δ) with respect to the intact gland sample is reported and literature random coil chemical shifts are shown.(34)

Residue	Intact	Condensed	Dilute	1 M urea	ΔCondensed	ΔDilute	Δ1 M urea	Random coil
A Hβ	1.40	1.42	1.39	1.40	0.01	-0.01	-0.01	1.39
R Hγ	1.60	1.62	1.60	1.60	0.01	0.00	0.00	
P Hγ	2.01	2.02	2.00	2.02	0.01	-0.01	0.01	
Q Hβ1	2.15	2.17	2.14	2.15	0.01	-0.01	0.00	2.12
Q Hβ2	1.98	1.99	1.97	1.98	0.01	-0.01	0.00	1.99
R Hβ1	1.90	1.91	1.89	1.90	0.01	-0.01	0.00	1.86
R Hβ2	1.74	1.76	1.74	1.75	0.02	0.00	0.00	1.76
P Hβ1	2.26	2.28		2.27	0.02		0.01	2.29
P Hβ2	1.96	1.98		1.97	0.01		0.01	1.94
Q Hγ	2.34	2.35	2.33	2.34	0.02	-0.01	0.00	
Y Hβ1	3.06	3.07	3.04	3.06	0.01	-0.01	0.01	3.03
Y Hβ2	2.92	2.93	2.91	2.93	0.01	-0.01	0.01	2.98
R Hδ	3.17	3.18	3.16	3.18	0.01	-0.01	0.01	
G Hα	4.07	4.09	4.07	4.08	0.02	-0.01	0.01	3.96
G Hα	3.95	3.97	3.95	3.96	0.02	0.00	0.01	
P Hδ	3.62	3.63	3.60	3.62	0.01	-0.02	0.00	
A Hα	4.31	4.33	4.31	4.32	0.02	0.00	0.01	4.32
A Hα	4.24	4.25	4.23	4.25	0.01	-0.01	0.01	
Q/R Hα	4.35	4.36	4.34	4.35	0.01	-0.01	0.00	4.34
Y Hα	4.62	4.63	4.61	4.62	0.01	-0.01	0.00	4.55
Y Hα	4.51	4.53	4.51	4.52	0.02	-0.01	0.01	
S Hα	4.44	4.44		4.44	0.01		0.00	4.47
P Hα	4.42	4.43	4.42	4.42	0.02	0.00	0.01	4.42
S Hβ	3.87	3.89	3.86	3.87	0.01	-0.01	0.00	3.89/3.87
Y Hε	6.80	6.83	6.81	6.82	0.02	0.00	0.01	
Y Hδ	7.09	7.10	7.09	7.10	0.01	0.00	0.01	

Table S5. ^{15}N T_1 relaxation time (s) measured for intact glands, condensed phase, and 1 M urea samples.

**Residue**	**Intact**		**Condensed**		**1 M urea**	
	T1	Error	T1	Error	T1	Error
G**A**A	0.674	0.041	0.705	0.011	0.714	0.044
GQGG**A**G	0.682	< 0.001	0.718	0.006	0.609	0.027
A**A**G	0.684	0.001	0.744	0.005	0.732	0.016
A**A**A	0.692	< 0.001	0.717	0.001	0.775	0.016
**R**	0.621	< 0.001	0.647	0.027		
**R**			0.651	0.007		
G**Y**	0.607	< 0.001	0.647	0.001	0.681	0.003
G**Y**	0.617	0.003	0.662	0.015		
G**Y**	0.615	0.001	0.610	0.005	0.645	0.009
A**Y**	0.625	0.035	0.653	0.008	0.726	0.028
G**Q**G	0.664	0.001	0.693	0.015		
G**Q**GGAG	0.669	0.003	0.715	0.005	0.822	0.038
Y**G**	0.631	0.015	0.663	0.009		
Q**G**	0.695	0.005	0.706	0.009		
R**G**	0.679	0.001	0.716	0.010		
G**G**	0.692	0.001	0.738	0.009		
GQG**G**AG	0.693	0.005	0.721	0.005		
GQGGA**G**	0.724	0.005	0.780	0.008		
A**G**	0.734	0.002	0.805	0.012		
Average	0.667		0.702		0.713	
SD	0.038		0.049		0.068	

Table S6. ^{15}N T_2 relaxation time (s) measured for intact glands, condensed phase, and 1 M urea samples.

Residue	Intact		Condensed		1 M urea	
	T2	Error	T2	Error	T2	Error
G**A**A	0.251	0.001	0.206	0.006		
GQGG**A**G	0.315	< 0.001	0.285	0.003	0.217	0.018
A**A**G	0.235	< 0.001	0.265	0.003	0.291	0.007
A**A**A	0.260	< 0.001	0.283	0.001	0.341	0.001
**R**	0.256	< 0.001	0.273	0.003		
**R**			0.300	0.005		
G**Y**	0.243	< 0.001	0.256	0.001	0.324	0.002
G**Y**	0.226	0.001	0.227	0.004		
G**Y**	0.234	0.001	0.224	0.002	0.342	0.007
A**Y**	0.208	0.001	0.215	0.004	0.328	0.013
G**Q**G	0.318	0.001	0.318	0.006		
G**Q**GGAG	0.328	< 0.001	0.333	0.002	0.317	0.016
Y**G**	0.257	< 0.001	0.249	0.004		
Q**G**	0.338	0.003	0.268	0.004		
R**G**	0.317	< 0.001	0.273	0.005		
G**G**	0.340	< 0.001	0.242	0.004		
GQG**G**AG	0.333	< 0.001	0.234	0.003		
GQGGA**G**	0.367	< 0.001	0.216	0.003		
A**G**	0.344	0.001	0.195	0.008		
Average	0.287		0.253		0.309	
SD	0.050		0.038		0.044	

Table S7. ^{1}H-^{15}N hNOE values measured for intact glands, condensed phase, and 1 M urea samples.

Residue	Intact		Condensed		1 M urea	
	NOE	Error	NOE	Error	NOE	Error
G**A**A	0.283	0.004	0.431	0.015		
GQGG**A**G	0.140	0.001	0.120	0.009	0.083	0.062
A**A**G	0.132	0.003	0.155	0.008	-0.060	0.033
A**A**A	0.257	0.001	0.247	0.002	0.060	0.003
**R**	0.334	0.001	0.366	0.008	0.059	0.113
**R**			0.309	0.012		
G**Y**	0.381	< 0.001	0.356	0.002	0.190	0.006
G**Y**	0.436	0.008	0.449	0.019		
G**Y**	0.399	0.004	0.321	0.007	0.321	0.026
A**Y**	0.397	0.013	0.276	0.016	0.088	0.059
G**Q**G	0.251	0.002	0.183	0.013		
G**Q**GGAG	0.152	0.001	0.213	0.006	-0.034	0.045
Y**G**	0.330	0.002	0.349	0.012	0.100	0.064
Q**G**	0.113	0.001	0.141	0.009	-0.066	0.076
R**G**	0.163	0.001				
G**G**	0.141	0.002	0.206	0.010	0.109	0.088
GQG**G**AG	0.205	0.001	0.157	0.007	0.057	0.040
GQGGA**G**	0.047	0.001	0.049	0.008	-0.122	0.080
A**G**	0.054	0.004	0.061	0.014	-0.076	0.073
Average	0.234		0.240		0.051	
SD	0.124		0.123		0.118	

Table S8. DSSP analysis of the simulated 116-residue MaSp1 hexamer assembly (**Fig. 4, S8**). Assemblies display a high degree of disorder with loops and bends representing the majority of secondary structure.

Secondary Structure	Water + NaCl (Intact Gland Phase)	Water + KH_2PO_4 (Condensed Phase)	Urea (Denatured Phase)
Loops	46%	46%	47%
Bends	21%	22%	20%
Turns	5%	6%	5%
PPII Helices	13%	12%	13%
β-strands	4%	5%	3%
β-bridges	11%	10%	12%

Table S9. Comparison of predicted and experimental ^{15}N chemical shifts (ppm) for simulated 116-residue MaSp1 hexamer assemblies. The chemical shift difference (Δ) between experiment and models is shown.

Residue	Experimental			SHIFTX2						Δ		
	Intact	Condensed	1 M urea	NaCl		Phosphate		Urea		Intact	Condensed	1 M urea
				Average	SD	Average	SD	Average	SD			
G**A**A	123.98	124.02	123.98	121.77	2.39	121.60	1.94	121.64	1.69	2.21	2.42	2.34
GQGG**A**G	123.70	123.75	123.74	122.65	1.21	123.13	2.27	123.50	1.80	1.05	0.62	0.24
A**A**G	122.78	122.81	122.94	122.36	1.89	122.73	2.10	122.58	2.18	0.42	0.08	0.36
A**A**A	122.57	122.65	122.83	122.61	2.35	122.79	2.10	122.72	2.22	-0.04	-0.14	0.11
**R**	120.36	120.63	120.43	119.35	2.50	120.46	3.37	120.43	2.42	1.01	0.17	0.00
**R**		120.57									0.11	
G**Y**	120.04	120.09	120.09							0.65	1.19	0.55
G**Y**	120.09	120.23	120.28	119.39	2.56	118.90	1.77	119.54	2.75	0.70	1.33	0.74
G**Y**	119.51	119.57	119.58							0.12	0.67	0.03
G**Q**G	119.80	119.83	119.68	118.60	1.65	119.79	2.14	119.80	1.99	1.20	0.04	-0.13
G**Q**GGAG	119.69	119.76		118.93	2.26	119.40	2.08	119.18	2.40	0.75	0.36	0.49
Y**G**	110.75	110.79	110.80	111.22	2.46	111.63	2.45	111.48	2.12	-0.47	-0.84	-0.68
Q**G**	109.88	109.99	109.93	109.86	2.15	110.05	2.44	109.90	2.55	0.02	-0.06	0.03
R**G**	109.79	109.86	109.86	110.83	2.73	110.26	2.50	109.76	2.44	-1.04	-0.40	0.10
G**G**	108.73	108.88	108.76	107.59	1.35	107.56	1.57	107.75	1.58	1.14	1.32	1.01
G**G**	108.60	108.70	108.63	107.59	1.35	107.56	1.57	107.75	1.58	1.01	1.14	0.88
GQG**G**AG	108.41	108.51	108.45	107.73	1.03	108.02	1.30	108.45	1.47	0.68	0.49	0.00
GQGGA**G**	108.10	108.20	108.14	107.67	1.72	108.18	1.31	108.72	1.70	0.43	0.02	-0.58
A**G**	107.64	107.74	107.74	108.19	2.28	107.55	2.18	108.38	1.88	-0.55	0.19	-0.64
A**G**	107.57	107.64	107.68							-0.62	0.09	-0.70

Table S10. Comparison of predicted and experimental NH chemical shifts (ppm) for simulated 116-residue MaSp1 hexamer assemblies. The chemical shift difference (Δ) between experiment and models is shown.

Residue	Experimental			SHIFTX2						Δ		
	Intact	Condensed	1 M urea	NaCl		Phosphate		Urea		Intact	Condensed	1 M urea
				Average	SD	Average	SD	Average	SD			
G**A**A	8.09	8.13		8.31	0.47	8.12	0.48	8.17	0.44	-0.22	0.01	
GQGG**A**G	8.26	8.30	8.27	8.30	0.30	8.26	0.25	8.24	0.18	-0.04	0.04	0.03
A**A**G	8.24	8.27	8.25	8.17	0.35	8.14	0.34	8.15	0.29	0.07	0.13	0.10
A**A**A	8.07	8.11	8.11	8.15	0.43	8.25	0.34	8.23	0.35	-0.08	-0.14	-0.12
**R**	8.17	8.28		8.49	0.52	8.27	0.34	8.21	0.42	-0.32	0.01	
G**Y**	8.16	8.20	8.14							-0.10	-0.06	-0.19
G**Y**	8.13	8.17	8.07	8.26	0.50	8.26	0.51	8.33	0.48	-0.13	-0.09	-0.27
G**Y**	8.05	8.08	8.09							-0.21	-0.18	-0.24
G**Q**G	8.34	8.39		8.21	0.33	8.28	0.33	8.31	0.34	0.13	0.11	
G**Q**GGAG	8.24	8.29	8.25	8.30	0.27	8.24	0.26	8.31	0.24	-0.06	0.05	-0.06
QNε	7.50	7.54	7.50	7.26	0.17	7.30	0.15	7.30	0.15	0.24	0.24	0.20
QNε	6.84	6.88	6.85	6.98	0.12	6.99	0.12	7.00	0.13	-0.14	-0.11	-0.15
Y**G**	8.38	8.43		8.46	0.44	8.45	0.58	8.41	0.48	-0.08	-0.02	
Q**G**	8.50	8.55		8.28	0.41	8.34	0.39	8.27	0.38	0.22	0.21	
R**G**	8.48	8.52		8.36	0.23	8.38	0.59	8.38	0.36	0.12	0.14	
G**G**	8.25	8.32		8.16	0.42	8.28	0.37	8.23	0.42	0.09	0.04	
G**G**	8.19	8.24	8.21							0.03	-0.04	-0.02
GQG**G**AG	8.17	8.22	8.18	8.22	0.28	8.23	0.40	8.29	0.17	-0.05	-0.01	-0.11
GQGGA**G**	8.42	8.47		8.23	0.34	8.33	0.34	8.34	0.22	0.19	0.14	
A**G**	8.22	8.26	8.26	8.25	0.37	8.32	0.24	8.42	0.29	-0.03	-0.06	-0.16

Table S11. Comparison of predicted and experimental ^{13}C chemical shifts (ppm) for simulated 116-residue MaSp1 hexamer assemblies. The chemical shift difference (Δ) between experiment and models is shown

Residue	Experimental				SHIFTX2						Δ		
	Intact	Condensed	Dilute	1 M urea	NaCl		Phosphate		Urea		Intact	Condensed	1 M urea
					Average	SD	Average	SD	Average	SD			
**A Cβ**	18.89	18.97	19.04	18.98	20.22	1.30	20.19	1.22	20.47	1.30	-1.33	-1.22	-1.49
**R Cγ**	26.99	27.09	27.16	27.03	26.89	0.38	27.18	0.32	27.08	0.25	0.10	-0.09	-0.05
**Q Cβ***	29.28	29.34	29.38	29.32	29.73	1.30	29.82	1.38	29.76	1.35	-0.45	-0.48	-0.44
**R Cβ***	30.57	30.69	30.78	30.65	32.06	0.97	31.39	1.75	32.02	1.47	-1.49	-0.70	-1.37
**Q Cγ**	33.70	33.80	33.90	33.74	33.72	0.32	33.77	0.24	33.82	0.26	-0.02	0.03	-0.08
**Y Cβ***	38.64	38.74	38.80	38.69	40.05	1.32	40.09	1.49	39.80	1.36	-1.41	-1.35	-1.11
**R Cδ**	43.20	43.25	43.32	43.25	43.18	0.26	43.07	0.18	43.10	0.31	0.02	0.18	0.15
**G Cα**	44.53	44.57	44.54	44.55	45.11	0.57	45.12	0.59	45.14	0.59	-0.58	-0.55	-0.59
**G Cα**	45.29	45.35	45.44	45.29							0.18	0.23	0.15
**A Cα**	52.67	52.72	52.75	52.68	52.01	0.99	51.92	0.95	51.79	0.92	0.66	0.80	0.89
**A Cα**	53.00	53.03	53.09	52.86							0.99	1.11	1.07
**Q Cα**	55.95	56.03	56.04	55.98	55.22	1.03	55.24	1.12	55.36	1.18	0.73	0.79	0.62
**R Cα**					54.95	1.05	55.26	1.38	55.23	1.22	1.00	0.77	-55.23
**Y Cα**	57.58	57.66	57.84	57.61	57.34	1.27	57.32	0.96	57.31	1.07	0.24	0.34	0.30
**Y Cα**	58.15	58.22	58.45	58.18							0.81	0.90	0.87
**Y Cε**	118.07	118.19	118.25	118.14	117.76	0.18	117.97	0.17	117.78	0.16	0.31	0.22	0.36
**Y Cγ**	130.75	130.64	130.70	130.76	132.00	0.28	130.43	0.56	132.04	0.31	-1.25	0.21	-1.28
**Y Cδ**	133.05	133.16	133.15	133.10	133.12	0.31	133.05	0.20	133.13	0.24	-0.07	0.11	-0.03
**Y Cζ**	157.27	157.31	157.35	157.33	160.10	0.25	160.43	0.20	160.18	0.27	-2.83	-3.12	-2.85
**R Cζ**	159.49	159.47	159.53	159.51	160.41	0.18	159.57	0.17	159.73	0.29	-0.92	-0.10	-0.22
**GY CO**	174.07	174.00	174.04	174.07	173.19	0.75	173.11	0.68	173.23	0.64	0.88	0.89	0.84
**GR CO**	174.21	174.14	174.22	174.22	173.36	0.78	173.33	0.75	173.67	0.49	0.85	0.81	0.55
**GQ CO**	174.39	174.34	174.39	174.39	173.78	0.71	173.67	0.73	173.78	0.85	0.61	0.67	0.61

**GG CO**	174.63	174.58	174.66	174.66	174.19	0.47	174.13	0.61	174.24	0.52	0.44	0.45	0.42
**GG CO**	174.76	174.69	174.75	174.76							0.57	0.56	0.52
**Q CO**	176.45	176.39	176.45	176.48	176.11	0.66	176.05	0.59	176.11	0.66	0.34	0.34	0.37
**Q/Y CO****	176.72	176.65	176.70	176.74	175.78	0.63	175.67	0.66	175.78	0.80	0.94	0.98	0.96
**R CO**	177.07	177.10	177.17	177.09	175.90	0.60	176.04	0.67	175.92	0.71	1.17	1.06	1.17
**A CO**	178.38	178.32	178.39	178.40	176.74	0.91	176.72	0.86	176.68	0.92	1.64	1.60	1.72
**Q Cδ**	180.47	180.39	180.47	180.49	179.35	0.29	179.65	0.33	179.65	0.35	1.12	0.74	0.84

*Averaged Cβ shifts from experimental data. **Used Y shifts from SHIFTX2 calculation

Table S12. Comparison of predicted and experimental ^{1}H chemical shifts (ppm) for simulated 116-residue MaSp1 hexamer assemblies. The chemical shift difference (Δ) between experiment and models is shown.

Residue	Experimental				SHIFTX2						Δ		
	Intact	Condensed	Dilute	1 M urea	NaCl		Phosphate		Urea		Intact	Condensed	1 M urea
					Average	SD	Average	SD	Average	SD			
**A Hβ**	1.40	1.42	1.39	1.40	1.51	0.18	1.51	0.17	1.51	0.16	-0.11	-0.10	-0.11
**R Hγ**	1.60	1.62	1.60	1.60	1.53	0.12	1.50	0.19	1.47	0.19	0.07	0.12	0.13
**Q Hβ1**	2.15	2.17	2.14	2.15	2.03	0.15	2.05	0.09	2.03	0.11	0.12	0.12	0.12
**Q Hβ2**	1.98	1.99	1.97	1.98	1.96	0.14	2.00	0.11	2.00	0.15	0.02	-0.01	-0.02
**R Hβ1**	1.90	1.91	1.89	1.90	1.85	0.09	1.83	0.11	1.76	0.24	0.05	0.08	0.14
**R Hβ2**	1.74	1.76	1.74	1.75	1.71	0.10	1.70	0.20	1.63	0.24	0.03	0.06	0.12
**Q Hγ**	2.34	2.35	2.33	2.34	2.31	0.10	2.31	0.09	2.31	0.14	0.03	0.04	0.03
**Y Hβ1**	3.06	3.07	3.04	3.06	2.88	0.21	2.94	0.11	2.93	0.16	0.18	0.13	0.13
**Y Hβ2**	2.92	2.93	2.91	2.93	2.79	0.19	2.76	0.16	2.81	0.16	0.13	0.17	0.12
**R Hδ**	3.17	3.18	3.16	3.18	3.12	0.13	3.09	0.20	3.07	0.20	0.05	0.09	0.11
**G Hα**	4.07	4.09	4.07	4.08	3.97	0.22	3.95	0.19	3.95	0.18	0.10	0.14	0.13
**G Hα**	3.95	3.97	3.95	3.96							-0.02	0.02	0.01
**A Hα**	4.31	4.33	4.31	4.32	4.38	0.31	4.43	0.25	4.49	0.26	-0.07	-0.10	-0.17
**A Hα**	4.24	4.25	4.23	4.25							-0.14	-0.18	-0.25
**Q Hα**	4.35	4.36	4.34	4.35	4.38	0.27	4.42	0.25	4.39	0.25	-0.03	-0.06	-0.04
**R Hα**					4.59	0.25	4.35	0.41	4.46	0.28	-0.24	0.01	-0.11
**Y Hα**	4.62	4.63	4.61	4.62	4.80	0.34	4.85	0.42	4.78	0.36	-0.18	-0.22	-0.16
**Y Hα**	4.51	4.53	4.51	4.52							-0.29	-0.32	-0.26
**Y Hε**	6.80	6.83	6.81	6.82	6.69	0.11	6.68	0.11	6.69	0.11	0.11	0.15	0.13
**Y Hδ**	7.09	7.10	7.09	7.10	6.91	0.14	6.92	0.14	6.93	0.12	0.18	0.18	0.17

Table S13. Comparison of ^{13}C solid-state NMR chemical shifts at room temperature and at DNP temperatures (100 K). [a]Peaks from Jenkins et al. *Biomacromolecules,* 2013.(35)

Residue	Shift (ppm) at RT	Shift (ppm) at DNP Temp
[a]P Cα	62.4	62.3
[a]P Cβ	31.6	32.5
[a]P Cγ	26.9	27.7
[a]P Cδ	49.3	49.6
[a]P CO	175.8	177.3
Y Cα	56.6/58.5	57.3
Y Cβ	41.1/39.7	40.3
Y Cγ,δ	133.3	133.7
Y Cε	118.0	117.6
Y Cζ	158.0	157.8
Y CO	175.6/176.7	176.6
[a]A Cα (β-sheet/RC)	50.6/52.8	51.1/53.1
[a]A Cβ (β-sheet/RC)	22.4/18.4	22.9/18.5
[a]A CO (β-sheet/RC)	176.2/180.1	176.3/178.4
[a]G Cα	45.0	45.2
[a]G CO	172.9	174.3 (broad)
[a]Q Cα	54.4	55.7
[a]Q Cβ	29.4	30.1
[a]Q Cγ	32.9	33.9
Q Cδ	179.3	179.7
Q CO	175.2	174.8
R Cα	54.6/55.8/57.2	55.0

**R Cβ**	33.3/30.9	32.7
**R Cγ**	27.9	28.2
**R Cδ**	43.0	43.1
**R Cζ**	159.0	159.0
**R CO**	173.4/175.4/176	175.8

Table S14. ^{13}C chemical shifts (ppm) measured for the silk fiber, compared to chemical shifts for intact glands, condensed phase, and 1 M urea samples. Δ is reported with respect to the fiber (SSNMR – Solution NMR). *Y and Q CO peaks overlap. **R and Q Ca peaks overlap. [a]Peaks from Jenkins et al. *Biomacromolecules*, 2013.(35) Averaged Cβ shifts from solution NMR experimental data. Literature secondary structure chemical shifts are also shown.(33)

Residue	SS NMR	Solution NMR				Δ				Secondary Structure		
		Intact	Condensed	Dilute	1 M urea	Intact	Condensed	Dilute	1 M urea	β-strand	Random coil	α-helix
Y Cα, β-sheet	56.6	57.58	57.66	57.84	57.61	-1.0	-1.1	-1.2	-1.0	56.56	57.72	61.07
Y Cα, RC	58.5					0.9	0.8	0.7	0.9			
Y Cα, β-sheet	56.6	58.15	58.22	58.45	58.18	-1.6	-1.6	-1.9	-1.6			
Y Cα, RC	58.5					0.4	0.3	0.0	0.3			
Y Cβ, β-sheet	41.1	38.64	38.74	38.80	38.69	2.5	2.4	2.3	2.4	40.79	38.71	38.38
Y Cβ, RC	39.7					1.1	1.0	0.9	1.0			
Y Cγ	133.3	130.75	130.64	130.70	130.76	2.6	2.7	2.6	2.5			
Y Cδ		133.05	133.16	133.15	133.10	0.3	0.1	0.2	0.2			
Y Cε	118.0	118.07	118.19	118.25	118.14	-0.1	-0.2	-0.3	-0.1			
Y Cζ	158.0	157.27	157.31	157.35	157.33	0.7	0.7	0.7	0.7			
Y CO, β-sheet	175.6	176.72*	176.65*	176.70*	176.74*	-1.1	-1.1	-1.1	-1.1	174.65	175.32	177.05
Y CO, RC	176.7					0.0	0.0	0.0	0.0			
R Cα, β-sheet	54.6					-1.35	-1.4	-1.4	-1.4			
R Cα, RC	55.8	55.95**	56.03**	56.04**	55.98**	-0.2	-0.2	-0.2	-0.2	54.63	55.96	59.05
R Cα. RC	57.2					1.3	1.2	1.2	1.2			
R Cβ, β-sheet	33.3	30.57	30.69	30.78	30.65	2.7	2.6	2.5	2.7	32.36	30.53	30.00
R Cβ, RC	30.9					0.3	0.2	0.1	0.3			
RCγ	27.9	26.99	27.09	27.16	27.03	0.9	0.8	0.7	0.9			
R Cδ	43.1	43.20	43.25	43.32	43.25	-0.1	-0.1	-0.2	-0.1			
R Cζ	159.0	159.49	159.47	159.53	159.51	-0.5	-0.5	-0.5	-0.5			
R CO, β-sheet	173.4	177.07	177.10	177.17	177.09	-3.7	-3.7	-3.8	-3.7	175.04	175.91	178.11
R CO, β-sheet	175.4					-1.7	-1.7	-1.8	-1.7			

**R CO. RC**	176.0					-1.1	-1.1	-1.2	-1.1			
**A Cα, β-sheet**	50.6[a]	52.67	52.72	52.75	52.68	-2.1	-2.1	-2.2	-2.1	50.86	52.67	54.86
**A Cα, β-sheet**		53.00	53.03	53.09	52.86	-2.4	-2.4	-2.5	-2.3			
**A Cβ, β-sheet**	22.4[a]	18.89	18.97	19.04	18.98	3.5	3.4	3.4	3.4	21.72	19.03	18.27
**A CO β-sheet**	176.3	178.38	178.32	178.39	178.40	-2.1	-2.0	-2.1	-2.1	175.30	177.39	179.58
**G Cα, β-sheet**	45.0[a]	44.53	44.57	44.54	44.55	0.5	0.4	0.5	0.5	45.08	45.34	47.02
**G Cα, β-sheet**		45.29	45.35	45.44	45.29	-0.3	-0.4	-0.4	-0.3			
**GY CO**		174.07	174.00	174.04	174.07	-1.2	-1.1	-1.1	-1.2			
**GR CO**		174.21	174.14	174.22	174.22	-1.3	-1.2	-1.3	-1.3			
**GQ CO**	172.9	174.39	174.34	174.39	174.39	-1.5	-1.4	-1.5	-1.5	173.01	174.30	176.31
**GG CO**		174.63	174.58	174.66	174.66	-1.7	-1.7	-1.8	-1.8			
**GG CO**		174.76	174.69	174.75	174.76	-1.9	-1.8	-1.8	-1.9			
**P Cα**	62.4	63.49	63.53	63.52	63.52	-1.1	-1.1	-1.1	-1.1	62.79	63.53	65.52
**P Cβ**	31.6	31.94	32.07	-	32.01	-0.3	-0.5		-0.4	32.45	31.87	31.08
**P Cδ**	49.3	49.67	49.74	49.86	49.71	-0.4	-0.4	-0.6	-0.4			
**P Cγ**	26.9	27.07	27.13	27.16	27.10	-0.2	-0.2	-0.3	-0.2			
**P CO**	175.8									176.41	176.91	178.34
**Q Cα**	54.4[a]	55.95**	56.03**	56.04**	55.98**	-1.55	-1.6	-1.6	-1.6	54.33	55.94	58.61
**Q Cβ**	29.4[a]	29.28	29.34	29.38	29.32	0.1	0.1	0.0	0.1	31.92	28.67	28.33
**Q Cγ**	32.9[a]	33.70	33.80	33.90	33.74	-0.8	-0.9	-1.0	-0.8			
**Q Cδ**	179.3	180.47	180.39	180.47	180.49	-1.2	-1.1	-1.2	-1.2			
**QCO**	175.2	176.45*	176.39*	176.45*	176.48*	-1.3	-1.2	-1.3	-1.3	174.58	175.88	178.35
**Q CO**	174.8[a]					-1.6	-1.6	-1.6	-1.7			
**S Cα, β-sheet**	56.6[a]	58.58	58.73		58.60	-2.0	-2.1		-2.0	57.14	58.35	60.86
**S Cβ, β-sheet**	66.0[a]	63.82	63.87	63.83	63.86	2.2	2.1	2.2	2.1	65.39	63.88	62.81

Table S15. ^{15}N chemical shifts (ppm) measured for the fiber, compared to chemical shifts for intact glands, condensed phase, and 1 M urea samples. Δ is reported with respect to the fiber (SSNMR – Solution NMR). Literature secondary structure chemical shifts are also shown.(33)

Residue	SS NMR	Solution NMR			Δ			Secondary Structure		
		Intact	Condensed	1 M urea	Intact	Condensed	1 M urea	β-strand	Random coil	α-helix
**P**	135.1	134.41	134.41	134.52	0.7	0.7	0.6			
**A**		124.18	124.23	124.13	0.2	0.2	0.3			
G**A**A		123.98	124.02	123.98	0.4	0.4	0.4			
GQGG**A**G	124.4	123.70	123.75	123.74	0.7	0.7	0.7	125.57	123.2	121.65
A**A**G		122.78	122.81	122.94	1.6	1.6	1.5			
A**A**A		122.57	122.65	122.83	1.8	1.8	1.6			
**R**		120.36	120.63	120.43	1.8	1.6	1.8	122.6	120.59	118.99
**R**			120.57			1.6				
G**Y**		120.04	120.09	120.09	2.2	2.1	2.1			
G**Y**	122.2	120.09	120.23	120.28	2.1	2.0	1.9	122.55	120.05	119.67
G**Y**		119.51	119.57	119.58	2.7	2.6	2.6			
A**Y**		118.92	118.93	119.12	3.3	3.3	3.1			
G**Q**G		119.80	119.83	119.68	2.4	2.4	2.5	123.14	119.73	118.59
G**Q**GGAG		119.69	119.76		2.5	2.4				
Y**G**		110.75	110.79	110.80	0.8	0.7	0.7			
Q**G**		109.88	109.99	109.93	1.6	1.5	1.6			
R**G**		109.80	109.86	109.86	1.7	1.6	1.6			
G**G**		108.73	108.89	108.76	2.8	2.6	2.7			
G**G**	111.5	108.60	108.70	108.63	2.9	2.8	2.9	110.19	109.94	107.34
GQG**G**AG		108.41	108.50	108.45	3.1	3.0	3.1			
GQGGA**G**		108.09	108.21	108.14	3.4	3.3	3.4			
A**G**		107.64	107.73	107.74	3.9	3.8	3.8			
A**G**		107.57	107.65	107.68	3.9	3.8	3.8			

**R NƐ**	86.8	84.61	84.65	84.69	2.2	2.1	2.1	
**R Nη**	72.6	71.4	71.6	71.3	1.3	1.1	1.3	
**R Nη**	75.6				4.3	4.1	4.3	

Table S16. DSSP analysis of the hexamer AF3 β-sheet model (**Fig. 6A**). Highly ordered structure with high β-sheet content including A, R and Y assigned β-sheet secondary structure.

Total Amino Acids	330	234	84	36	12
Secondary Structure	G	A	Q	Y	R
β-sheet (E)	43%	87%	61%	100%	100%
Unstructured (U)	41%	11%	7%	0%	0%
Bend (S)	16%	0%	14%	0%	0%
β-bridge (B)	0%	2%	18%	0%	0%

Table S17. Predicted ^{13}C chemical shifts (ppm) from SHIFTX2 for AF3 β-sheet models compared to experimental chemical shifts from SSNMR. The chemical shift difference (Δ) between experiment and models is shown.
[a]Peaks from Jenkins et al. *Biomacromolecules*, 2013.(35)

Residue	Experimental	SHIFTX2						Δ		
		Trimer	SD	Hexamer	SD	Trimer after MD	SD	Trimer	Hexamer	Trimer after MD
Y Cα, β-sheet	56.6	57.3	0.1	56.9	0.4	57.4	0.6	-0.7	-0.3	-0.9
Y Cα, RC	58.5							1.3	1.7	1.1
Y Cβ, β-sheet	41.1	41.0	0.4	41.1	1.1	39.7	1.3	0.1	0	1.4
Y Cβ, RC	39.7							-1.3	-1.4	0.1
Y Cγ	133.3	132.4	0.1	130.7	0.7	132.4	0.2	0.9	2.6	0.9
Y Cδ		133.1	0.12	133.1	0.4	133.3	0.6	0.2	0.2	0.0
Y Cε	118.0	118.0	0.1	118.0	0.1	118.0	0.1	0.0	0.0	0.0
Y Cζ	158.0	160.2	0.3	160.3	0.2	160.2	0.3	-2.2	-2.3	-2.2
Y CO, β-sheet	175.6	175.0	0.6	175.0	0.4	176.2	0.6	0.6	0.6	-0.6
Y CO, RC	176.7							1.7	1.7	0.5
R Cα, β-sheet	54.6	55.8	0.1	55	0.2	56.1	0.9	-1.2	-0.4	-1.5
R Cα, RC	55.8							0.0	0.9	-0.3
R Cα. RC	57.2							1.4	2.3	1.1
R Cβ, β-sheet	33.3	31.9	0.1	33.6	0.6	32.2	0.4	1.4	-0.3	1.1
R Cβ, RC	30.9							-1	-2.7	-1.3
RCγ	27.9	27.2	0.0	27.2	0.3	26.9	0.3	0.7	0.7	1.0
R Cδ	43.1	43.3	0.0	43.4	0.1	43.0	0.3	-0.2	-0.3	0.0
R Cζ	159.0	159.5	0.1	159.6	0.1	159.9	0.4	-0.5	-0.6	-0.9
R CO, β-sheet	173.4	176.4	0.0	175.0	0.3	176.0	0.6	-3.0	-1.6	-2.6
R CO, β-sheet	175.4							-1.0	0.4	-0.6
R CO. RC	176.0							-0.4	1.0	0.0
A Cα, β-sheet	50.6[a]	51.4	0.4	50.9	0.6	51.5	0.9	-0.8	-0.3	-0.9

**A Cβ, β-sheet**	22.4[a]	21.6	1.0	22.3	1.2	20.9	1.3	0.8	0.1	1.5
**A CO β-sheet**	176.3	175.8	0.8	175.3	0.7	176.1	0.9	0.5	1	0.2
**G Cα, β-sheet**	45.0[a]	44.8	0.7	45.1	0.7	45.1	0.6	0.2	-0.1	-0.1
**G CO**	172.9	173.4	0.8	172.8	0.9	173.8	0.7	-0.5	0.1	-0.9
**Q Cα**	54.4[a]	54.7	0.2	54.8	0.9	54.5	0.8	-0.3	-0.4	-0.1
**Q Cβ**	29.4[a]	31.4	0.6	31.7	1.4	30.9	1.6	-2.0	-2.3	-1.5
**Q Cγ**	32.9[a]	33.8	0.2	33.7	0.3	33.7	0.3	-0.9	-0.8	-0.8
**Qδ**	179.3	179.6	0.1	179.3	0.4	179.4	0.1	-0.3	0.0	-0.1
**Q CO**	175.2	175.2	0.7	175.2	0.9	175.6	0.4	0.0	0.1	-0.4
**Q CO**	174.8[a]							-0.4	-0.3	-0.8

Table S18. Predicted ^{15}N chemical shifts (ppm) from SHIFTX2 for AF3 β-sheet models compared to experimental chemical shifts from SSNMR. The chemical shift difference (Δ) between experiment and models is shown.

Residue	Experimental	SHIFTX2						Δ		
		Trimer	SD	Hexamer	SD	Trimer after MD	SD	Trimer	Hexamer	Trimer after MD
**R**		123.6	0.1	121.0	1.2	119.8	1.4	-1.4	1.2	2.4
**Y**	122.2	118.9	0.5	120.1	1.6	118.1	1.1	3.3	2.1	4.1
**Q**		121.1	1.3	120.2	2.1	119.1	2.4	1.1	2	3.1
**G**	111.5	109.5	1.8	109.1	2.6	109.0	2.1	2.0	2.4	2.5
**A**	124.4	124.2	1.4	123.9	1.4	123.5	2.1	0.2	0.5	0.9

Table S19. DSSP quantification of the trimer AF3 β-sheet model (**Fig. 6B**). Intermediate order with high β-sheet content for A and Y but unstructured R.

Total Amino Acids	57	51	12	6	3
Secondary Structure	A	G	Q	Y	R
β-sheet (E)	74%	35%	75%	100%	0%
Unstructured (U)	26%	53%	25%	0%	100%
Bend (S)	0%	12%	0%	0%	0%

Table S20. DSSP quantification of the trimer AlphaFold3 model after MD simulation (**Fig. 6C**). Lower ordered structure, ~50% poly(Ala) β-sheet, other residues primarily unstructured including R.

Total Amino Acids	57	51	12	6	3
Secondary Structure	A	G	Q	Y	R
β-sheet (E)	53%	8%	17%	33%	0%
Unstructured (U)	39%	75%	50%	50%	100%
Bend (S)	2%	4%	0%	0%	0%
Turn (T)	0%	8%	0%	17%	0%
β-bridge (B)	7%	6%	33%	0%	0%

Table S21: Spectral density functions (J) calculated at 800 MHz for individual residues in MaSp1 under different sample states. Values are given in nanoseconds (ns). Ratios J(0)/J(ωH) and J(0)/J(ωN) are included as indicators of relative motion amplitudes.

**Residue**	**State**	**J(0) [ns]**	**J(ωN) [ns]**	**J(ωH) [ns]**	**J(0)/J(ωH)**	**J(0)/J(ωN)**
A**A**A	condensed	0.394	0.118	0.0164	24.1	3.3
A**A**A	intact	0.435	0.123	0.0168	26.0	3.5
A**A**A	urea	0.315	0.106	0.0189	16.6	3.0
A**A**G	condensed	0.432	0.112	0.0177	24.3	3.8
A**A**G	intact	0.49	0.122	0.0198	24.8	4.0
A**A**G	urea	0.379	0.11	0.0226	16.8	3.4
A**G**	condensed	0.633	0.102	0.0182	34.7	6.2
A**G**	intact	0.306	0.112	0.0201	15.2	2.7
A**Y**	condensed	0.544	0.13	0.0173	31.4	4.2
A**Y**	intact	0.561	0.139	0.0151	37.1	4.0
A**Y**	urea	0.325	0.114	0.0197	16.5	2.9
G**A**A	condensed	0.583	0.124	0.0126	46.3	4.7
G**A**A	intact	0.451	0.127	0.0167	27.0	3.6
G**G**	condensed	0.482	0.114	0.0168	28.7	4.2
G**G**	intact	0.305	0.12	0.0194	15.7	2.5
G**Q**G	condensed	0.335	0.121	0.0184	18.2	2.8
G**Q**G	intact	0.331	0.128	0.0176	18.8	2.6
G**Q**GGAG	condensed	0.318	0.118	0.0172	18.5	2.7
G**Q**GGAG	intact	0.316	0.125	0.0198	16.0	2.5
G**Q**GGAG	urea	0.352	0.099	0.0197	17.9	3.6
GQG**G**AG	condensed	0.499	0.116	0.0183	27.4	4.3
GQG**G**AG	intact	0.315	0.121	0.0179	17.6	2.6
GQGG**A**G	condensed	0.39	0.116	0.0191	20.4	3.4
GQGG**A**G	intact	0.336	0.122	0.0197	17.1	2.8
GQGG**A**G	urea	0.53	0.136	0.0236	22.5	3.9
GQGGA**G**	condensed	0.557	0.105	0.019	29.3	5.3
GQGGA**G**	intact	0.278	0.113	0.0205	13.5	2.5
G**Y**	condensed	0.437	0.133	0.0155	28.1	3.3
G**Y**	intact	0.46	0.143	0.0159	28.9	3.2
G**Y**	urea	0.324	0.123	0.0186	17.5	2.6
Q**G**	condensed	0.42	0.118	0.019	22.1	3.6
Q**G**	intact	0.308	0.119	0.0199	15.4	2.6
**R**	condensed	0.402	0.134	0.0153	26.2	3.0
**R**	intact	0.432	0.138	0.0167	25.8	3.1

R**G**	condensed	0.412	0.116	0.0187	22.0	3.5
R**G**	intact	0.333	0.123	0.0192	17.3	2.7
Y**G**	condensed	0.456	0.13	0.0153	29.7	3.5
Y**G**	intact	0.432	0.136	0.0166	26.0	3.2
Average (all sites)	urea	0.392	0.119	0.0208	19.2	3.4
Average (all sites)	condensed	0.451	0.121	0.0173	26.5	3.8
Average (all sites)	intact	0.371	0.127	0.0181	21.3	2.9

SI References

1. F. Jeffery *et al.*, Microdissection of black widow spider silk-producing glands. *J Vis Exp* 10.3791/2382 (2011).
2. D. Onofrei *et al.*, Investigating the Atomic and Mesoscale Interactions that Facilitate Spider Silk Protein Pre-Assembly. *Biomacromolecules* **22**, 3377–3385 (2021).
3. L. E. Kay, D. A. Torchia, A. Bax, Backbone dynamics of proteins as studied by nitrogen-15 inverse detected heteronuclear NMR spectroscopy: application to staphylococcal nuclease. *Biochemistry* **28**, 8972–8979 (1989).
4. F. Delaglio *et al.*, NMRPipe: a multidimensional spectral processing system based on UNIX pipes. *J Biomol NMR* **6**, 277–293 (1995).
5. M. Bieri, E. J. d'Auvergne, P. R. Gooley, relaxGUI: a new software for fast and simple NMR relaxation data analysis and calculation of ps-ns and μs motion of proteins. *Journal of Biomolecular NMR* **50**, 147–155 (2011).
6. N. A. Farrow, O. Zhang, A. Szabo, D. A. Torchia, L. E. Kay, Spectral density function mapping using 15N relaxation data exclusively. *Journal of Biomolecular NMR* **6**, 153–162 (1995).
7. W. Lee, M. Rahimi, Y. Lee, A. Chiu, POKY: a software suite for multidimensional NMR and 3D structure calculation of biomolecules. *Bioinformatics* **37**, 3041–3042 (2021).
8. K. Takegoshi, S. Nakamura, T. Terao, 13C–1H Dipolar-Assisted Rotational Resonance in Magic-Angle Spinning NMR. *Chemical Physics Letters* **344**, 631–637 (2001).
9. K. Takegoshi, S. Nakamura, T. Terao, C13–1H Dipolar-Driven C13–13C Recoupling Without C13 RF Irradiation in Nuclear Magnetic Resonance of Rotating Solids. *The Journal of Chemical Physics* **118**, 2325–2341 (2003).
10. G. P. Holland, M. S. Creager, J. E. Jenkins, R. V. Lewis, J. L. Yarger, Determining Secondary Structure in Spider Dragline Silk by Carbon−Carbon Correlation Solid-State NMR Spectroscopy. *Journal of the American Chemical Society* **130**, 9871–9877 (2008).
11. T. Izdebski, P. Akhenblit, J. E. Jenkins, J. L. Yarger, G. P. Holland, Structure and Dynamics of Aromatic Residues in Spider Silk: 2D Carbon Correlation NMR of Dragline Fibers. *Biomacromolecules* **11**, 168–174 (2010).
12. J. E. Jenkins *et al.*, Solid-state NMR evidence for elastin-like β-turn structure in spider dragline silk. *Chemical Communications* **46**, 6714–6716 (2010).
13. R. S. Thakur, N. D. Kurur, P. K. Madhu, Swept-Frequency Two-Pulse Phase Modulation for Heteronuclear Dipolar Decoupling in Solid-State NMR. *Chemical Physics Letters* **426**, 459–463 (2006).
14. C. R. Morcombe, K. W. Zilm, Chemical shift referencing in MAS solid state NMR. *Journal of Magnetic Resonance* **162**, 479–486 (2003).
15. P. Bertani, J. Raya, B. Bechinger, 15N chemical shift referencing in solid state NMR. *Solid State Nuclear Magnetic Resonance* **61-62**, 15–18 (2014).
16. A. W. Hing, S. Vega, J. Schaefer, Transferred-echo double-resonance NMR. *Journal of Magnetic Resonance (1969)* **96**, 205–209 (1992).
17. A. Lange, S. Luca, M. Baldus, Structural Constraints from Proton-Mediated Rare-Spin Correlation Spectroscopy in Rotating Solids. *Journal of the American Chemical Society* **124**, 9704–9705 (2002).
18. S. Stoll, A. Schweiger, EasySpin, a comprehensive software package for spectral simulation and analysis in EPR. *J Magn Reson* **178**, 42–55 (2006).
19. M. Mirdita *et al.*, ColabFold: making protein folding accessible to all. *Nature Methods* **19**, 679–682 (2022).
20. M. J. Abraham *et al.*, GROMACS: High performance molecular simulations through multi-level parallelism from laptops to supercomputers. *SoftwareX* **1-2**, 19–25 (2015).
21. J. Lee *et al.*, CHARMM-GUI Input Generator for NAMD, GROMACS, AMBER, OpenMM, and CHARMM/OpenMM Simulations Using the CHARMM36 Additive Force Field. *Journal of Chemical Theory and Computation* **12**, 405–413 (2016).

22. S. Jo, T. Kim, V. G. Iyer, W. Im, CHARMM-GUI: a web-based graphical user interface for CHARMM. *J Comput Chem* **29**, 1859–1865 (2008).
23. J. Huang *et al.*, CHARMM36m: an improved force field for folded and intrinsically disordered proteins. *Nature Methods* **14**, 71–73 (2017).
24. W. G. Hoover, Canonical dynamics: Equilibrium phase-space distributions. *Physical Review A* **31**, 1695–1697 (1985).
25. S. Nosé, A molecular dynamics method for simulations in the canonical ensemble. *Molecular Physics* **52**, 255–268 (1984).
26. S. Nosé, M. L. Klein, Constant pressure molecular dynamics for molecular systems. *Molecular Physics* **50**, 1055–1076 (1983).
27. M. Parrinello, A. Rahman, Polymorphic transitions in single crystals: A new molecular dynamics method. *Journal of Applied Physics* **52**, 7182–7190 (1981).
28. N. Michaud-Agrawal, E. J. Denning, T. B. Woolf, O. Beckstein, MDAnalysis: a toolkit for the analysis of molecular dynamics simulations. *J Comput Chem* **32**, 2319–2327 (2011).
29. R. J. Gowers *et al.* (2016) MDAnalysis: A Python Package for the Rapid Analysis of Molecular Dynamics Simulations. in *Proceedings of the 15th Python in Science Conference*, eds S. Benthall, S. Rostrup (Austin, TX), pp 98–105.
30. J. Abramson *et al.*, Accurate structure prediction of biomolecular interactions with AlphaFold 3. *Nature* **630**, 493–500 (2024).
31. N. A. Ayoub, J. E. Garb, R. M. Tinghitella, M. A. Collin, C. Y. Hayashi, Blueprint for a High-Performance Biomaterial: Full-Length Spider Dragline Silk Genes. *PLOS ONE* **2**, e514 (2007).
32. Anonymous, The PyMOL Molecular Graphics System, Version 3.0 Schrödinger, LLC.
33. Y. Wang, O. Jardetzky, Probability-based protein secondary structure identification using combined NMR chemical-shift data. *Protein Science* **11**, 852–861 (2002).
34. D. S. Wishart, C. G. Bigam, A. Holm, R. S. Hodges, B. D. Sykes, 1H, 13C and 15N random coil NMR chemical shifts of the common amino acids. I. Investigations of nearest-neighbor effects. *J Biomol NMR* **5**, 67–81 (1995).
35. J. E. Jenkins *et al.*, Characterizing the Secondary Protein Structure of Black Widow Dragline Silk Using Solid-State NMR and X-ray Diffraction. *Biomacromolecules* **14**, 3472–3483 (2013).
